# Enantioselectivity-Evaluation of Chiral Copper(II) Complexes Coordinated by Novel Chiral Tetradentate Ligands for Free Amino Acids by Mass Spectrometry Coupled With the Isotopically Labeled Enantiomer Method

**DOI:** 10.3389/fchem.2020.598598

**Published:** 2020-11-30

**Authors:** Takashi Nakakoji, Kaori Yoshino, Kazuki Izutsu, Hirofumi Sato, Hiroyuki Miyake, Eiko Mieda, Satoshi Shinoda, Hiroshi Tsukube, Hideya Kawasaki, Ryuichi Arakawa, Daisuke Ono, Motohiro Shizuma

**Affiliations:** ^1^Department of Chemistry, Graduate School of Science, Osaka City University, Osaka, Japan; ^2^Osaka Research Institute of Industrial Science and Technology, Osaka, Japan; ^3^Faculty of Chemistry, Materials and Bioengineering, Kansai University, Suita, Japan

**Keywords:** chiral metal complex, free amino acid, electrospray ionization mass spectrometry, isotopic labeling, chiral tetradentate ligand, enantioselective complexation

## Abstract

A series of copper(II) complexes with chiral tetradentate ligands, *N,N*′-ethylene- bis(*S*-amino acid methyl amide or methyl ester) prepared from *S-*alanine, *S-*phenylalanine, *S-*valine or *S-*proline, was generated in methanol. The copper complexes provided three component complexes in the presence of a free chiral amino acid. The enantioselectivity for the amino acid was evaluated by electrospray ionization-mass spectrometry coupled with the deuterium-labeled enantiomer method and these copper complexes were found to exhibit high enantioselectivity for free amino acids having bulky side chains. This result suggests that steric interaction between the tetradentate ligand and free amino acid was a major factor in chiral recognition. The copper complex with a chiral tetradentate ligand prepared from *S*-proline showed opposite enantioselectivity to copper complexes consisting of tetradentate ligands prepared from other *S*-amino acids. The conformational difference of the tetradentate ligand in the copper complex was found to be significant for enantioselectivity.

## Introduction

Chiral recognition is one of the essential and fundamental processes in living systems. Particularly, chiral discrimination of α-amino acids and their derivatives has been paid great attention since they are among the most significant compound groups in bioscience. Organometallics and metal complexes can play a special role to detect the chirality of amino acids (Severin et al., 1998; Chin et al., 1999).

For example, chiral lanthanoid complexes were useful to determine enantiomeric excess (*ee*) and the absolute configuration of α-amino acids by using them as nuclear magnetic resonance (NMR) shift reagents (Kabuto and Sasaki, 1987; Takemura et al., 2001). In ligand exchange chromatography using a stationary phase fixed with metal ion such as copper or nickel, enantiomers of chiral amino acids were separated by using a chiral selector as the mobile phase (Brückner et al., 1990). By reversed-phase chromatography, optical separation of chiral amino acids was achieved by using a mobile phase including a chiral selector and copper salts (Gil-Av et al., 1980). Tao et al. used a metal complex with three amino acids including one enantiopure amino acid and two *ee*-unknown amino acids to evaluate the *ee* value of the amino acid with high accuracy by mass spectrometry coupled with collision induced dissociation in the ion trap (Tao et al., 1999, 2000; Tao and Cooks, 2003). Thus, the optical isomerism of chiral amino acids has been examined by various instrumental analysis methods by utilizing the complexation of chiral organometallics or metal complexes with amino acids. However, the details of the complexation and the mechanism of chiral discrimination have not been clarified due to the complicated complexation behaviors in the solution state. Therefore, detailed investigation of complexation of chiral metal complexes with amino acids leads to finding new designs for molecular recognition systems, which can spread to a wide range of fields such as organic, inorganic, analytical, and biological chemistries.

**Figure 1:**
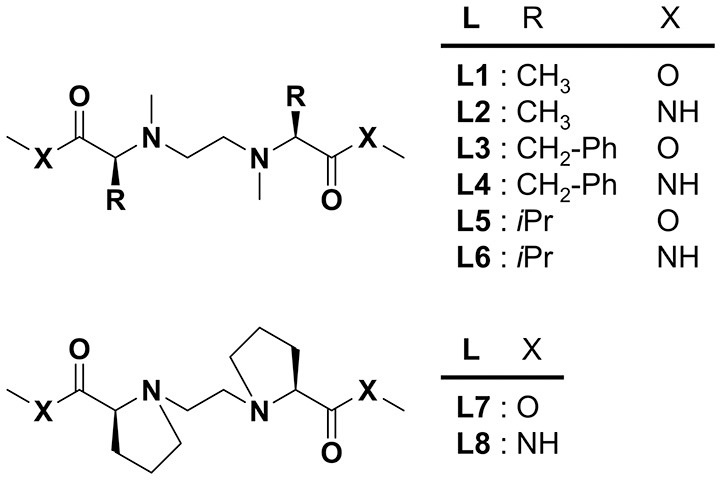
Structures of chiral tetradentate ligands (**L1**–**L8**).

Recently, we have reported the enantioselective coordination of chiral α-amino acids in solution to a copper(II) complex consisting of chiral tetradentate ligand (**L1** or **L2**) (Nakakoji et al., 2020) in which two alanine methyl esters or amides are linked via an ethylene bridge (Miyake et al., 2004). The chiral copper complex produced three-component complex with an amino acid and its enantioselectivity was evaluated by electrospray ionization mass spectrometry coupled with the deuterium-labeled/unlabeled enantiomer guest method. Complex formation behavior observed by spectroscopic methods such as CD and UV, and by DFT calculations. It was clarified that the steric intramolecular interaction between the sidearm of the chiral ligand and that of the amino acid in the three-component complex was the primary factor in the chiral amino acid discrimination by the MS method. It is of interest to clarify the steric factors by altering the steric effects of the side chain of the chiral tetradentate ligand since controlling the enantioselectivity in the coordination of amino acids to the copper(II) complex is one of the significant issues. In this report, we synthesized chiral tetradentate ligands (**L3**–**L8**) with different sidearms in occupancy space size or flexibility (Figure 1) and evaluated the enantioselectivity of amino acid coordination with the copper complexes by electrospray ionization.

## Materials and Methods

### General

^1^H NMR (270 or 300 MHz) and ^13^C NMR (67.5 or 75 MHz) spectra were taken with a JEOL JNM EX-270 FT-NMR spectrometer or a JEOL JNM AL300 FT-NMR spectrometer, respectively. Tetramethylsilane (TMS, δ 0 ppm) was used as the internal standard in CDCl_3_. The solvent signal (δ 4.8 ppm) was used as the internal standard in D_2_O. High resolution mass spectra (ESI, positive ion mode) were measured with a JEOL AccuTOF LC-plus 4G mass spectrometer and the JEOL YOKUDELNA ion peak [M + Na]^+^ (*m/z* 430.9141952) was used as an internal standard for mass calibration. IR spectra were taken with a HORIBA FT-IR 730 in the range of 650–4,000 cm^−1^. Elemental analysis was measured with a CE INSTRUMENTS EA-1110 CHNS-O or J-Science MICRO CORDER JM10. Melting points were measured with a SEIKO DSC SSC/5200. Optical rotation was measured with a Jasco P-1020 with a 10 cm quartz cell irradiating the sodium D line. TLC was performed by using Merck TLC Silica gel 60 F_254_ 25 glass plates detected by a UV lamp (254 nm) or iodine as indicator.

### Materials

Chiral tetradentate ligands having *S*-alanine units (**L1** and **L2**) were synthesized according to previous reports (Miyake et al., 2004). ^1^H NMR and mass spectra of the products are shown in Supplementary Figures 1, 2. The synthesized compounds were purified by column chromatography using silica gel 60 (Merck) and silica gel 60N (Merck) as the stationary phase. Methanol for synthesis was distilled over quicklime as a desiccant.

LC/MS grade methanol (Fujifilm Wako Pure Chem. Co.) was purchased and used for ESI-MS. Ethylene glycol bistosylate was prepared from ethylene glycol and *p-*toluenesulfonyl chloride. Boc-*N*-methyl-*S*-valine methyl amide was prepared by the reported procedures (Bronner et al., 2011; Faggi et al., 2013). All other reagents containing metal salts and amino acids (AAs) were purchased from commercial suppliers and used without further purification. The structures of given deuterium-labeled amino acids (CDN ISOTOPES and ISOTEC, Inc.) are shown in Supplementary Table 1.

### Preparation of Chiral Ligands

#### *N,N*′-Dimethyl-*N,N*′-Ethylene-Bis(S-Phenylalanine Methyl Ester) (L3) (Olsen, 1970; Insaf and Witiak, 1999)

Dimethylformamide (30 mL) was added to a mixture of *N*-Boc-*S*-phenylalanine (2.0 g, 7.5 mmol) and silver oxide (5.2 g, 23 mmol) in an ice bath. Methyl iodide (4.3 g, 30 mmol) was added dropwise to the mixture in the ice bath, and the resulting mixture was stirred at room temperature overnight. The mixture was further stirred at 50°C for 3 h and then cooled to room temperature. The mixture was filtered through celite on the glass filter. The filtrate was mixed with chloroform which was washed with 10% Na_2_S_2_O_3_ aqueous solution and the organic layer was dried over anhydrous sodium sulfate. The solution was evaporated and the residue purified with flash column chromatography (silica gel, ethyl acetate:*n*-hexane = 1:4, v/v) to give *N*-Boc-*N*-methyl-*S*-phenylalanine methyl ester (**1**) as a colorless liquid (1.70 g, 79.0%). ^1^H NMR (270 MHz, CDCl_3_): δ 7.33–7.14 (m, 5H, Ar-*H*), 4.94 (m, 0.5H, C*H*), 4.54 (m, 0.5H, C*H*), 3.74 (s, 6H, OC*H*_3_), 3.29 (m, 1H, C*H*_2_), 3.01 (ddd, ^3^*J* = 11.0 Hz, ^2^*J* = 14.5 Hz, 1H, C*H*_2_), 2.72 (s, 3H, NC*H*_3_), 1.33 (s, 9H, C*H*_3_); ^13^C NMR (67.5 MHz, CDCl_3_): δ 28.2, 35.5, 52.0, 61.6, 128.6, 128.5, 129.0; IR (neat, cm^−1^): 2976, 1745, 1697, 1392, 1171, 1146; elemental analysis, calcd. for C_16_H_23_NO_4_: C, 65.5%; H, 7.9%; N, 4.8%; Found: C, 65.5%; H, 7.9%; N, 4.8%; HRMS (ESI) calculated for C_16_H_22_NO_4_ [M + Na]^+^ *m/z* 316.1525, found *m/z* 316.1542; ${\left[\alpha \right]}_{\text{D}}^{26.5}$ −90.9 (*c* 0.1, CHCl_3_).

Compound **1** (1.0 g, 3.4 mmol) was dissolved in excess TFA, and the solution was stirred for 1 h at room temperature. After the starting material disappeared on TLC (silica gel, ethyl acetate: *n*-hexane = 1:4, v/v), the solution was evaporated to give *N*-methyl-*S*-phenylalanine methyl ester TFA salt. The TFA salt was treated with K_2_CO_3_ aqueous solution. The obtained *N*-methyl-*S*-phenylalanine methyl ester (0.41 g, 2.1 mmol), ethylene glycol bistosylate (0.39 g, 1.1 mmol), and 1,2,2,6,6-pentamethylpiperidine (PMP) (0.39 g, 2.6 mmol) were dissolved in toluene, and the resulting mixture was stirred at 80°C for 4 days. After cooling to room temperature, diethylether was added to the mixture. The precipitate was removed by filtration, the filtrate was evaporated and purified with flash column chromatography (silica gel, ethyl acetate:*n*-hexane = 1:1, v/v) to give **L3** as a colorless liquid (0.26 g, 29.0%). ^1^H NMR (270 MHz, CDCl_3_): δ 7.30–7.15 (m, 5H, Ar-*H*), 3.60 (s, 6H, OC*H*_3_), 3.53 (dd, ^3^*J* = 6.06 Hz, ^2^*J* = 8.57 Hz, 2H, C*H*), 2.97 (ddd, ^3^*J* = 9.15 Hz, ^3^*J* = 13.6 Hz, ^2^*J* = 47.9 Hz, 2H, C*H*_2_), 2.63 (m, 4H, ethylene-C*H*_2_), 2.36 (s, 6H, N-C*H*_3_); ^13^C NMR (67.5 MHz, CDCl_3_): δ 35.9, 38.7, 51.0, 52.8, 68.5, 126.3, 128.3, 129.1, 138.4, 172.2; IR (neat, cm^−1^): 2951, 1732, 1456, 1213, 1196, 1165; elemental analysis, calcd. for C_24_H_32_N_2_O_4_: C, 69.9%; H, 7.8%; N, 6.8%; Found: C, 69.6%; H, 7.7%; N, 6.7%; (ESI) calculated for C_24_H_32_N_2_O_4_ [M + H]^+^ *m/z* 413.2440, found *m/z* 413.2437; ${\left[\alpha \right]}_{\text{D}}^{26.5}$ −52.2 (*c* 0.1, CHCl_3_).

#### *N,N*′-Dimethyl-*N,N*′-Ethylene-Bis(S-Phenylalanine Methyl Amide) (L4)

**L3** (0.10 g, 0.24 mmol) was dissolved in an excess amount of 40% methylamine solution in methanol, and the solution was stirred at 60°C for 2 days and room temperature for a day. The solution was evaporated, and the residue was purified with flash column chromatography (silica gel, chloroform: methanol = 1:9, v/v) to give **L4** as a white solid (0.05 g, 47.2%). ^1^H NMR (300 MHz, CDCl_3_): δ 7.27–7.16 (m, 12H, Ar-*H* and N-*H*), 3.45 (dd, ^3^*J* = 7.0 Hz, ^3^*J* = 7.4 Hz, 2H, -C*H*-CH_2_), 3.32 (dd, ^2^*J* = 7.0 Hz, ^2^*J* = 15.7 Hz, 2H, C*H*H'-Ar), 2.85 (dd, ^3^*J* = 7.4 Hz, ^2^*J* = 15.7 Hz, 2H, CH*H'*-Ar), 2.74 (d, ^3^*J* = 5.3 Hz, 6H, NH-C*H*_3_), 2.56 (m, 2H, -C*H*H'-NCH_3_), 2.45 (m, 2H, -CH*H'*-NCH_3_), 2.25 (s, 6H, N-C*H*_3_); ^13^C NMR (75 MHz, CDCl_3_): δ 26.8, 33.5, 40.4, 52.5, 71.1, 126.8, 129.1, 129.9, 141.0, 173.6; IR (KBr, cm^−1^): 3313, 2979, 1641, 1562, 1454, 1412, 1252, 1120, 1047, 750, 696; elemental analysis, calcd. for C_24_H_34_N_4_O_2_: C, 70.2%; H, 8.4%; N, 13.7%; Found: C, 70.0%; H, 8.4%; N, 13.6%; HRMS (ESI) calculated for C_24_H_34_N_4_O_2_ [M + Na]^+^ *m/z* 433.2579, found *m/z* 433.2582; ${\left[\alpha \right]}_{\text{D}}^{26.6}$ −33.7 (*c* 0.08, CHCl_3_); mp, 101°C.

#### *N,N*′-Dimethyl-*N,N*′-Ethylene-Bis(S-Valine Methyl Ester) (L5)

(*S*)-Valine methyl ester hydrochloride (0.51 g, 3.0 mmol) was dissolved in methanol, and 40% glyoxal aqueous solution was and to the solution. The mixture was stirred at room temperature for 2.5 h. NaBH_3_CN (0.75 g, 11.9 mmol) was added to the mixture keeping the pH at 4.0–5.0 by addition of 30% trimethylamine aqueous solution, and the resulting solution was stirred for 2 weeks. The solution was extracted with chloroform and the organic layer was washed with sat. NaHCO_3_ aq. and dried over anhydrous sodium sulfate. The solution was evaporated, and 0.67 mol/L diethylether hydrochloride was added to gave *N,N*′*-*ethylene-bis(*S*-valine methyl ester) dihydrochloride as a white solid. The solid was purified by recrystallization from chloroform/methanol to yield 0.14 g (16.0%) of *N,N*′*-*ethylene-bis(*S*-valine methyl ester) dihydrochloride. The physical and spectral properties of the product were as follows: ^1^H NMR (270 MHz, D_2_O): δ 3.97 (d, ^3^*J* = 3.92 Hz, 2H, C*H*), 3.78 (s, 6H, OC*H*_3_), 3.40 (s, 4H, C*H*_2_), 2.28 (m, 2H, (CH_3_)_2_-C*H*), 0.95 (dd, ^3^*J* = 6.92 Hz, ^2^*J* = 18.1 Hz, 12H, C*H*_3_); ^13^C NMR (67.5 MHz, D_2_O): δ 18.8, 30.5, 54.3, 67.0; IR (KBr, cm^−1^): 3464, 2954, 2725, 2679, 1743, 1473, 1442, 1234; elemental analysis, calcd. C_14_H_28_N_2_O_4_.4HCl for C, 38.72%; H, 7.43%; N, 6.45%; found C, 38.73%; H, 6.77%; N, 6.35%; HRMS (ESI, positive), calculated for C_14_H_29_N_3_O_4_ (M + H) *m/z* 289.212, found *m/z* 289.212; specific rotation, ${\left[\alpha \right]}_{\text{D}}^{26.5}$ −9.35 (*c* 0.1, H_2_O); mp, 99.5°C.

*N,N*′*-*Ethylene-bis(*S*-valine methyl ester) hydrochloride (0.1 g, 0.28 mmol) was dissolved in methanol, and 37% formaldehyde aqueous solution (0.090 g, 1.1 mmol) was added to the solution, and the mixture was stirred at room temperature at 0.5 h. NaBH_3_CN (0.75 g, 11.9 mmol) was added to the mixture keeping the pH at 4.0 by addition 30% trimethylamine aqueous solution, and the resulting mixture was stirred overnight. The solution was extracted with chloroform and the organic layer was washed with saturated NaHCO_3_ aqueous solution, and dried over anhydrous sodium sulfate. The solution was evaporated and the residue was purified with flash column chromatography (silica gel, ethylacetate: *n*-hexane = 1: 3, v/v) to give **L5** as colorless liquid (0.070 g, 80.0%). The physical and spectral properties of the product were as follows: ^1^H NMR (300 MHz, CDCl_3_): δ 3.68 (s, 6H, OC*H*_3_), 2.78 (d, ^3^*J* = 10.5 Hz, 2H, C*H*), 2.54 (m, 4H, C*H*_2_), 2.26 (s, 6H, N-C*H*_2_), 2.00 (m, 2H, (CH_3_)_2_-C*H*), 0.90 (dd, ^3^*J* = 6.56 Hz, ^2^*J* = 34.3 Hz, 12H, C*H*_3_); ^13^C NMR (75 MHz, CDCl_3_): δ 19.3, 19.8, 27.5, 38.2, 50.4, 52.9, 73.9, 172.5; IR (KB/cm^−1^): 2962, 1732, 1456, 1178, 1149; elemental analysis, calcd. C_16_H_32_N_2_O_4_ C, 60.7; H, 10.2; N, 8.9; found C, 60.4; H, 10.3; N, 8.7; HRMS (ESI, positive) calculated for C_16_H_32_N_2_O_4_ [M + H]^+^ *m/z* 317.2440, found *m/z* 317.2466; specific rotation, ${\left[\alpha \right]}_{\text{D}}^{26.6}$ −78.6 (*c* 0.1, CHCl_3_).

#### N,N′-Dimethyl-N,N′-Ethylene-Bis(S-Valine Methyl Amide) (L6)

A solution of 4N HCl in ethyl acetate (ca. 10 ml) was added to Boc-*N*-methyl-*S*-valine methyl amide (1.03 g, 4.24 mmol) which was stirred for 1 h at room temperature. After evaporation of the solvent, dry diethyl ether was added to the residue to give hydrochloride salt of *N*-methyl-*S*-valine methyl amide as a white solid, which was separated by filtration and dried in vacuo for several hours (yield 0.626 g, 3.48 mmol). To a solution of *N*-methyl-*S*-valine methyl amide hydrochloride (0.626 g, 3.48 mmol) in 20 mL of methanol, 37% formaldehyde aqueous solution (0.252 g, 1.74 mmol) was added, and the mixture was stirred at room temperature for 2.5 h. Ninety percentage NaBH_3_CN (0.253 g, 3.61 mmol) was added to the mixture and the resulting mixture was stirred overnight. The product was extracted with chloroform and the organic layer was washed with saturated NaHCO_3_ aqueous solution three times, and then dried over anhydrous sodium sulfate. The solution was removed and the residue was purified with column chromatography (silica gel, chloroform: methanol = 20:1, v/v) and recycle HPLC (JAIGEL-1H,−2H, chloroform) to give **L6** as a white solid (0.053 g, 9.6%). The physical and spectral properties of the product were as follows: ^1^H NMR (300 MHz, CDCl_3_): δ H 6.55 (brs, 2H, N*H*_2_), 2.82 (d, *J* = 4.8 Hz, 6H, NHC*H*_3_), 2.68 (d, *J* = 6.79 Hz, 2H, C*H*), 2.49 (dd, ^3^*J* = 120.7 Hz, ^2^*J* = 8.57 Hz, 4H, C*H*_2_), 2.25 (s, 6H, N-C*H*_2_), 2.14 (ddd, *J* = 6.98 Hz, 2H, (CH_3_)_2_-C*H*), 0.95 (dd, ^3^*J* = 6.79 Hz, ^2^*J* = 47.1 Hz, 12H, C*H*_3_); ^13^C NMR (75 MHz, CDCl_3_): δ 18.8, 20.9, 26.4, 28.2, 39.7, 54.1, 76.0, 172.8; IR (KBr, cm^−1^): 3300, 3093, 2971, 1639, 1564, 1468, 1412, 1232, 1161, 1109, 1034; elemental analysis, calcd. for C_14_H_34_N_4_O_2_: C, 61.1%; H, 10.9%; N, 17.8%; Found: C, 61.0%; H, 10.9%; N, 17.7%; HRMS (ESI) calculated for C_16_H_34_N_4_O_2_ [M + Na]^+^ *m/z* 337.2579, found *m/z* 337.2604; ${\left[\alpha \right]}_{\text{D}}^{26.6}$ −71.1 (*c* 0.1, CHCl_3_); mp, 168°C.

#### N,N′-Dimethyl-N,N′-Ethylene-Bis(S-Proline Methyl Ester) (L7) (Insaf and Witiak, 1999)

*S*-Proline methyl ester hydrochloride was treated with K_2_CO_3_ aqueous solution. *S*-proline methyl ester (0.50 g, 3.8 mmol), ethylene glycol bistosylate (0.70 g, 0.19 mmol) and PMP (0.71 g, 4.6 mmol) was dissolved in toluene, and the resulting mixture was refluxed for 2 days. After cooling to room temperature, diethylether was added to the mixture. The obtained precipitate was removed by filtration, and the filtrate was evaporated and purified with flash column chromatography (silica gel, chloroform:methanol = 1:9, v/v) to give **L7** as a yellow syrup (0.26 g, 25.0%). ^1^H NMR (300 MHz, CDCl_3_): δ 3.71 (s, 6H, OC*H*_3_), 3.20 (m, 4H, 2H (C*H*), 2H (1-C*H*_2_)), 2.65 (m, 4H, ethylene-C*H*_2_), 2.42 (q, *J* = 8.0 Hz, 2H, 1-C*H*_2_), 2.18–1.72 (m, 8H, 2-C*H*_2_, 3-C*H*_2_); ^13^C NMR (75 MHz, CDCl_3_): δ 23.2, 29.2, 51.7, 53.4, 53.6, 66.0, 174.6; IR (neat, cm^−1^): 2952, 2817, 1734, 1435, 1198, 1171; elemental analysis, calcd. for C_14_H_24_N_2_O_4_0.25CO_2_ C, 58.0%; H, 8.2%; N, 9.5%; Found: C, 57.9%; H, 8.3%; N, 9.3%; HRMS (ESI) calculated for C_14_H_24_N_2_O_4_ [M + H]^+^ *m/z* 285.1814, found *m/z* 285.1849; ${\left[\alpha \right]}_{\text{D}}^{26.6}$ −106.8 (*c* 0.1, CHCl_3_).

#### N,N′-Dimethyl-N,N′-Ethylene-Bis(S-Proline Methyl Amide) (L8)

**L8** was synthesized by the same procedures as **L4**. The product (0.08 g, 80.6%) was afforded from **L7** (0.10 g, 0.35 mmol). The physical and spectral properties of the product were as follows: ^1^H NMR (300 MHz, CDCl_3_): δ H 7.72 (brs, 2H, N*H*), 3.18 (m, 4H, C*H*, 1-C*H*H'), 2.82 (m, 8H, C*H*_3_ and ethylene-C*H*H'), 2.49 (d, *J* = 8.6 Hz, 4H, ethylene-CH*H'*), 2.30 (m, 4H, 1-CH*H'*, 3-C*H*H'), 1.99-1.66 (m, 6H, 2-C*H*_2_, 3-CH*H*'); ^13^C NMR (75 MHz, CDCl_3_): δ24.7, 26.6, 31.0, 54.7, 55.5, 68.6, 175.7; IR (KBr, cm^−1^): 3477, 3305, 2960, 2831, 1836, 1537, 1404, 1302, 1155, 1061, 872, 739; elemental analysis, calcd. for C_14_H_26_N_4_O_2_0.5H_2_O: C, 57.7%; H, 9.3%; N, 19.2%; Found: C, 57.8%; H, 9.3%; N, 19.1%; HRMS (ESI) calculated for C_14_H_26_N_4_O_2_ [M + Na]^+^ *m/z* 305.1953, found *m/z* 305.1977; ${\left[\alpha \right]}_{\text{D}}^{26.6}$ −171.6 (*c* 0.05, CHCl_3_); mp, 122°C.

### Mass Spectrometry

The instrumental conditions for ESI mass spectral measurements (positive ion mode) with a JEOL AccuTOF LC-plus 4G mass spectrometer were optimized to detect the metal complex ions with high sensitivity as follows: voltage of spray needle, 1 kV; orifice1, 50 V; orifice2, 1 V; ring lens, 5 V; temperature of desolvation chamber, 100°C; temperature of orifice1, 50°C; mass range, *m/z* 150–1,000. The mass spectral data were collected under the following conditions: acquisition time, 0.397 s (wait time = 0.003 s, recording time = 0.4 s); measurement time, 2 min.

The accuracy of the 1:1 equivalent of the *R-*AA and deuterium-labeled *S-*AA was calibrated based on the *I*_*R*_/*I*_*S*_ values obtained by the copper complex with achiral tetradentate ligand. *I*_*R*_/*I*_*S*_ measurements of Cu^II^-**L3** and Cu^II^-**L8** were carried out under optimized conditions by controlling the amount of amino acid determined from the relative peak intensity of the complex ions in the mass spectra shown in Supplementary Figures 19–26. *I*_*R*_/*I*_*S*_ measurements of Cu^II^-**L1** was carried out under conditions optimized in a previous report (Nakakoji et al., 2020). The sample solutions used for mass spectral measurements shown in Figure 3 and Supplementary Figures 27–42 were prepared as follows: *solution (1)* 1.20 mL of copper(II) chloride (2.00 × 10^−3^ M) in methanol and 1.00 mL of **L** (2.00 × 10^−3^ M) in methanol were mixed, which was then diluted to 20 mL in a volumetric flask by adding methanol; *solution (2-1)* In the case of Cu^II^-**L3** and Cu^II^-**L8** as chiral hosts, 1.00 mL of solution (1) and 0.1 mL of an equimolar mixture of *R-*AA and deuterium-labeled *S-*AA (5.00 × 10^−4^ M each) containing K_2_CO_3_ (equimolar for carboxyl group) in water were mixed to prepare the final solution (mole ratio: CuCl_2_/(**L**)/*R*-AA/*S*-AA-*d*_n_ = 1.2/1.0/0.5/0.5); (2-2) In the case of Cu^II^-**L1** as chiral host, 1.00 mL of solution (1) and 0.4 mL of an equimolar mixture of *R-*enantiomer and deuterium-labeled *S-*enantiomer (5.00 × 10^−4^ M each) containing K_2_CO_3_ (1.00 × 10^−3^ M) in water were mixed to prepare the final solution (mole ratio: CuCl_2_/(**L**)/*R*-AA/*S*-AA-*d*_n_ = 1.2/1.0/2.0/2.0).

### DFT Calculations

Density Functional Theory (DFT) calculations were performed using a Material Studio version 2018 (Dassault Systems Biovia) on a Windows workstation (Delley, 1990, 2000). The X-ray crystalline structure of copper(II) complex [Cu^II^(**L2**)(MeOH)_2_]^2+^ (Nakakoji et al., 2020) was used as the initial structure and the ligand (**L2**) was modified to build other ligands (**L1**, **L3–L8**) using the Visualizer module in Material Studio. The geometry of each complex [Cu^II^(**L**)(MeOH)_2_]^2+^ in methanol was optimized using a DMol3 module (density functional, B3LYP; basis set, DNP) (Vosko et al., 1980; Lee et al., 1988; Weinert and Davenport, 1992; Becke, 1993; Stephens et al., 1994; Delley, 1995, 2006).

## Results and Discussion

### Detection of Three-Component Complex Ions by Mass Spectrometry

**Figure 2:**
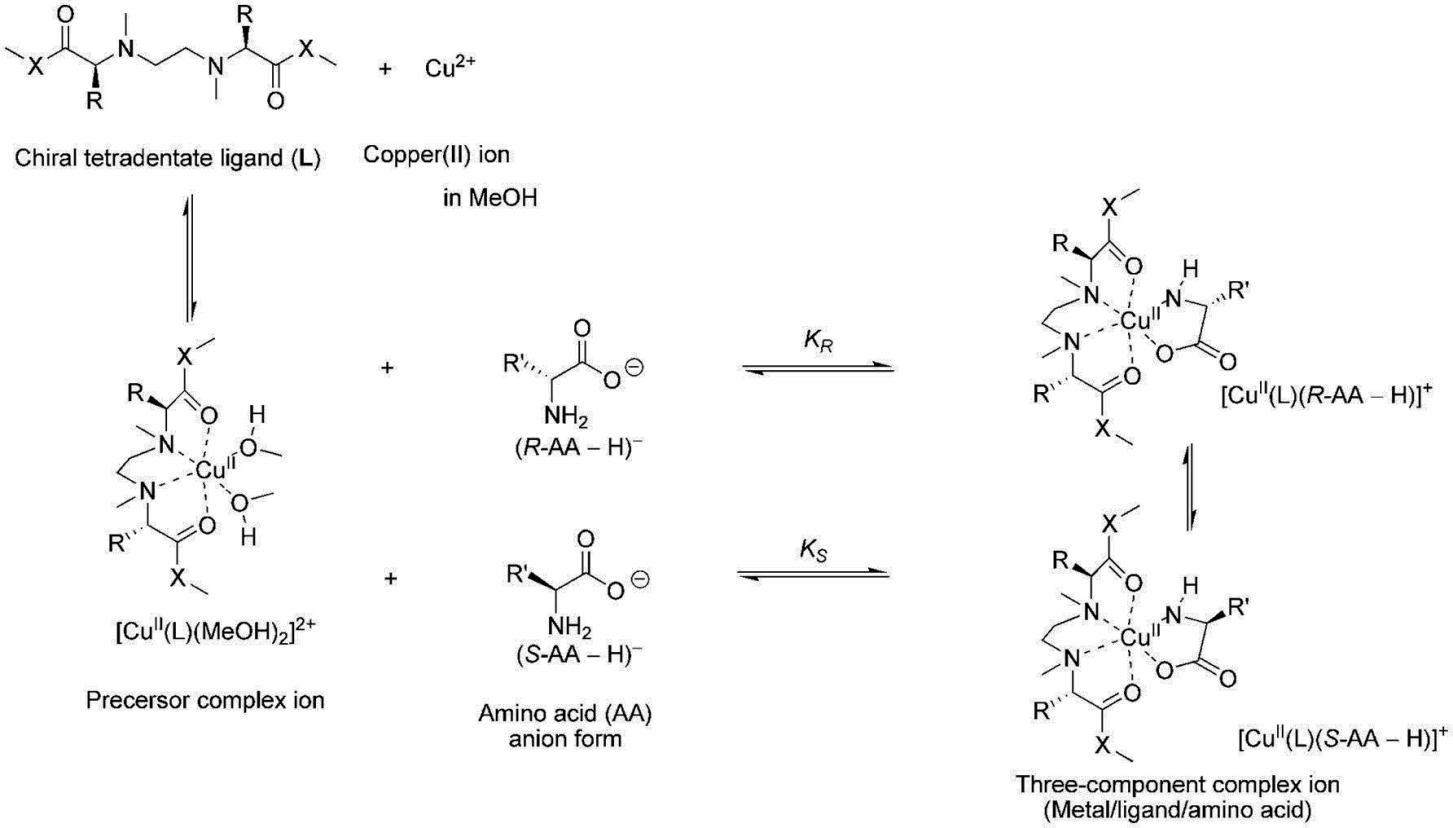
Main process of the enantioselective complexation of the chiral copper complex ions with chiral amino acid.

In this study the ligand exchange reaction of a copper(II) complex ion between chiral amino acids (AAs) in solution was examined (Figure 2). The chiral discrimination abilities of the copper(II) complexes with novel chiral ligands (**L3–L8**) toward free amino acids were evaluated using electrospray ionization mass spectrometry coupled with the deuterium-labeled/unlabeled enantiomer method (the MS/EL method) (Sawada et al., 1995; Sawada, 1997; Shizuma, 2010). In this method, the chiral discrimination ability was estimated on the basis of the relative peak intensity of the three-component complex ions, [Cu^II^(**L**)(*R*-AA–H)]^+^ and [Cu^II^(**L**)(*S*-AA-*d*_n_-H)]^+^ (n, number of deuterium atoms). The complex ions were generated by mixing the copper salt, the chiral ligand, and an equimolar mixture of deuterium-labeled *S-*AA and unlabeled *R-*AA at the following mole ratio: Cu/**L**/*S-*AA-*d*_n_/*R-*AA = 1.2:1.0:2.0:2.0. Addition of excess AA induced competitive coordination of AAs to the precursor complex ion [Cu^II^(**L)**(solvent)_2_]^2+^ (Nakakoji et al., 2020). The relative peak intensity of the complex ions, *I*[Cu^II^(**L**)(*R*-AA–H)]^+^/*I*[Cu^II^(**L**)(*S*-AA-*d*_n_-H)]^+^ (=*I*_*R*_/*I*_*S*_, *I*: peak intensity), under the competitive coordination equilibrium conditions becomes nearly equal to the ratio of association constants in the complexation equilibrium system (*K*_*R*_/*K*_*S*_) (Shizuma et al., 2002, 2005). Copper(II) chloride was chosen as the copper salt since very few peaks of ion species except the three-component complex ions consisting of one chiral ligand **L1** or **L2** were observed in the mass spectra (Nakakoji et al., 2020).

**Figure 3:**
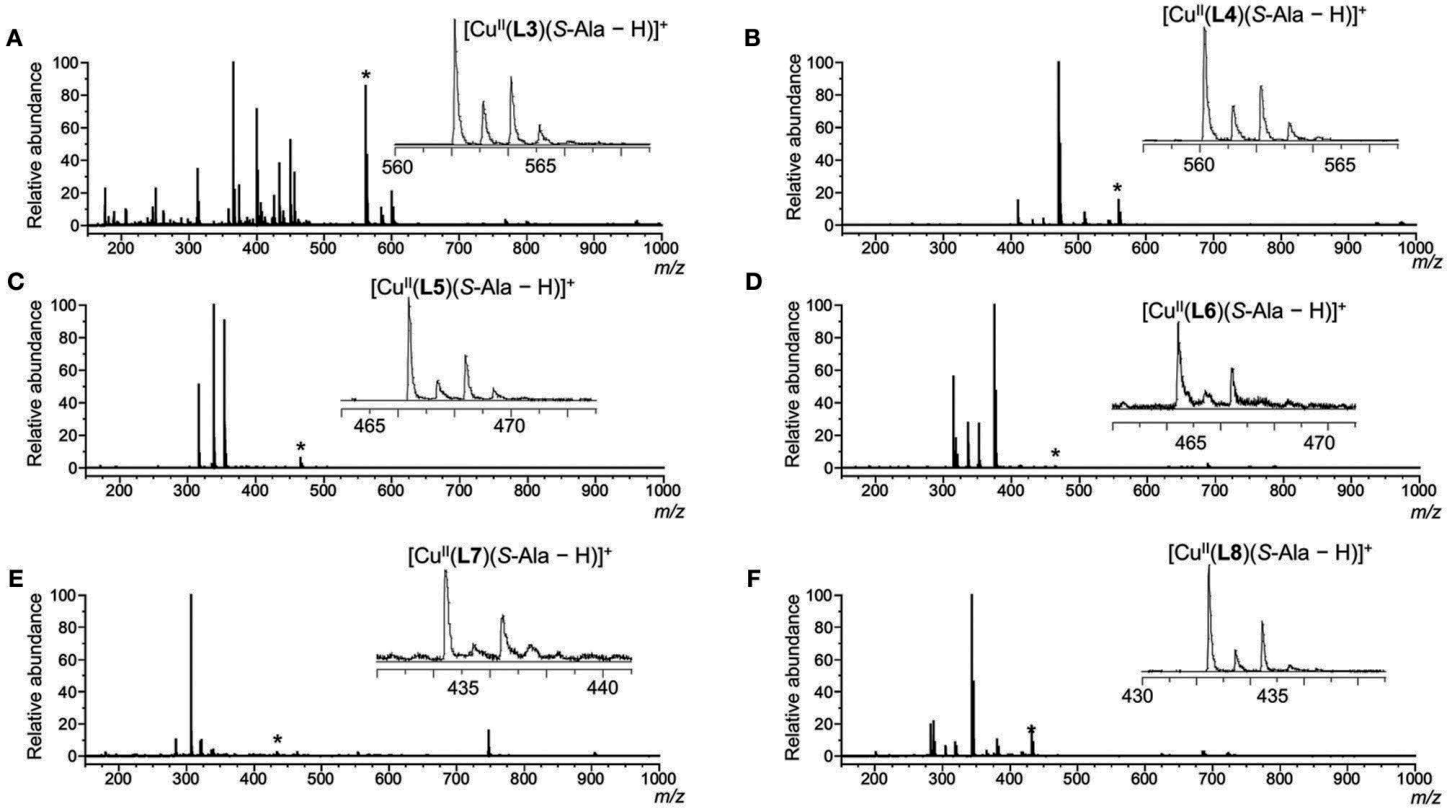
ESI mass spectra of CuCl_2_/**L**/*S*-Ala in water/methanol (1/10, v/v). Each signal marked with an asterisk (*) shows [Cu^II^(**L**)(*S*-Ala – H)]^+^ and the expanded signal is shown in each spectrum. [CuCl_2_]_0_ = 1.09 × 10^−4^ M and [**L**]_0_ = [*S*-Ala]_0_ = [K_2_CO_3_]_0_ = 9.09 × 10^−5^ M. [CuCl_2_]_0_/[**L**]_0_/[*S*-Ala]_0_ = 1.2/1.0/1.0. **L**: **(A) L3**; **(B) L4**; **(C) L5**; **(D) L6**; **(E) L7**; **(F) L8**.

Since detecting the three-component complex ion by mass spectrometry is required to evaluate the chiral discrimination ability, the ion species observed in the mass spectrum were examined. Mass spectra of the mixtures of copper salt, chiral ligands and *S-*alanine are shown in Figure 3. When the pH was <7, the three-component complex ion peak containing coordinated amino acid was not observed. Since the three-component complex ion was observed with high sensitivity by adding potassium carbonate equal to or more than the amino acid, it is required the amino acid in a carboxylate state to form the three-component complex ion. In all chiral ligand cases, the peaks of the three-component complex ions [Cu^II^(**L**)(*S*-AA–H)]^+^ were observed in the mass spectra under the following concentration conditions: [CuCl_2_]_0_, 1.09 × 10^−4^ M; [**L**]_0_, 9.09 × 10^−5^ M; [*S*-Ala]_0_, 9.09 × 10^−5^ M; [K_2_CO_3_]_0_, 9.09 × 10^−5^ M. However, in the cases of Cu^II^/**L5**, Cu^II^/**L6**, and Cu^II^/**L7**, the intensity of the complex ion [Cu^II^(**L**)(*S*-AA–H)]^+^ was very low. It was suggested that the isopropyl units of *S*-valine moieties were too bulky to incorporate *S*-Ala. The three-component complex ions [Cu^II^(**L**)(*S*-AA–H)]^+^ were detected for the Cu^II^/**L3**, Cu^II^/**L4**, and Cu^II^/**L8** systems. The peak intensity of complex ion [Cu^II^(**L3**)(*S*-AA–H)]^+^ (ester type ligand) was higher than that of complex ion [Cu^II^(**L4**)(*S*-AA–H)]^+^ (amide type ligand), and [Cu^II^(**L8**)(*S*-AA–H)]^+^ (amide type ligand) was higher than [Cu^II^(**L7**)(*S*-AA–H)]^+^. Thus, the systems of Cu^II^/**L3** and Cu^II^/**L8** were chosen for further evaluation of the *I*_*R*_/*I*_*S*_ value.

### Evaluation of Chiral Discrimination Ability Toward AA

Tetradentate ligands **L3** and **L8** were chosen for enantioselectivity evaluation of the copper(II) complex toward chiral AAs by mass spectrometry, since three-component complex ions [Cu(**L**)(AA – H)]^+^ were detected with high intensity. Each ligand was mixed with copper(II) chloride in methanol, and then an aqueous solution of an equimolar mixture of a deuterium-labeled *S-*Val (*d*_8_) and unlabeled *R-*Val containing potassium carbonate was added to the methanol solution. The mass spectra of the prepared solutions were measured. In the resulting mass spectra, two complex ions, [Cu(**L**)(*R*-Val – H)]^+^ and [Cu(**L**)(*S*-Val-*d*_8_ – H)]^+^, were observed (Supplementary Figures 19–26). Under competitive complexation conditions with large excess of AA for the copper(II)-**L** complex ([AA]_0_ >> [Cu^2+^(**L**)(MeOH)_2_]_0_), the relative peak intensity of the complex ions (*I*[Cu(**L**)(*R*-Val – H)]^+^/ *I*[Cu(**L**)(*S*-Val-*d*_8_ – H)]^+^ = *I*_*R*_/*I*_*S*_ value, *I*: intensity) was regarded as the chiral discrimination ability. The larger the concentration of Val relative to that of the copper(II)-**L** complex, the larger the relative ratio of *I*_*R*_ and *I*_*S*_ values. Finally, the *I*_*R*_/*I*_*S*_ reached a certain value. In the case where there was a much larger concentration of Val relative to the copper(II)-**L** complex, the signal of [**L** + Na]^+^ was detected as the base peak, suggesting decomposition of copper(II)-**L** complex (Supplementary Figures 22, 26). The final sample preparation conditions were determined based on the effect of the contamination peak and the *I*_*R*_/*I*_*S*_ value. The final concentrations of each component of the sample solution used for the MS measurement were as follows: [CuCl_2_]_0_: [**L**]_0_: [*R*-AA]_0_: [*S*-AA-*d*_n_]_0_: [K_2_CO_3_]_0_ = 1.2 × 10^−4^ M: 1.0 × 10^−4^ M: 5.0 × 10^−4^ M: 5.0 × 10^−4^ M: 1.0 × 10^−3^ M in methanol/water (10/1).

**Figure 4:**
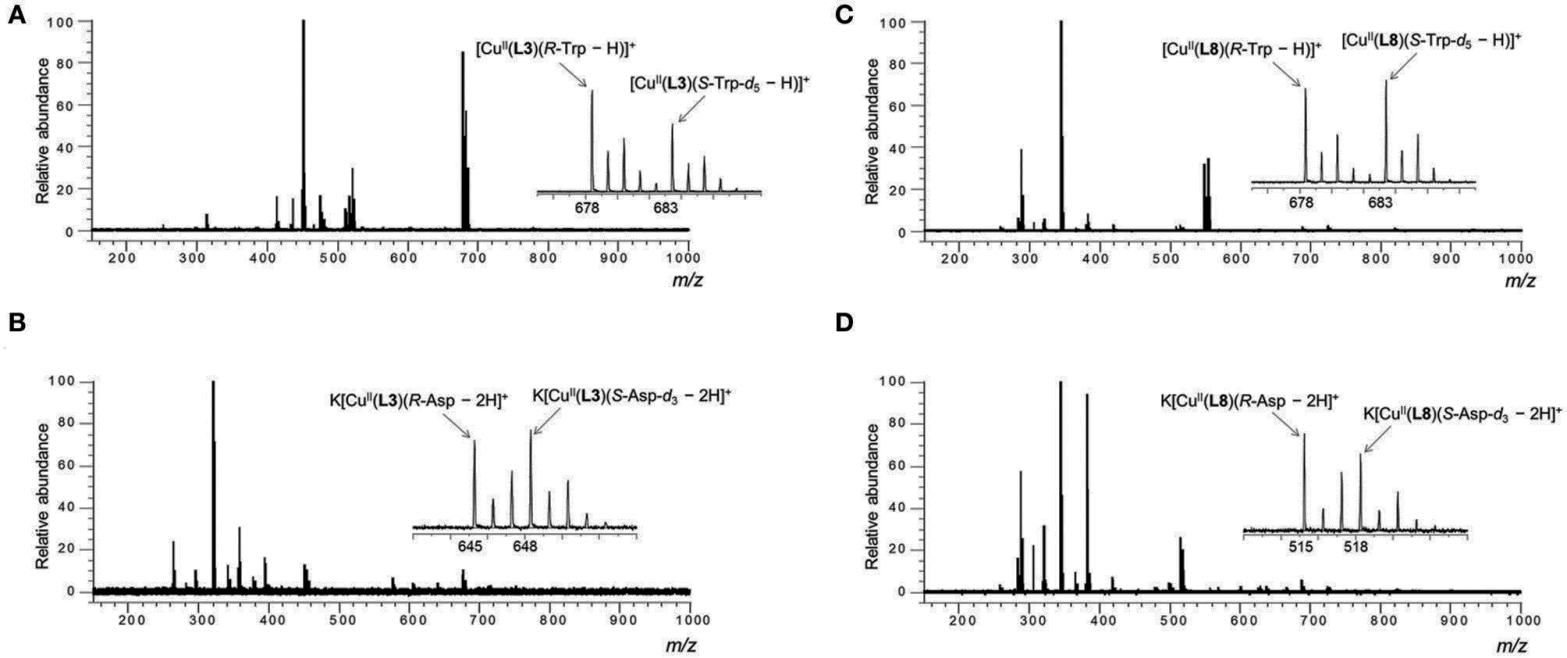
ESI mass spectra of the MS/EL method of CuCl_2_/**L**/*R*-AA/*S*-AA-*d*_n_ in water/methanol (1/10, v/v). [CuCl_2_]_0_ = 1.09 × 10^−4^ M, [**L**]_0_ = 9.09 × 10^−5^ M and [*R*-AA]_0_ = [*S*-AA-*d*_n_]_0_ = 4.55 × 10^−5^ M. [CuCl_2_]_0_/[**L**]_0_/[*R*-AA]_0_/[*S*-AA-*d*_n_]_0_ = 1.2/1.0/0.5/0.5. **(A)** [K_2_CO_3_]_0_ = 9.09 × 10^−5^ M, **L** = **L3**, AA = *R*-Trp/*S*-Trp-*d*_5_, **(B)** [K_2_CO_3_]_0_ = 1.82 × 10^−4^ M, **L** = **L3**, AA = *R*-Asp/*S*-Asp-*d*_3_, **(C)** [K_2_CO_3_]_0_ = 9.09 × 10^−5^ M, **L** = **L8**, AA = *R*-Trp/*S*-Trp-*d*_5_, **(D)** [K_2_CO_3_]_0_ = 1.82 × 10^−4^ M, **L** = **L3**, AA = *R*-Asp/*S*-Asp-*d*_3_.

Sample solutions for various amino acids were prepared under the condition described above, and their mass spectra were measured. Two three-component complex ion peaks were observed in all measured mass spectra. As a typical mass spectrum, the mass spectrum of the sample solution using **L3** as the ligand and Val as the AA is shown in Figure 4A. In the case of Asp, the three-component complex ion peak was detected as a potassium ion adduct K[Cu(**L**)(Asp – 2H)]^+^ (Figure 4B). The mass spectra of other sample solutions are shown in the Supplementary Figures 27–42. As shown in the enlarged view in Figure 4, two complex ion peaks with isotope distributions characteristic of copper were observed. In the case of **L3** and Trp (Figure 4A), the signal intensity on the low mass side was large, suggesting that the *R-*amino acid coordinates with the complex ion preferentially. The *I*_*R*_/*I*_*S*_ value was 1.64. In the case of **L8** and Asp, the *I*_*R*_/*I*_*S*_ value was 1.31 (*R*-preference).

The relative intensity (*I*_*R*_/*I*_*S*_) values of the two complex ions are summarized in Table 1, and the values are corrected for the contribution of the isotope peaks of the complex ions on the low mass side to the peak intensity on the high mass side. The complex containing **L3** showed high chiral discriminating ability for Phe, Val, Hyp, and Trp, and its enantioselectivity preference was the *R-*amino acid. On the other hand, in the complex ions containing **L8**, a high chiral discrimination ability was observed for Hyp and Asp and its enantioselectivity was *S-*selective for Hyp and *R-*selective for Asp.

**Table 1:** *I*_*R*_/*I*_*S*_ values of a Cu^II^-chiral ligand (**L3** and **L8**)-amino acid (AA) complexation system in the MS/EL method.

**Ligand**	**Amino acid (AA)**	***I_R_/I_S_***	**Enantioselectivity**
**L3**	Ala	0.99	–
**L3**	Leu	0.99	–
**L3**	Val	1.35	*R*
**L3**	Met	0.97	–
**L3**	Orn	1.15	*R*
**L3**	Lys	1.15	*R*
**L3**	Phe	1.42	*R*
**L3**	Hyp	2.99	*R*
**L3**	Trp	1.64	*R*
**L3**	Asp	0.96	–
**L8**	Ala	1.02	–
**L8**	Leu	1.05	–
**L8**	Val	0.95	–
**L8**	Met	0.94	*S*
**L8**	Orn	1.06	*R*
**L8**	Lys	1.06	*R*
**L8**	Phe	0.86	*S*
**L8**	Hyp	0.27	*S*
**L8**	Trp	1.04	–
**L8**	Asp	1.31	*R*

As described in previous reports, detailed investigations for copper(II) complex containing **L1** or **L2** as tetradentate ligands and amino acid confirmed that the complexation observed by mass spectrometry took place in the solution, not in the gas phase (Nakakoji et al., 2020). In this study using ligands with similar structures, it seems that the ligand exchange reaction in solution was observed by mass spectrometry.

### Structure of Complex Ions and Enantioselectivity

To clarify the chiral discrimination mechanism, it is necessary to investigate the complex structure. For the complex structure, a spectroscopic approach based on the absorption spectrum or the circular dichroism spectrum is desirable. However, since the complexation equilibrium system of this study contains many ionic species (see Nakakoji et al., 2020), it shows complicated spectral changes. Therefore, it is difficult to evaluate the structure by spectroscopy. Furthermore, since divalent copper ions are paramagnetic, it is difficult to analyze the complex structure by the nuclear magnetic resonance (NMR) method. Therefore, we adopted DFT method for optimization of their structures.

**Figure 5:**
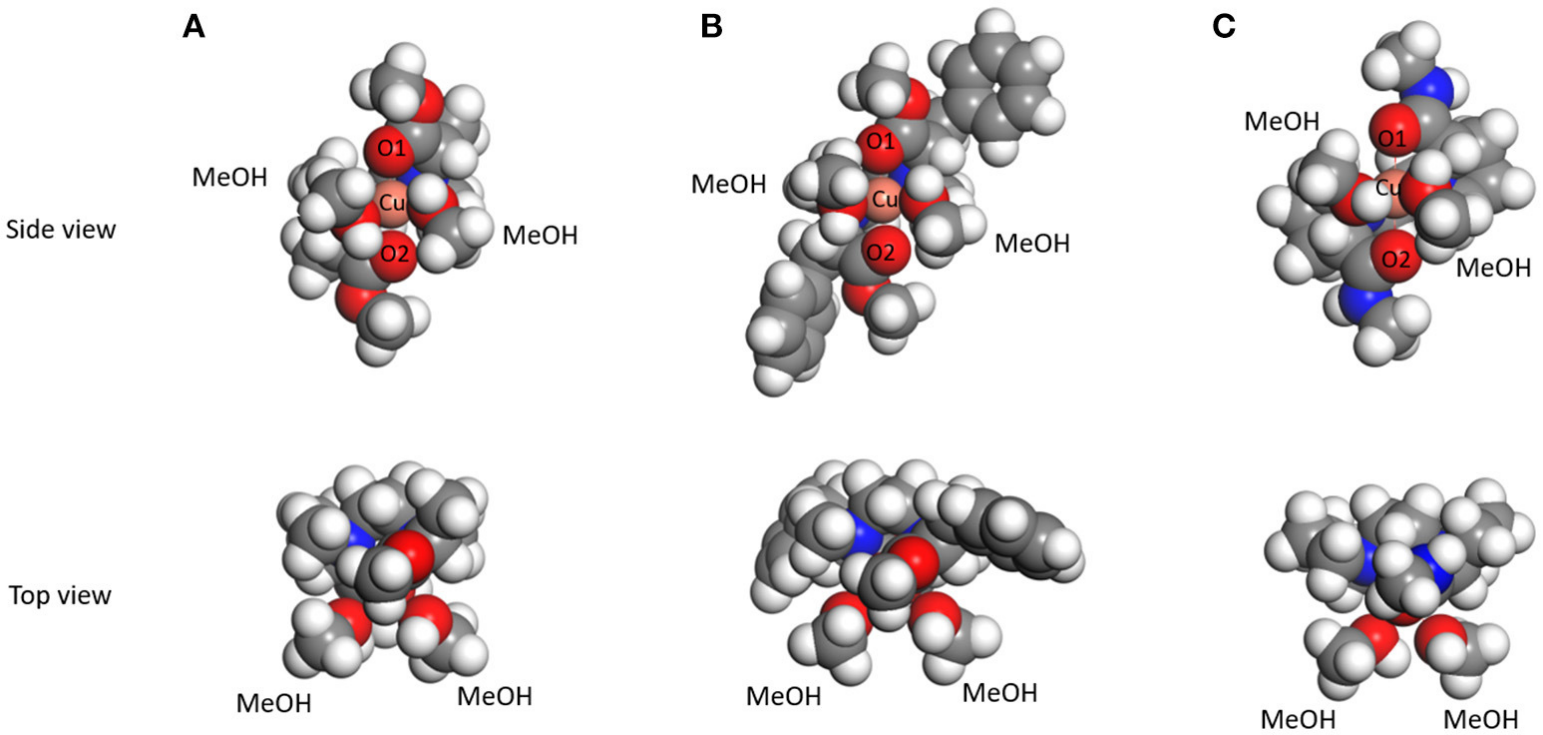
The structures of copper(II)-ligand complexes [Cu^II^(**L**)(MeOH)_2_]^2+^ in methanol optimized by DFT calculations. **(A) L** = **L1**, **(B) L** = **L3**, and **(C) L** = **L8**.

The structure of the complex [Cu(**L**)(MeOH)_2_]^2+^ in methanol, which is the precursor to generate the three-component complex [Cu(**L**)(AA – H)]^+^, was investigated by DFT calculations. The amino acid could occupy the cis position on the copper(II) ion as a bidentate ligand by replacing the two methanol molecules. X-ray crystalline structure analysis of the complex [Cu(**L2**)(MeOH)_2_]^2+^ revealed that the chiral tetradentate ligand is coordinated in *cis-*alpha form (Nakakoji et al., 2020). The initial structures of the complexes for DFT calculations were built based on the crystalline structure. The structures of the complexes [Cu(**L**)(MeOH)_2_]^2+^ (**L** = **L1**, **L3**, and **L8**) obtained by DFT calculations are shown in Figure 5 (The results of **L2**, **L4**–**L7** are shown in Supplementary Figure 43). The complexes [Cu(**L**)(MeOH)_2_]^2+^ (**L**: **L1**–**L8**) had slightly twisted *cis-*alpha structures with *C*_2_-symmetry similar to the complex [Cu(**L2**)(MeOH)_2_]^2+^ whose crystal structure was reported. The bond length between copper ion and the oxygen atom of the carbonyl of the tetradentate ligand in [Cu(**L1**)(MeOH)_2_]^2+^ and [Cu(**L3**)(MeOH)_2_]^2+^ was 2.15 Å (Supplementary Table 2). On the other hand, the bond length (2.5 Å) of Cu-O in [Cu(**L8**)(MeOH)_2_]^2+^ was longer than this. This is considered to be the effect of strain due to the rigid structure of the proline ring in the chiral tetradentate ligand. The side chains of the ligand in the complex ions [Cu(**L1**)(MeOH)_2_]^2+^ and [Cu(**L3**)(MeOH)_2_]^2+^ are free to rotate, whereas this is difficult for the ligand in the complex ion [Cu(**L8**)(MeOH)_2_]^2+^. This structural divergence of the complexes suggests that the complexes [Cu(**L1**)(MeOH)_2_]^2+^ and [Cu(**L3**)(MeOH)_2_]^2+^ provide similar large spaces to coordinate AA, and [Cu(**L8**)(MeOH)_2_]^2+^ provides a different space due to the rigidity of the Pro moieties.

**Figure 6:**
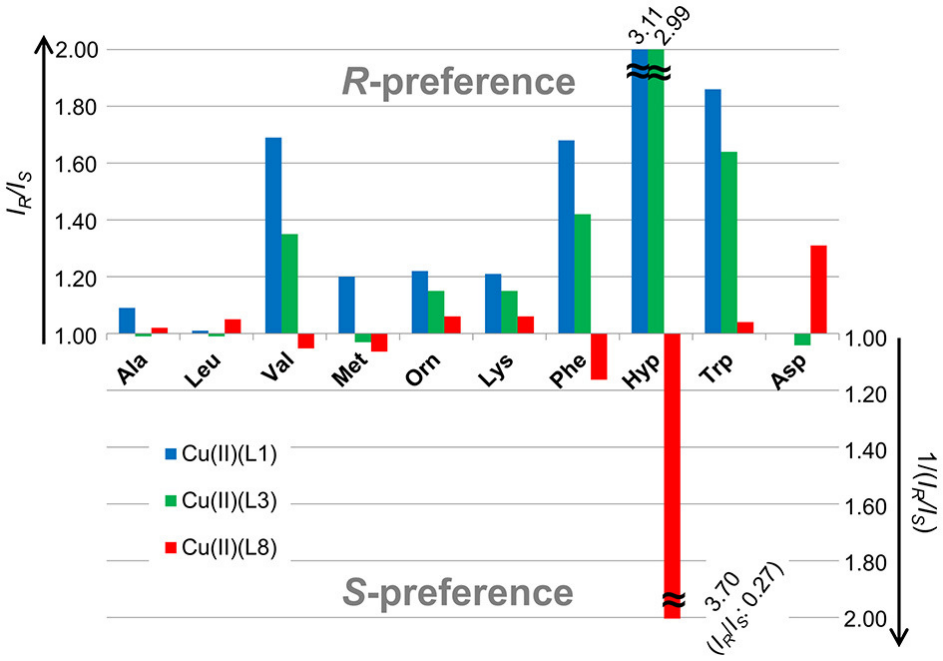
*I*_*R*_/*I*_*S*_ values of a Cu^II^-chiral ligand (**L1**, **L3** and **L8**)-amino acid (AA) complexation system with the MS/EL method.

As shown in Figure 6, the enantioselectivity in the complexation of [Cu(**L1**)(AA – H)]^+^ (blue block) and [Cu(**L3**)(AA – H)]^+^ (green block) with each AA was similar, while that of [Cu(**L8**)(AA – H)]^+^ (red block) was quite different. Thus, it is considered that the slight difference of the coordination structure of the copper(II) complex with the tetradentate ligand, which is calculated by DFT, is the main factor that determines the enantioselectivity for amino acids. The magnitude of the chiral discrimination ability for amino acids is considered to depend on the steric interaction between the side chain of the chiral tetradentate ligand and that of the amino acid. In fact, a large chiral discrimination ability was observed for AAs such as Phe, Val, Hyp, and Trp, which have large side chains.

The selectivity in reactions such as asymmetric catalytic reactions or ligand-exchange of metal complexes was investigated on the basis of the steric parameters (Sigman and Miller, 2009). The Taft steric parameters (Taft, 1952, 1953) have been applied to various fields such as quantitative structure-activity relationship (QSAR) in medicinal chemistry (Unger and Hansch, 1979; Huang et al., 2014). The ordering of the Taft steric parameter (*E*_S_) was –CH_3_ (0) > -CH_2_C_6_H_5_ (−0.38) > -CH(CH_3_)_2_ (−0.47). The ordering shows agreement with that of the intensity of the three-component complex ion [Cu(**L**)(AA – H)]^+^ in mass spectrometry. The coordination of the AA to [Cu(**L**)(MeOH)_2_]^2+^ suggests a dependence on the steric interaction between the side arms of AA and the precursor complex ion.

Thus, the binding ability with AA and the chiral AA discrimination ability of copper(II)- tetradentate ligand complex ions were controlled by the steric effect of the side arms of the ligand.

## Summary

In this paper, we evaluated the chiral discrimination ability in the enantioselective complexation of a copper(II)-chiral tetradentate ligand with a chiral amino acid as a second ligand by electrospray ionization mass spectrometry coupled with the isotopically labeled/unlabeled enantiomer method and clarified the following facts. (i) In the chiral tetradentate ligands **L5** and **L6** where the sidearms are sterically bulky, it is difficult to form the three-component complex with an amino acid. (ii) The complex ions with chiral tetradentate ligands **L1** and **L3**, which coordinate to the copper ion in pseudo *cis-*alpha type fashion, are similar in enantioselectivity toward amino acids. (iii) In the case of ligand **L8**, which has proline rings in the skeleton, the pseudo *cis-*alpha type of structure cannot be maintained due to the rigidity, and the enantioselectivity of the copper complex for amino acids are different from those of the complexes with **L1** or **L3**. In the case of amino acid having bulky side arm such as Val, Phe, Hyp, Cu^II^/**L8** complex showed opposite enantioselectivity to Cu^II^/**L2** complexes. It was clarified that the enantioselectivity for amino acids is controlled by the slight difference in the coordination form of the copper(II) complex with chiral tetradentate ligand, and the magnitude of the chiral discrimination ability depends on the steric interaction between the sidearms of the chiral tetradentate ligand and the side chain of the amino acid.

Mass spectrometry coupled with the isotopically labeled enantiomer method can be applied to a method for determination of the enantiomeric excess of chiral guests by using an isotopically labeled/unlabeled enantiomer pair for the chiral host (Sawada et al., 1998; Shizuma et al., 2000). Remarkably, it was revealed that the copper(II) complex with ligand **L8** has enantioselectivity for Asp, of which racemization in the human body has been reported to be related to various diseases (Man et al., 1983; Fujii et al., 1994; Fujii, 2005; Xin et al., 2007). Therefore, the copper(II)-chiral tetradentate ligand-amino acid three-component complexation equilibrium system proposed in this study can be applied to a method that can determine the optical purity of Asp simply, quickly and with high sensitivity by mass spectrometry alone. The knowledge obtained in this research will contribute to the future technological development of disease diagnosis.

## Data Availability Statement

The datasets generated for this study can be found in online repositories. The names of the repository/repositories and accession number(s) can be found in the article/Supplementary Material.

## Author Contributions

TN, HS, HM, and MS conceived and designed the study. TN, HS, HM, SS, and HT designed the metal-chiral ligand complexes. TN, KY, KI, EM, and HS synthesized and purified them. TN and MS measured the spectral data. HK, RA, and MS conceived the mass spectrometric experiments. TN prepared sample solutions and measured mass spectra. HM and MS performed the molecular simulations. HM, SS, HT, HK, RA, DO, and MS verified and discussed the results.TN, HM, and MS wrote the paper. All authors contributed to the article and approved the submitted version.

## Conflict of Interest

The authors declare that the research was conducted in the absence of any commercial or financial relationships that could be construed as a potential conflict of interest.
