# Supplementary material for: Enantioselectivity-Evaluation of Chiral Copper(II) Complexes Coordinated by Novel Chiral Tetradentate Ligands for Free Amino Acids by Mass Spectrometry Coupled With the Isotopically Labeled Enantiomer Method
Source: Front Chem. 2020 Nov 30;8:598598. doi: 10.3389/fchem.2020.598598 (PMC7793834; doi:10.3389/fchem.2020.598598)
Supplement: Supplementary file 1 [file Data_Sheet_1.docx]

Supplementary Material

Enantioselectivity-evaluation of chiral copper(II) complex coordinated by novel chiral tetradentate ligand for free amino acids by mass spectrometry coupled with the isotopically labeled enantiomer method

Takashi Nakakoji^1^, Hirofumi Sato^2^, Hiroyuki Miyake^1^*, Satoshi Shinoda^1^, Hiroshi Tsukube^1^, Hideya Kawasaki^3^, Ryuichi Arakawa^3^, Daisuke Ono^2^, Motohiro Shizuma^2^*

^1^Department of Chemistry, Graduate School of Science, Osaka City University, Osaka, Japan

^2^Osaka Research Institute of Industrial Science and Technology, Osaka, Japan

^3^Faculty of Chemistry, Materials and Bioengineering, Kansai University, Suita, Japan

*** Correspondence:**Hiroyuki Miyake
miyake@sci.osaka-cu.ac.jp

Motohiro Shizuma
shizuma@omtri.or.jp

Contents

**1 ^1^H-NMR spectra of compounds** (Figures S1–S9)

**2 High resolution mass spectra of compounds** (Figures S10–S18)

**3 ESI mass spectra of the mass spectrometry/enantiomer-labeled (MS/EL) method of CuCl_2_/L3/*R*-Val/*S*-Val-*d_8_* and CuCl_2_/L8/ *R*-Val/*S*-Val-*d_8_* in water/methanol** (Figures S19–S26)

**4 ESI mass spectra of the MS/EL method of CuCl_2_/L/*R*-AA/S-AA-*d_n_* in water/ methanol** (Figures S27–S42)

**5 Deuterated amino acids** (Table S1)

**6 DFT calculation of copper(II)-ligand complex [Cu(L)(MeOH)_2_]^2+^** (Figure S43)

**7 Bond length of Cu-O, Cu-N, and Cu-(MeOH) of copper(II)-ligand complex [Cu(L)(MeOH)_2_]^2+^ by DFT** (Table S2)

**8 Coordinates of copper(II)-ligand complex [Cu(L)(MeOH)_2_]^2+^ by DFT (Tables S3-S4)**

# ^1^H-NMR spectra of compounds


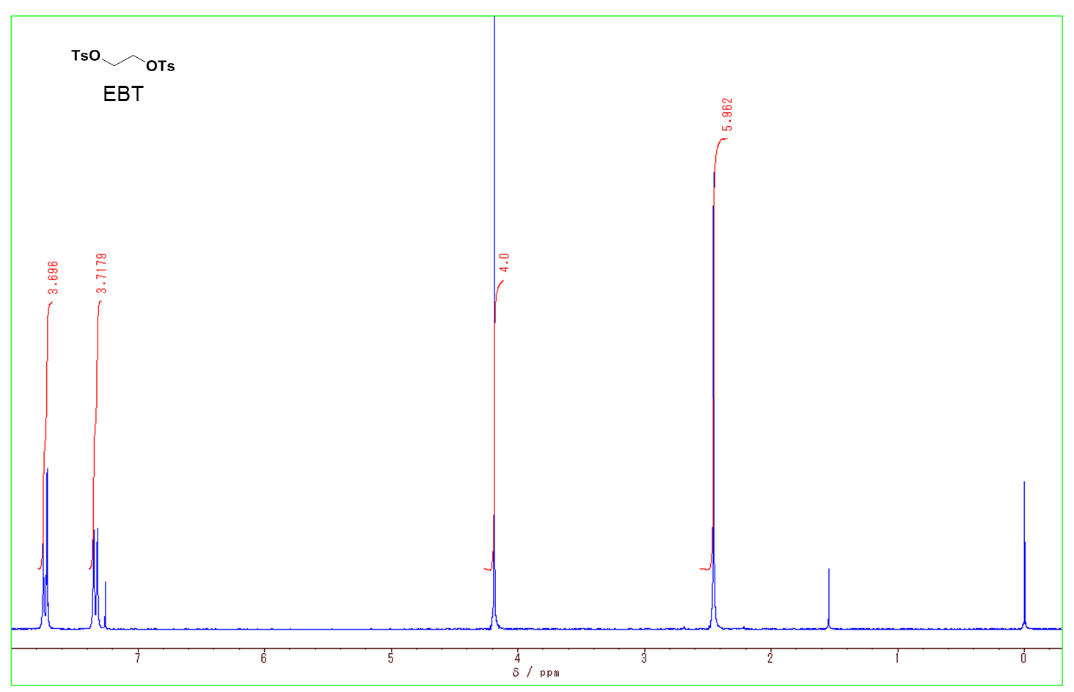


**Figure S1.** ^1^H NMR (270 MHz, CDCl_3_, 293 K) spectrum of ethylene bistsylate


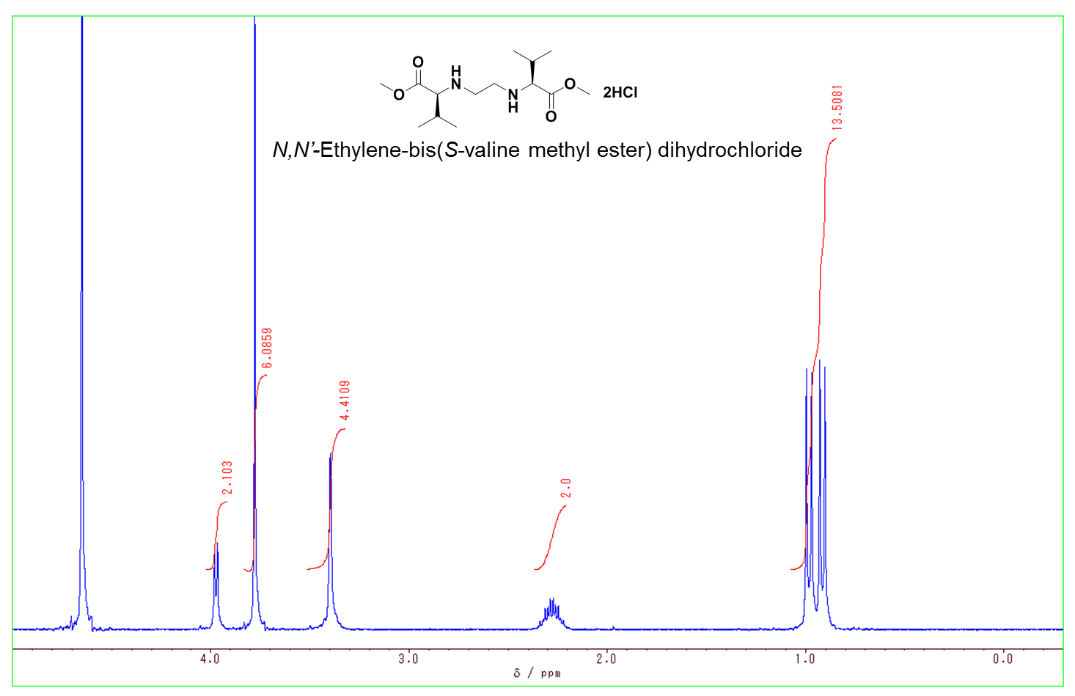


**Figure S2.** ^1^H NMR (270 MHz, CDCl_3_, 293 K) spectrum of *N,N´-*ethylene-bis(*S*-valine methyl ester) dihydrochloride


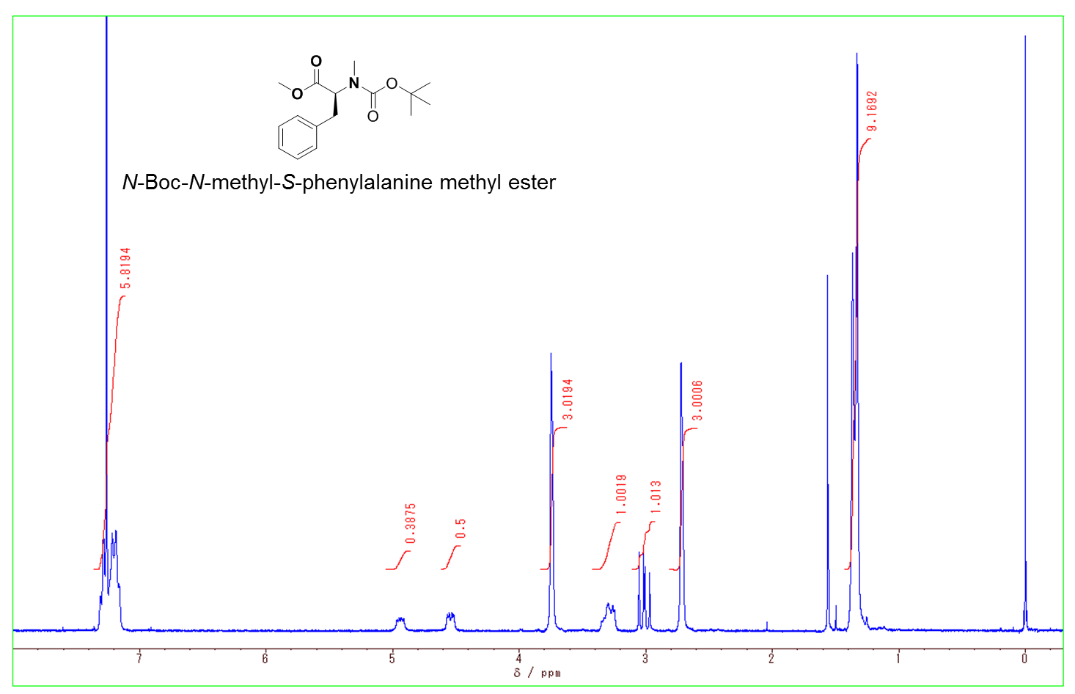


**Figure S3.** ^1^H NMR (270 MHz, CDCl_3_, 293 K) spectrum of *N*-Boc-*N*-methyl-*S*-phenylalanine methyl ester


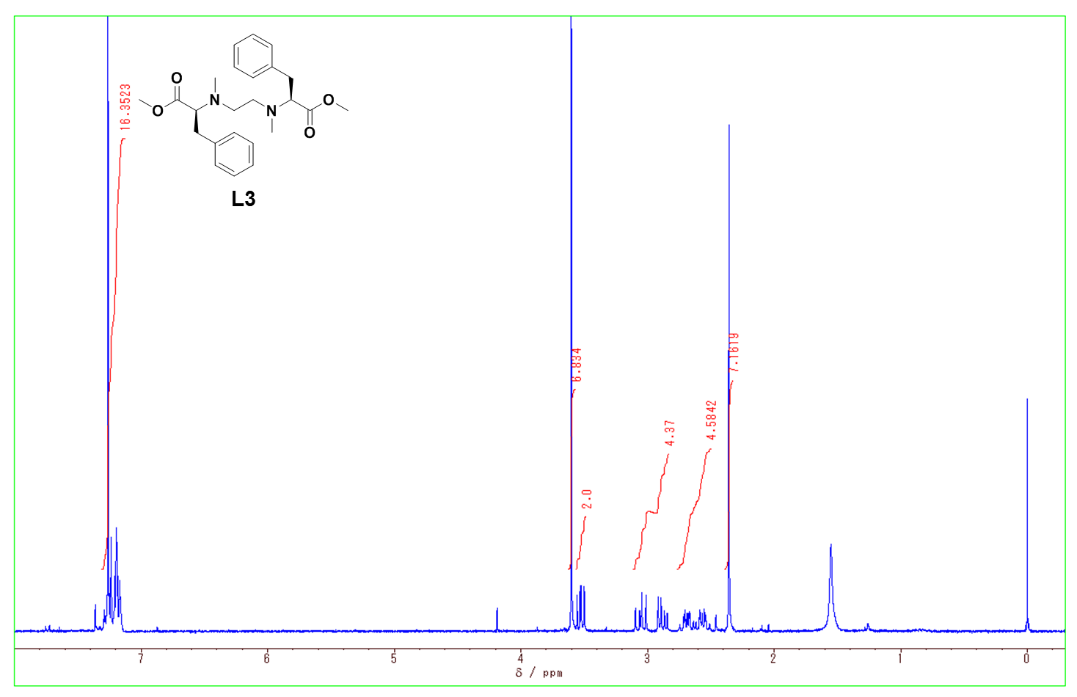


**L3**

**Figure S4.** ^1^H NMR (270 MHz, CDCl_3_, 293 K) spectrum of **L3**


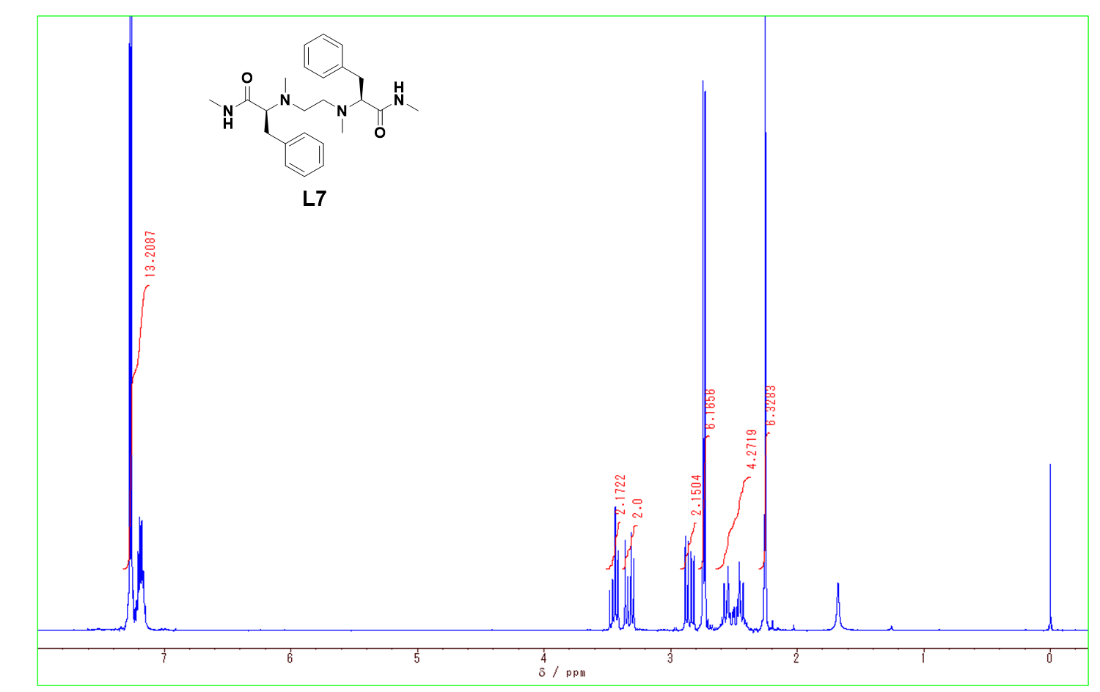


**L4**

**Figure S5.** ^1^H NMR (300 MHz, CDCl_3_, 293 K) spectrum of **L4**


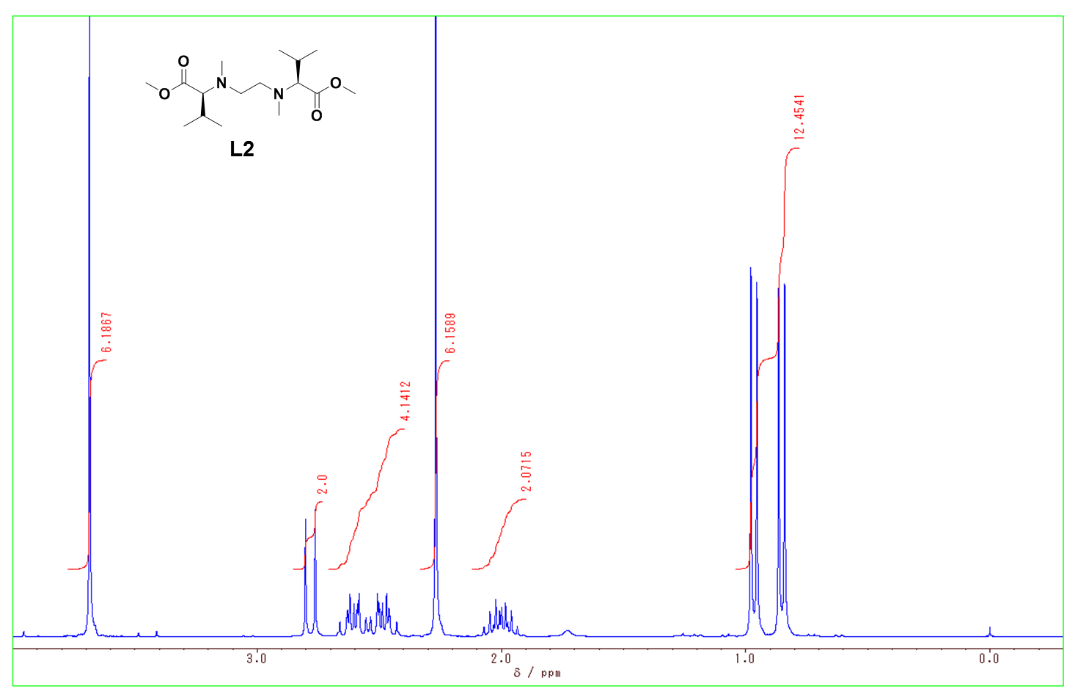


**L5**

**Figure S6.** ^1^H NMR (270 MHz, CDCl_3_, 293 K) spectrum of **L5**


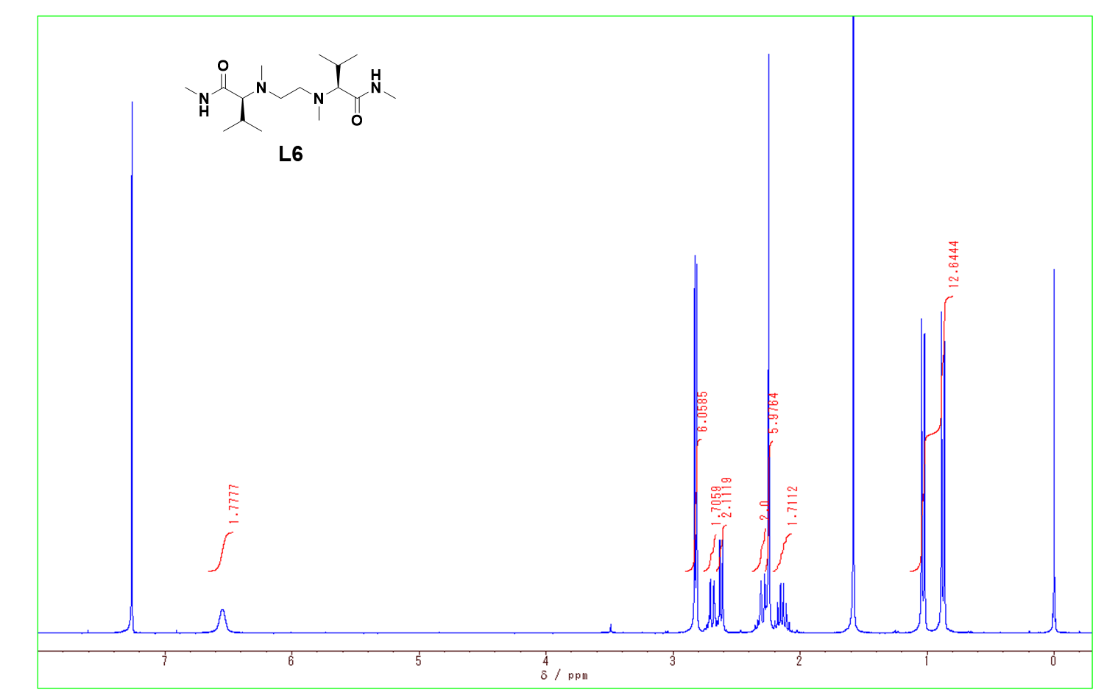


**L6**

**Figure S7.** ^1^H NMR (300 MHz, CDCl_3_, 293 K) spectrum of **L6**


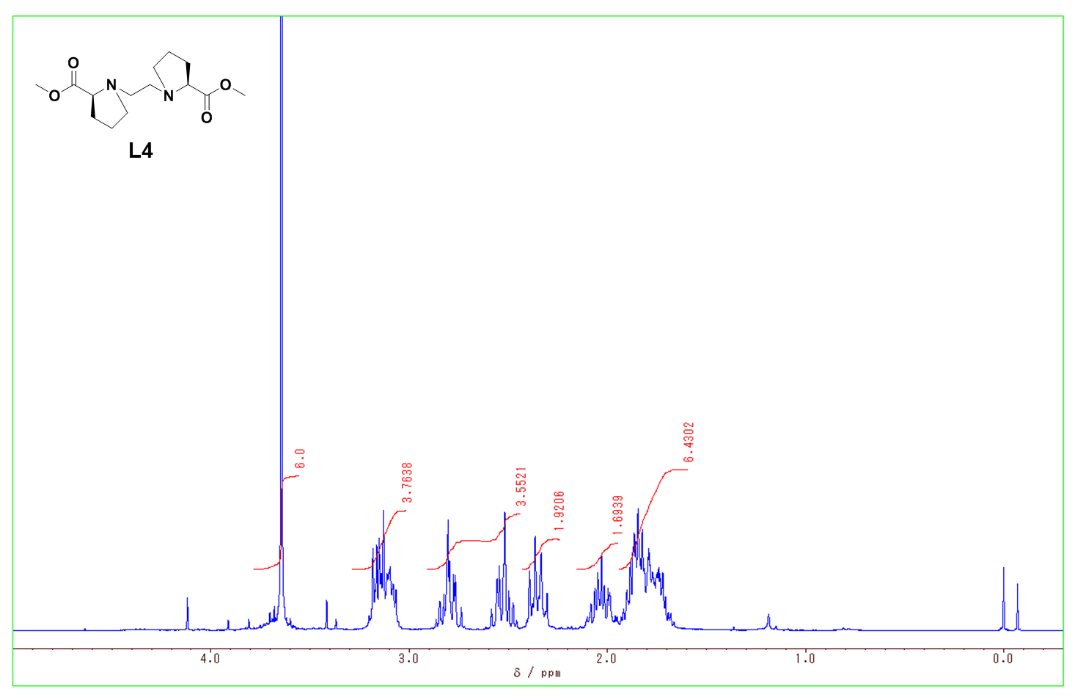


**L7**

**Figure S8.** ^1^H NMR (300 MHz, CDCl_3_, 293 K) spectrum of **L7**


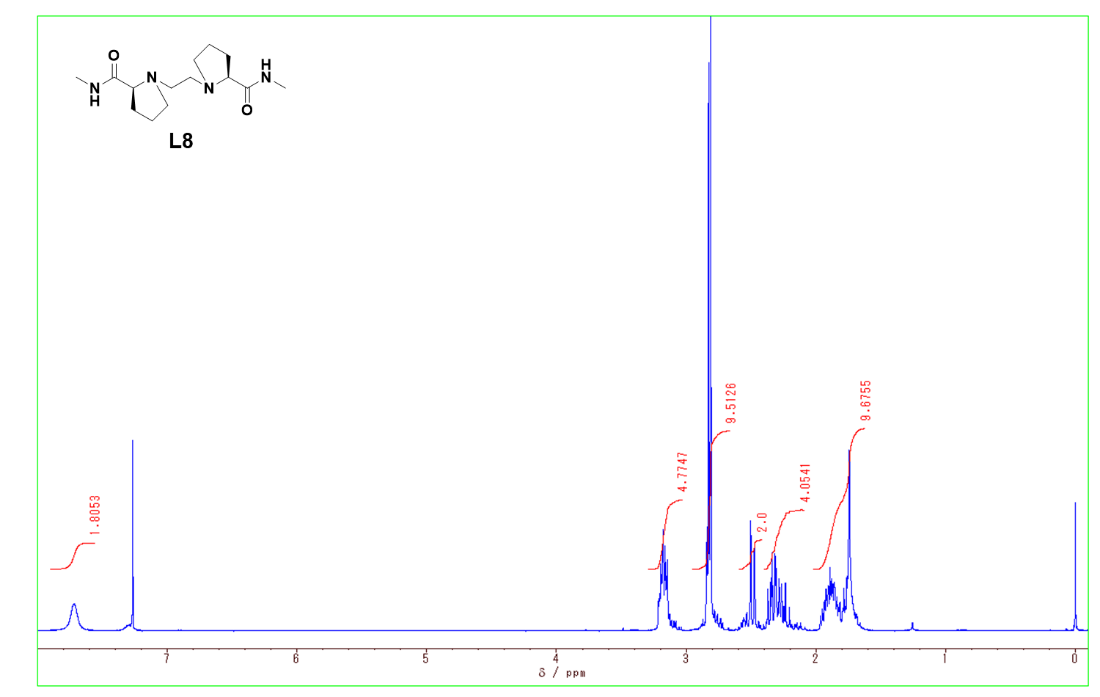


**L8**

**Figure S9.** ^1^H NMR (300 MHz, CDCl_3_, 293 K) spectrum of **L8**

# High resolution mass spectra of compounds


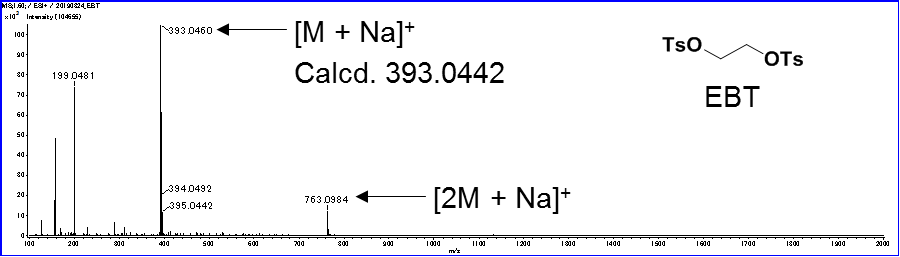


**Figure S10.** High resolution mass spectrum of ethylene bistsylate


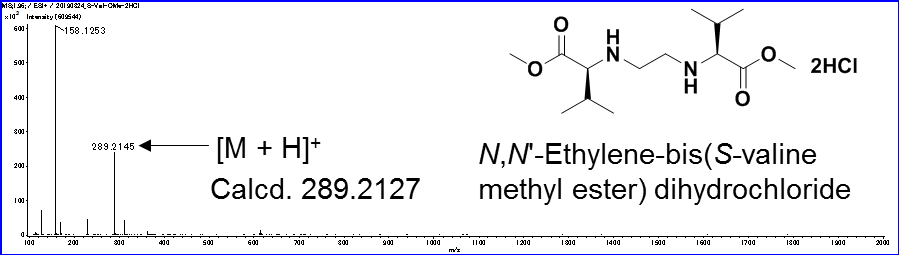


**Figure S11.** High resolution mass spectrum of *N,N´-*ethylene-bis(*S*-valine methyl ester) dihydrochloride


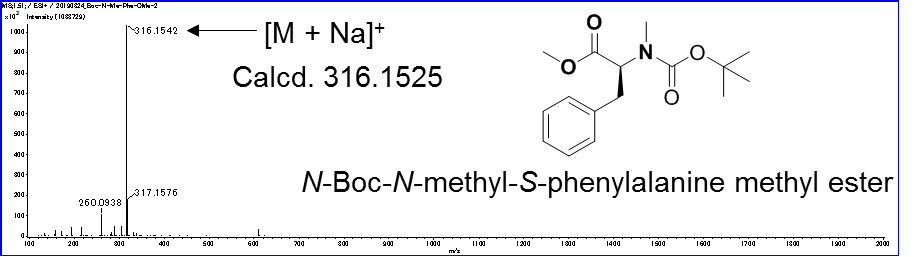


**Figure S12.** High resolution mass spectrum of *N*-Boc-*N*-methyl-*S*-phenylalanine methyl ester


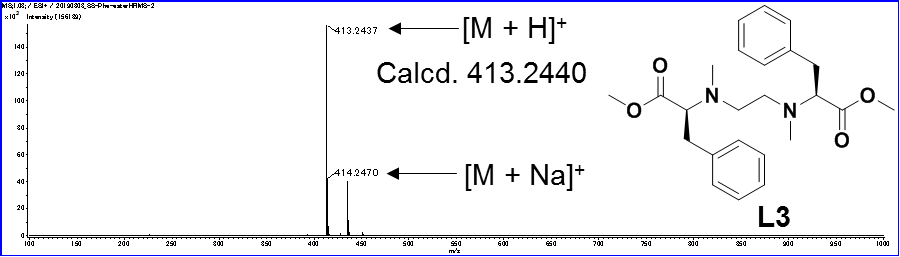


**L3**

**Figure S13.** High resolution mass spectrum of **L3**


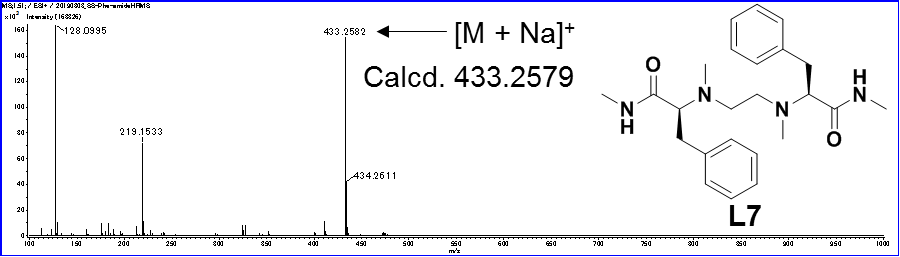


**L4**

**Figure S14.** High resolution mass spectrum of **L4**


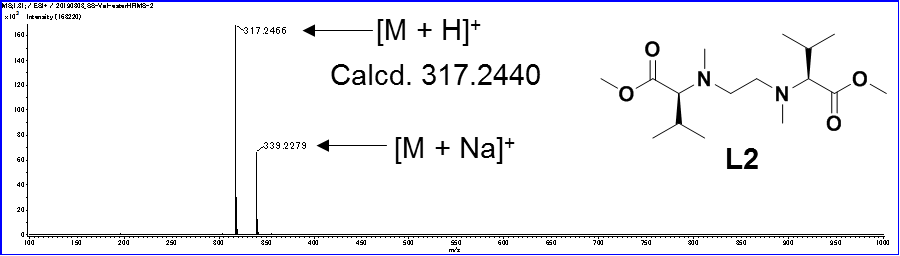


**L5**

**Figure S15.** High resolution mass spectrum of **L5**


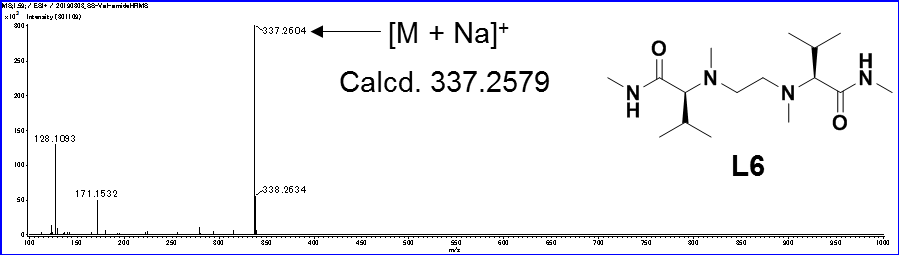


**L6**

**Figure S16.** High resolution mass spectrum of **L6**


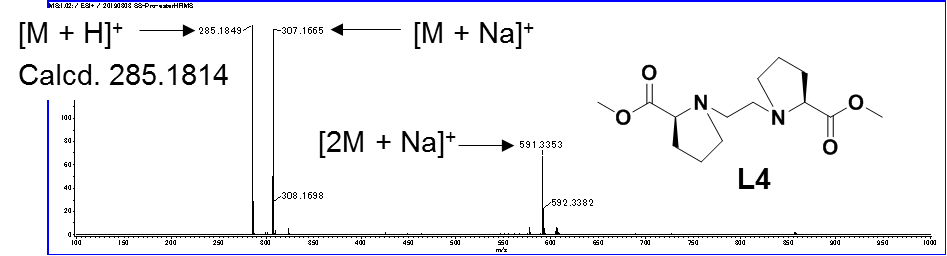


**L7**

**Figure S17.** High resolution mass spectrum of **L7**


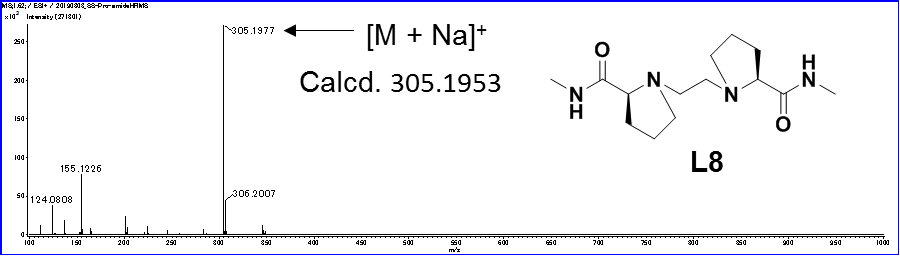


**L8**

**Figure S18.** High resolution mass spectrum of **L8**

# ESI mass spectra of the MS/EL method of CuCl_2_/L3/*R*-Val/*S*-Val-*d_8_* and CuCl_2_/L8/ *R*-Val/*S*-Val-*d_8_* in water/methanol


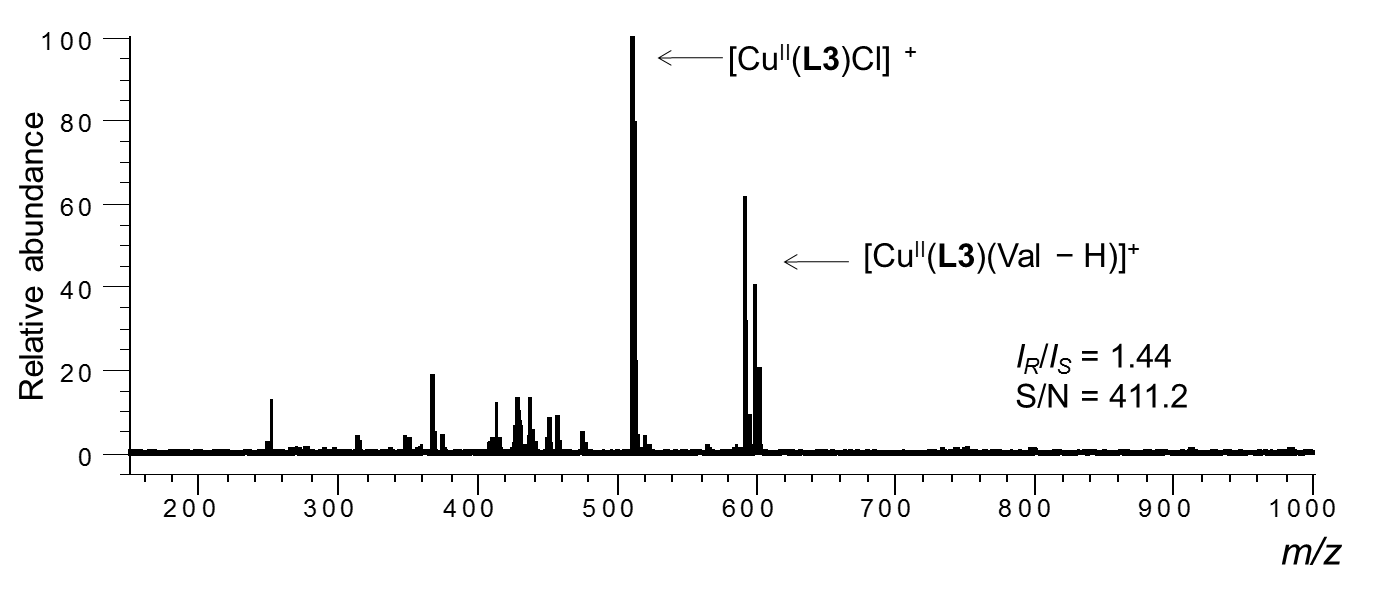


**Figure S19.** ESI mass spectra of a mixed solution of CuCl_2_/**L3**/*R*-Val/*S*-Val-*d*_8_ in water/methanol. The sample solution was prepared by mixing 1.00 mL of *in situ* prepared complex solution in methanol ([CuCl_2_]_0_ = 1.2 × 10^−4^ M and [**L3**]_0_ = 1.0 × 10^−4^ M) and a solution of an equimolar mixture of *R*-Val and *S*-Val-*d*_8_ ([*R*-Val]_0_ = *S*-Val-*d*_8_]_0_ = 5.0 × 10^−4^ M and [K_2_CO_3_]_0_ = 1.0 × 10^−3^ M) in water. The resulting mole ratio of each component [CuCl_2_]_0_/[**L3**]_0_/[*R*-Val]_0_/[*S*-Val-*d*_8_]_0_ (the amount of adding aqueous solution) = 1.2/1.0/0.25/0.25 (50 μL).


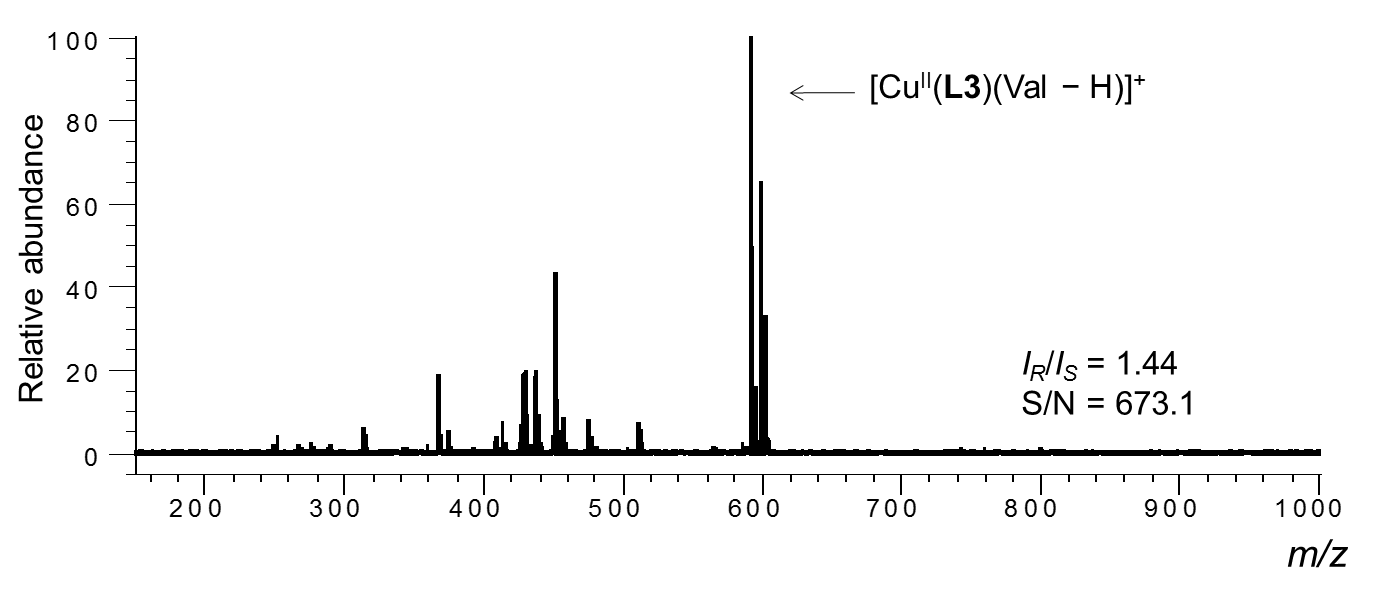


**Figure S20.** ESI mass spectra of a mixed solution of CuCl_2_/**L3**/*R*-Val/*S*-Val-*d*_8_ in water/methanol. The sample solution was prepared by mixing 1.00 mL of *in situ* prepared complex solution in methanol ([CuCl_2_]_0_ = 1.2 × 10^−4^ M and [**L3**]_0_ = 1.0 × 10^−4^ M) and a solution of an equimolar mixture of *R*-Val and *S*-Val-*d*_8_ ([*R*-Val]_0_ = *S*-Val-*d*_8_]_0_ = 5.0 × 10^−4^ M and [K_2_CO_3_]_0_ = 1.0 × 10^−3^ M) in water. The resulting mole ratio of each component [CuCl_2_]_0_/[**L3**]_0_/[*R*-Val]_0_/[*S*-Val-*d*_8_]_0_ (the amount of adding aqueous solution) = 1.2/1.0/0.5/0.5 (100 μL).


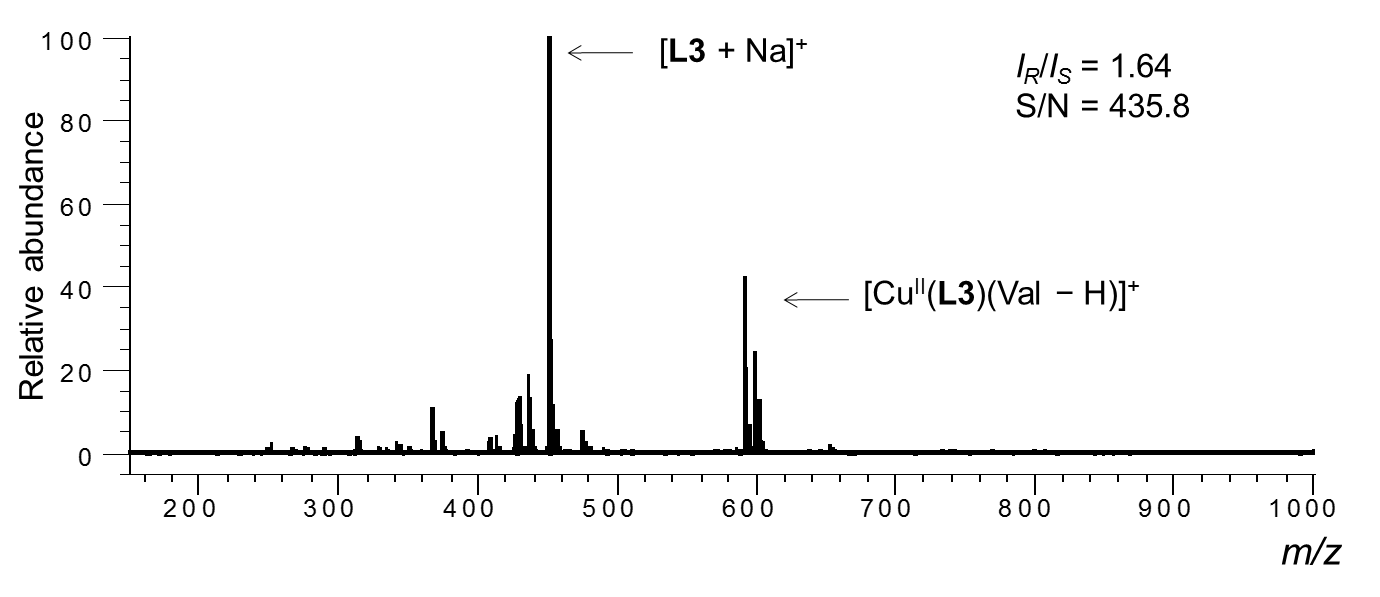


**Figure S21.** ESI mass spectra of a mixed solution of CuCl_2_/**L3**/*R*-Val/*S*-Val-*d*_8_ in water/methanol. The sample solution was prepared by mixing 1.00 mL of *in situ* prepared complex solution in methanol ([CuCl_2_]_0_ = 1.2 × 10^−4^ M and [**L3**]_0_ = 1.0 × 10^−4^ M) and a solution of an equimolar mixture of *R*-Val and *S*-Val-*d*_8_ ([*R*-Val]_0_ = *S*-Val-*d*_8_]_0_ = 5.0 × 10^−4^ M and [K_2_CO_3_]_0_ = 1.0 × 10^−3^ M) in water. The resulting mole ratio of each component [CuCl_2_]_0_/[**L3**]_0_/[*R*-Val]_0_/[*S*-Val-*d*_8_]_0_ (the amount of adding aqueous solution) = 1.2/1.0/0.75/0.75 (150 μL).


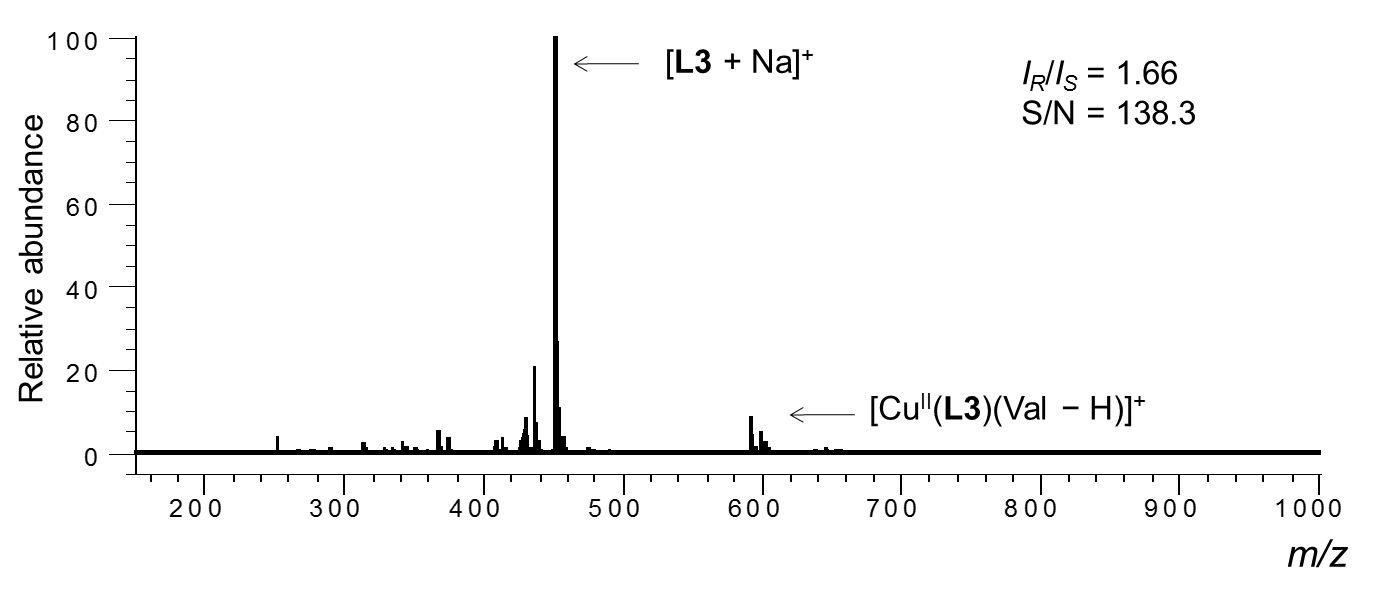


**Figure S22.** ESI mass spectra of a mixed solution of CuCl_2_/**L3**/*R*-Val/*S*-Val-*d*_8_ in water/methanol. The sample solution was prepared by mixing 1.00 mL of *in situ* prepared complex solution in methanol ([CuCl_2_]_0_ = 1.2 × 10^−4^ M and [**L3**]_0_ = 1.0 × 10^−4^ M) and a solution of an equimolar mixture of *R*-Val and *S*-Val-*d*_8_ ([*R*-Val]_0_ = *S*-Val-*d*_8_]_0_ = 5.0 × 10^−4^ M and [K_2_CO_3_]_0_ = 1.0 × 10^−3^ M) in water. The resulting mole ratio of each component [CuCl_2_]_0_/[**L3**]_0_/[*R*-Val]_0_/[*S*-Val-*d*_8_]_0_ (the amount of adding aqueous solution) = 1.2/1.0/1.0/1.0 (200 μL).


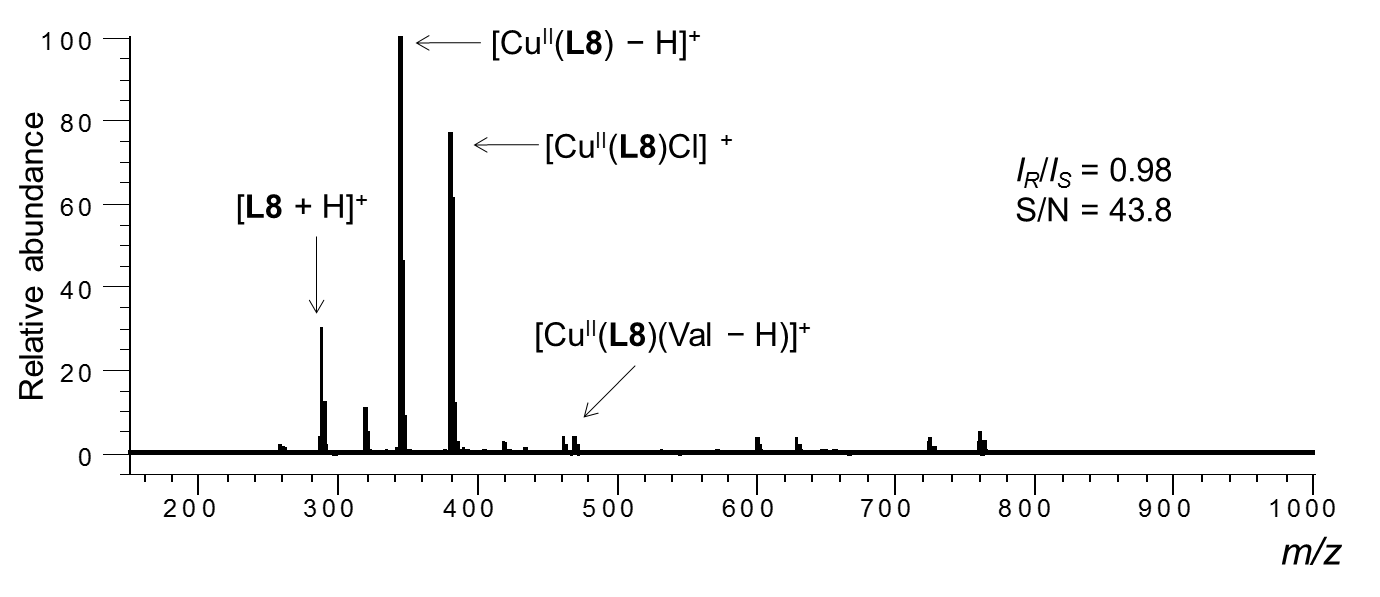


**Figure S23.** ESI mass spectra of a mixed solution of CuCl_2_/**L8**/*R*-Val/*S*-Val-*d*_8_ in water/methanol. The sample solution was prepared by mixing 1.00 mL of *in situ* prepared complex solution in methanol ([CuCl_2_]_0_ = 1.2 × 10^−4^ M and [**L8**]_0_ = 1.0 × 10^−4^ M) and a solution of an equimolar mixture of *R*-Val and *S*-Val-*d*_8_ ([*R*-Val]_0_ = *S*-Val-*d*_8_]_0_ = 5.0 × 10^−4^ M and [K_2_CO_3_]_0_ = 1.0 × 10^−3^ M) in water. The resulting mole ratio of each component [CuCl_2_]_0_/[**L8**]_0_/[*R*-Val]_0_/[*S*-Val-*d*_8_]_0_ (the amount of adding aqueous solution) = 1.2/1.0/0.25/0.25 (50 μL).


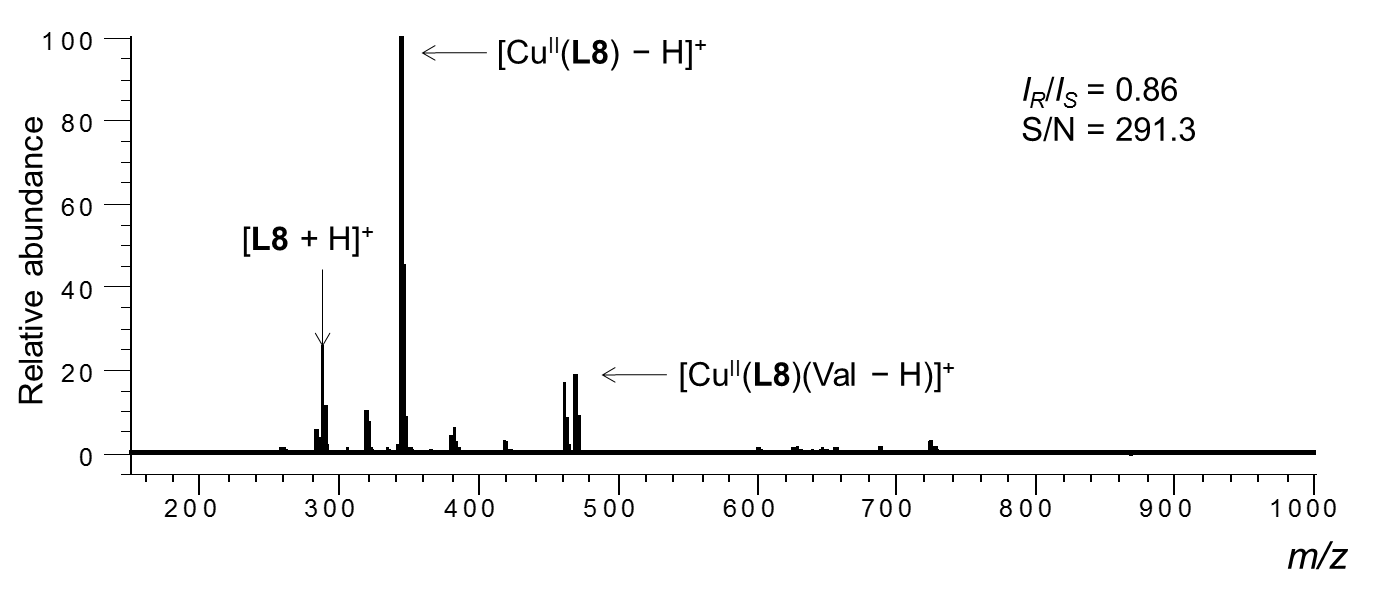


**Figure S24.** ESI mass spectra of a mixed solution of CuCl_2_/**L8**/*R*-Val/*S*-Val-*d*_8_ in water/methanol. The sample solution was prepared by mixing 1.00 mL of *in situ* prepared complex solution in methanol ([CuCl_2_]_0_ = 1.2 × 10^−4^ M and [**L8**]_0_ = 1.0 × 10^−4^ M) and a solution of an equimolar mixture of *R*-Val and *S*-Val-*d*_8_ ([*R*-Val]_0_ = *S*-Val-*d*_8_]_0_ = 5.0 × 10^−4^ M and [K_2_CO_3_]_0_ = 1.0 × 10^−3^ M) in water. The resulting mole ratio of each component [CuCl_2_]_0_/[**L8**]_0_/[*R*-Val]_0_/[*S*-Val-*d*_8_]_0_ (the amount of adding aqueous solution) = 1.2/1.0/0.5/0.5 (100 μL).


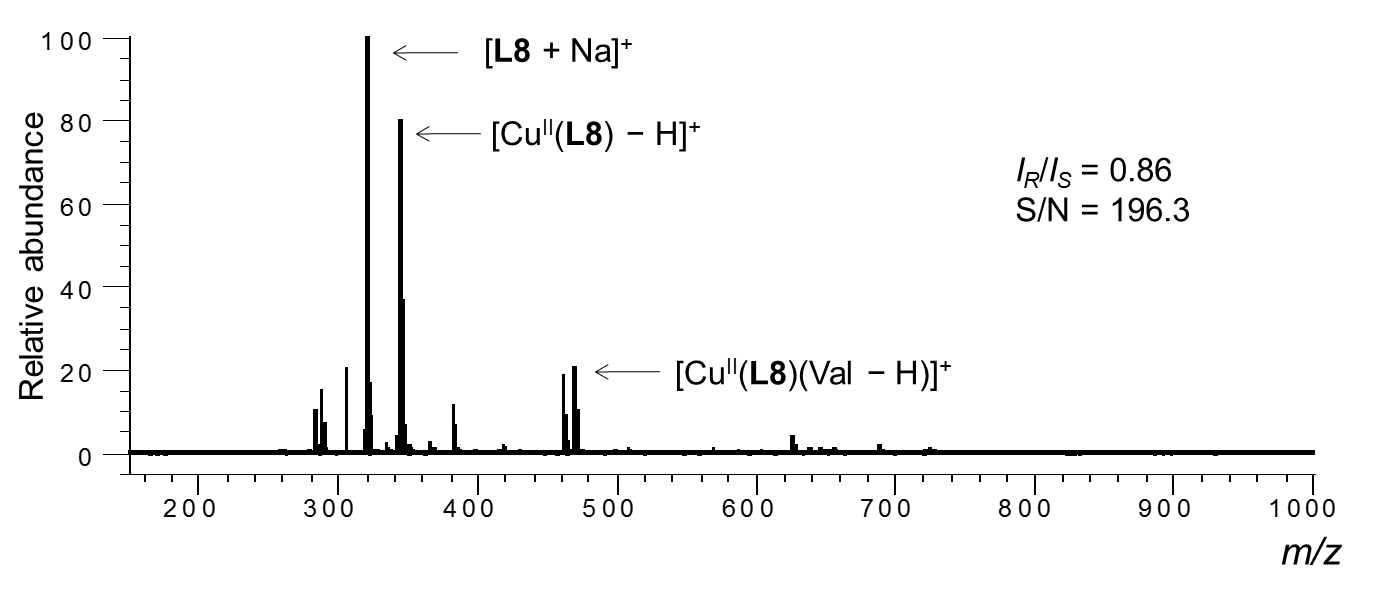


**Figure S25.** ESI mass spectra of a mixed solution of CuCl_2_/**L8**/*R*-Val/*S*-Val-*d*_8_ in water/methanol. The sample solution was prepared by mixing 1.00 mL of *in situ* prepared complex solution in methanol ([CuCl_2_]_0_ = 1.2 × 10^−4^ M and [**L8**]_0_ = 1.0 × 10^−4^ M) and a solution of an equimolar mixture of *R*-Val and *S*-Val-*d*_8_ ([*R*-Val]_0_ = *S*-Val-*d*_8_]_0_ = 5.0 × 10^−4^ M and [K_2_CO_3_]_0_ = 1.0 × 10^−3^ M) in water. The resulting mole ratio of each component [CuCl_2_]_0_/[**L8**]_0_/[*R*-Val]_0_/[*S*-Val-*d*_8_]_0_ (the amount of adding aqueous solution) = 1.2/1.0/0.75/0.75 (150 μL).


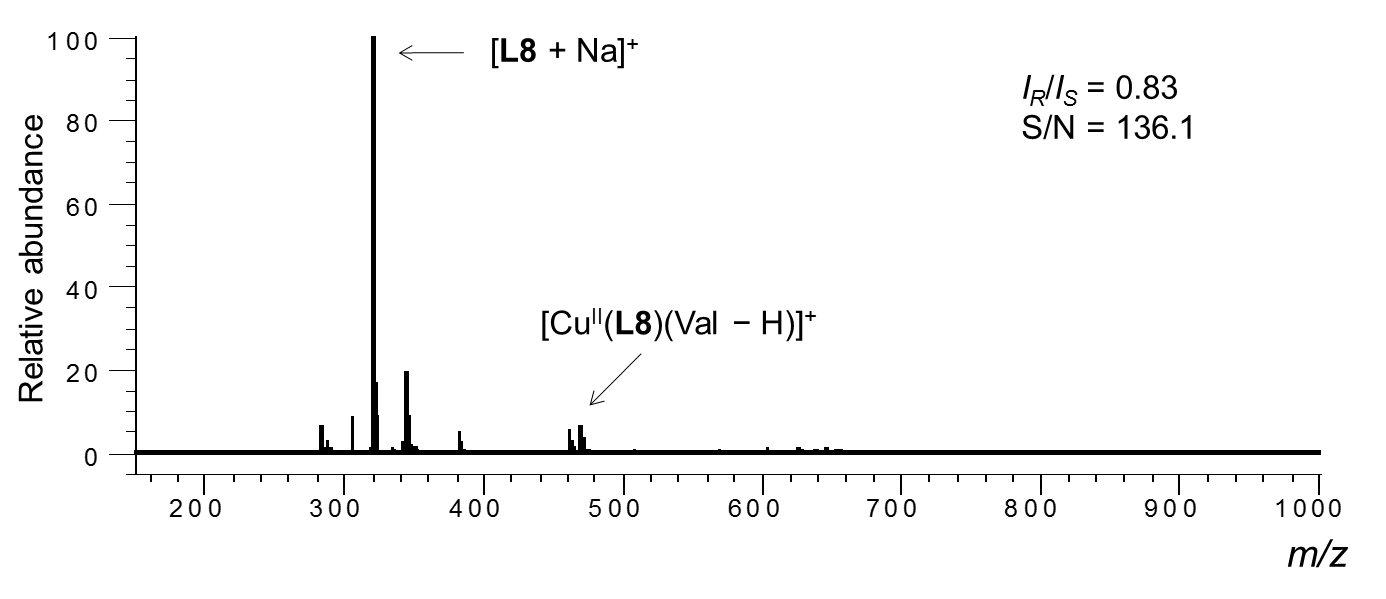


**Figure S26.** ESI mass spectra of a mixed solution of CuCl_2_/**L8**/*R*-Val/*S*-Val-*d*_8_ in water/methanol. The sample solution was prepared by mixing 1.00 mL of *in situ* prepared complex solution in methanol ([CuCl_2_]_0_ = 1.2 × 10^−4^ M and [**L8**]_0_ = 1.0 × 10^−4^ M) and a solution of an equimolar mixture of *R*-Val and *S*-Val-*d*_8_ ([*R*-Val]_0_ = *S*-Val-*d*_8_]_0_ = 5.0 × 10^−4^ M and [K_2_CO_3_]_0_ = 1.0 × 10^−3^ M) in water. The resulting mole ratio of each component [CuCl_2_]_0_/[**L8**]_0_/[*R*-Val]_0_/[*S*-Val-*d*_8_]_0_ (the amount of adding aqueous solution) = 1.2/1.0/1.0/1.0 (200 μL).

# ESI mass spectra of the MS/EL method of CuCl_2_/L/*R*-AA/S-AA-*d_n_* in water/ methanol


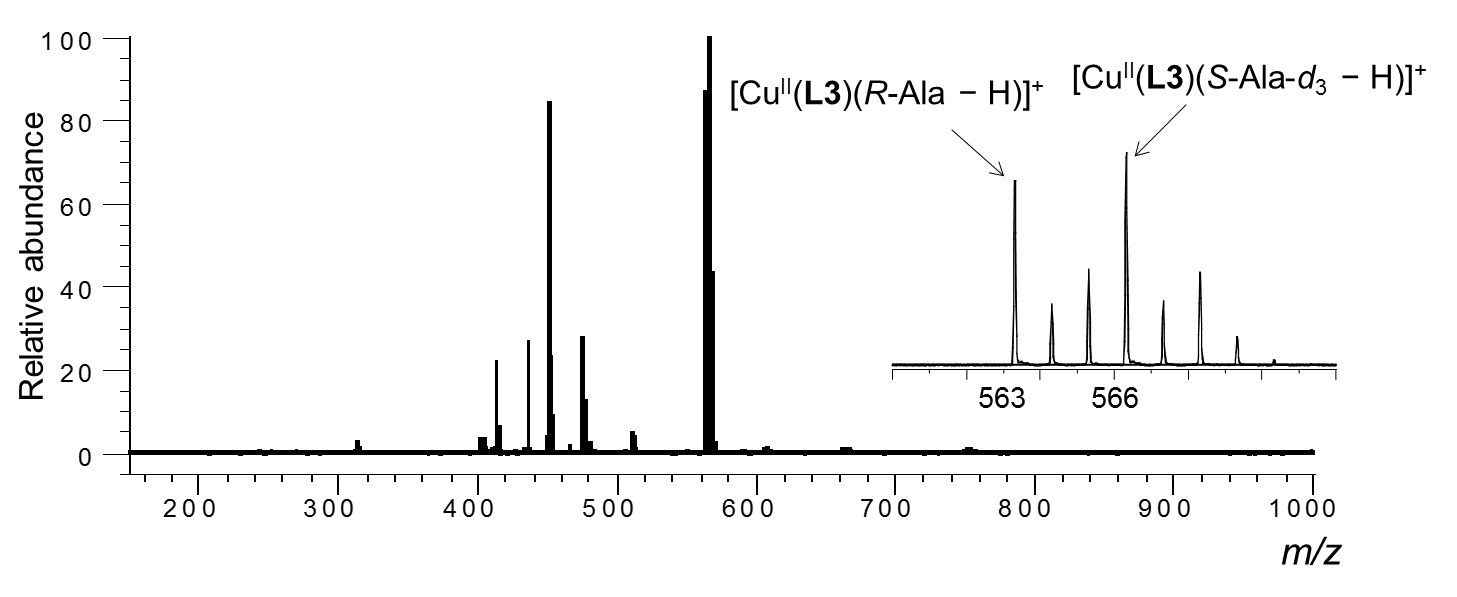


**Figure S27.** ESI mass spectra of the MS/EL method of CuCl_2_/**L**/*R*-AA/*S*-AA-*d*_n_- in water/methanol (1/10, v/v). [CuCl_2_]_0_ = 1.09 × 10^−4^ M, [**L**]_0_ = 9.09 × 10^−5^ M and [*R*-AA]_0_ = [*S*-AA-*d*_n_]_0_ = 4.55 × 10^−5^ M. [CuCl_2_]_0_/[**L**]_0_/[*R*-AA]_0_/[*S*-AA-*d*_n_]_0_ = 1.2/1.0/0.5/0.5. [K_2_CO_3_]_0_ = 9.09 × 10^−5^ M, **L** = **L3**, AA= *R*-Ala/*S*-Ala-*d*_3_.


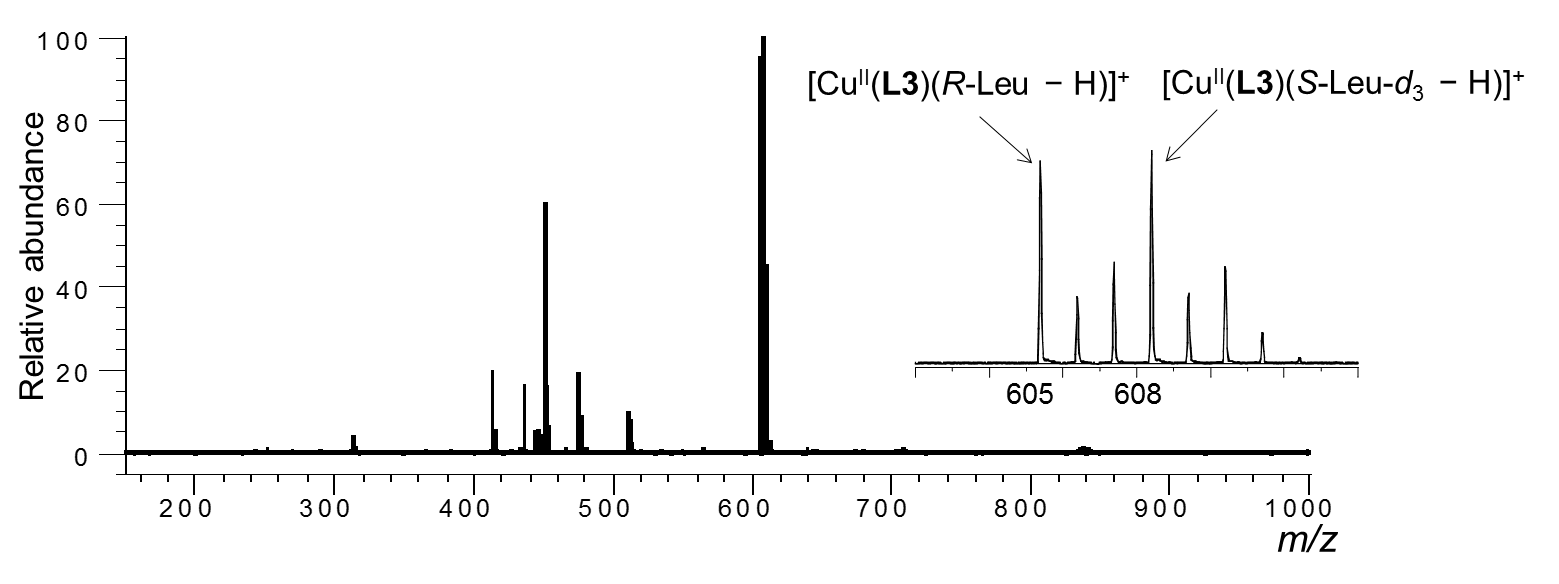


**Figure S28.** ESI mass spectra of the MS/EL method of CuCl_2_/**L**/*R*-AA/*S*-AA-*d*_n_- in water/methanol (1/10, v/v). [CuCl_2_]_0_ = 1.09 × 10^−4^ M, [**L**]_0_ = 9.09 × 10^−5^ M and [*R*-AA]_0_ = [*S*-AA-*d*_n_]_0_ = 4.55 × 10^−5^ M. [CuCl_2_]_0_/[**L**]_0_/[*R*-AA]_0_/[*S*-AA-*d*_n_]_0_ = 1.2/1.0/0.5/0.5. [K_2_CO_3_]_0_ = 9.09 × 10^−5^ M, **L** = **L3**, AA= *R*-Leu/*S*-Leu-*d*_3_.


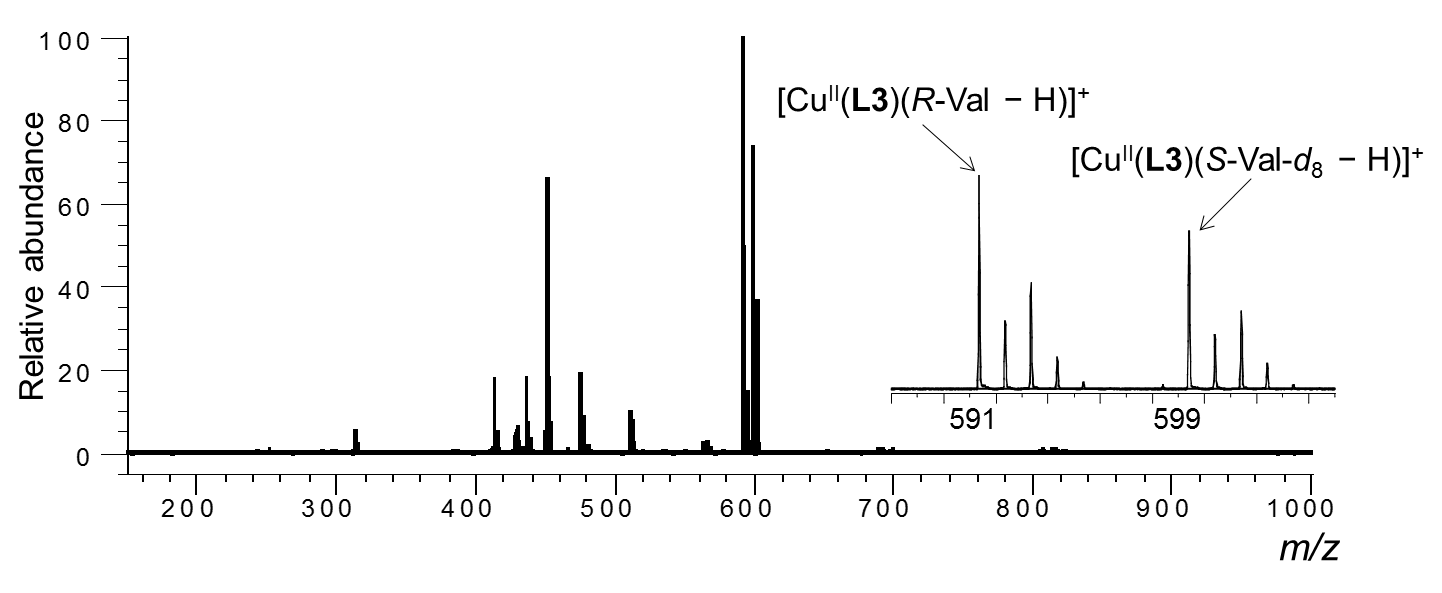


**Figure S29.** ESI mass spectra of the MS/EL method of CuCl_2_/**L**/*R*-AA/*S*-AA-*d*_n_- in water/methanol (1/10, v/v). [CuCl_2_]_0_ = 1.09 × 10^−4^ M, [**L**]_0_ = 9.09 × 10^−5^ M and [*R*-AA]_0_ = [*S*-AA-*d*_n_]_0_ = 4.55 × 10^−5^ M. [CuCl_2_]_0_/[**L**]_0_/[*R*-AA]_0_/[*S*-AA-*d*_n_]_0_ = 1.2/1.0/0.5/0.5. [K_2_CO_3_]_0_ = 9.09 × 10^−5^ M, **L** = **L3**, AA= *R*-Val/*S*-Val-*d*_8_.


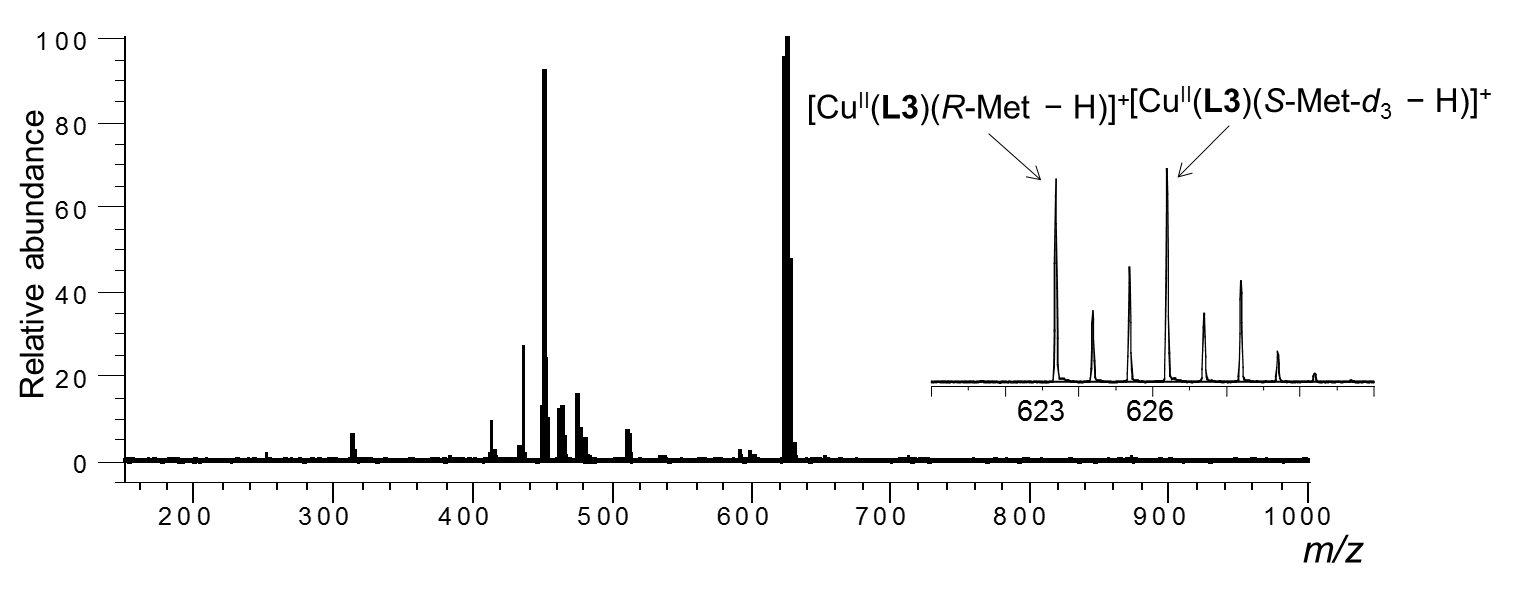


**Figure S30.** ESI mass spectra of the MS/EL method of CuCl_2_/**L**/*R*-AA/*S*-AA-*d*_n_- in water/methanol (1/10, v/v). [CuCl_2_]_0_ = 1.09 × 10^−4^ M, [**L**]_0_ = 9.09 × 10^−5^ M and [*R*-AA]_0_ = [*S*-AA-*d*_n_]_0_ = 4.55 × 10^−5^ M. [CuCl_2_]_0_/[**L**]_0_/[*R*-AA]_0_/[*S*-AA-*d*_n_]_0_ = 1.2/1.0/0.5/0.5. [K_2_CO_3_]_0_ = 9.09 × 10^−5^ M, **L** = **L3**, AA= *R*-Met/*S*-Met-*d*_3_.


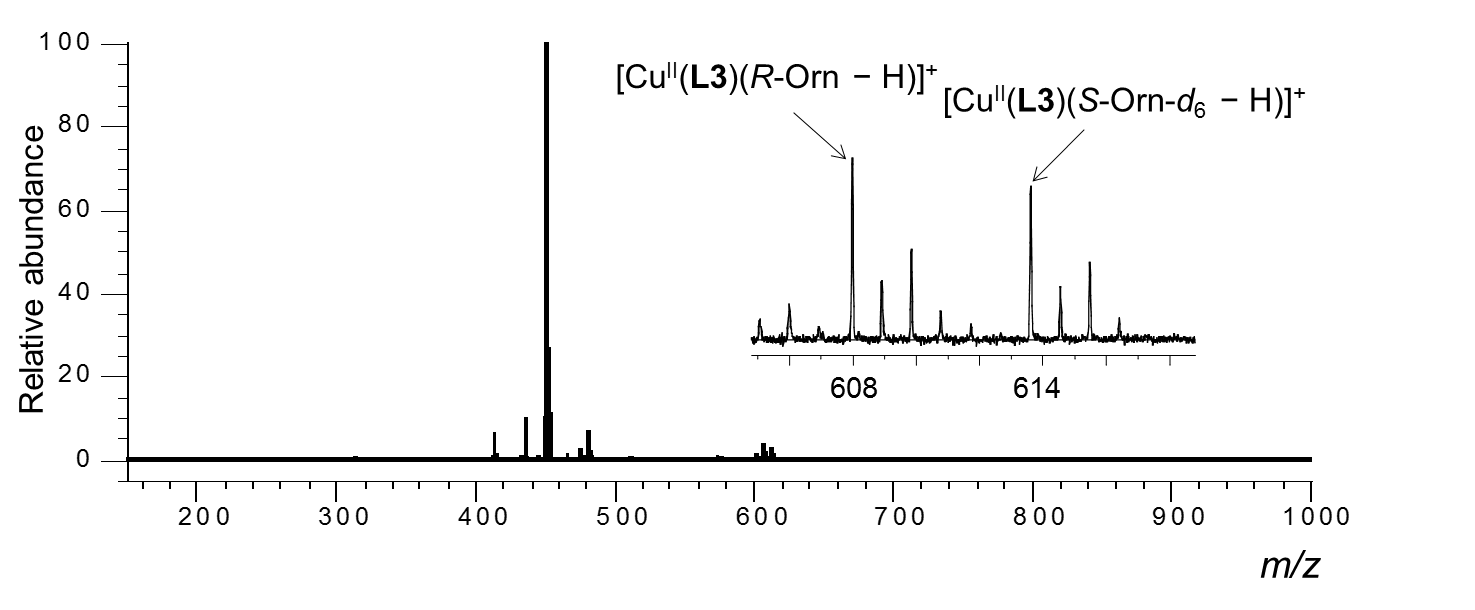


**Figure S31.** ESI mass spectra of the MS/EL method of CuCl_2_/**L**/*R*-AA/*S*-AA-*d*_n_- in water/methanol (1/10, v/v). [CuCl_2_]_0_ = 1.09 × 10^−4^ M, [**L**]_0_ = 9.09 × 10^−5^ M and [*R*-AA]_0_ = [*S*-AA-*d*_n_]_0_ = 4.55 × 10^−5^ M. [CuCl_2_]_0_/[**L**]_0_/[*R*-AA]_0_/[*S*-AA-*d*_n_]_0_ = 1.2/1.0/0.5/0.5. [K_2_CO_3_]_0_ = 9.09 × 10^−5^ M, **L** = **L3**, AA= *R*-Orn/*S*-Orn-*d*_6_.


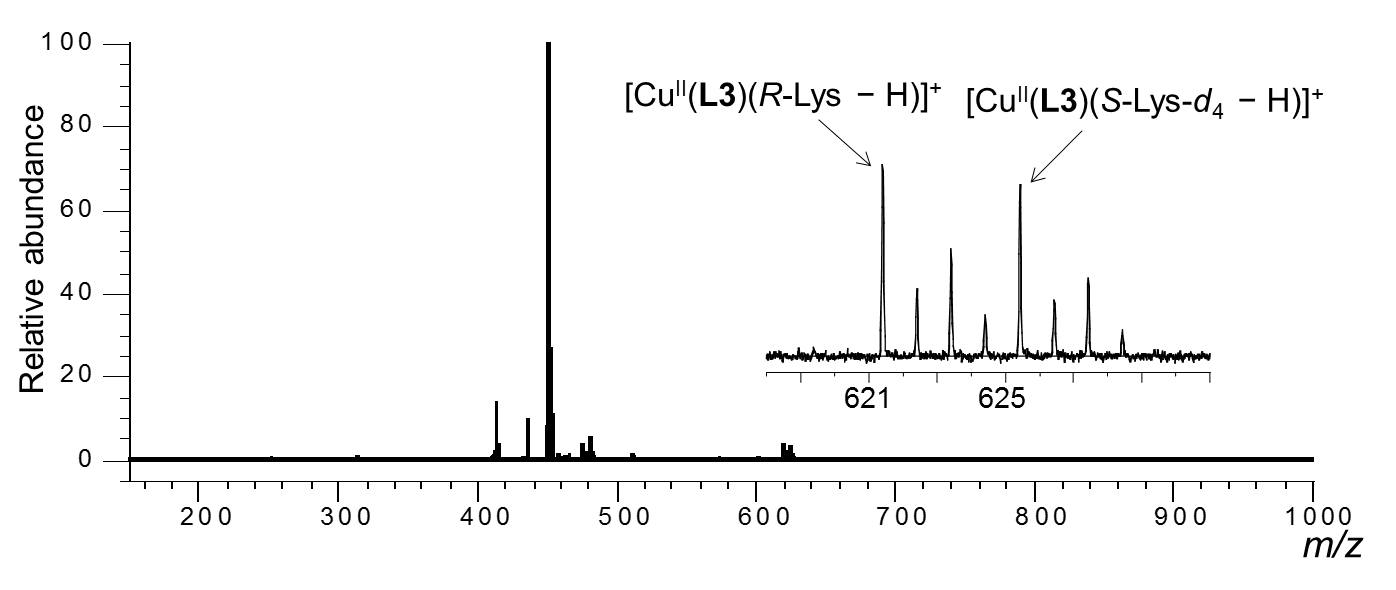


**Figure S32.** ESI mass spectra of the MS/EL method of CuCl_2_/**L**/*R*-AA/*S*-AA-*d*_n_- in water/methanol (1/10, v/v). [CuCl_2_]_0_ = 1.09 × 10^−4^ M, [**L**]_0_ = 9.09 × 10^−5^ M and [*R*-AA]_0_ = [*S*-AA-*d*_n_]_0_ = 4.55 × 10^−5^ M. [CuCl_2_]_0_/[**L**]_0_/[*R*-AA]_0_/[*S*-AA-*d*_n_]_0_ = 1.2/1.0/0.5/0.5. [K_2_CO_3_]_0_ = 9.09 × 10^−5^ M, **L** = **L3**, AA= *R*-Lys/*S*-Lys-*d*_4_.


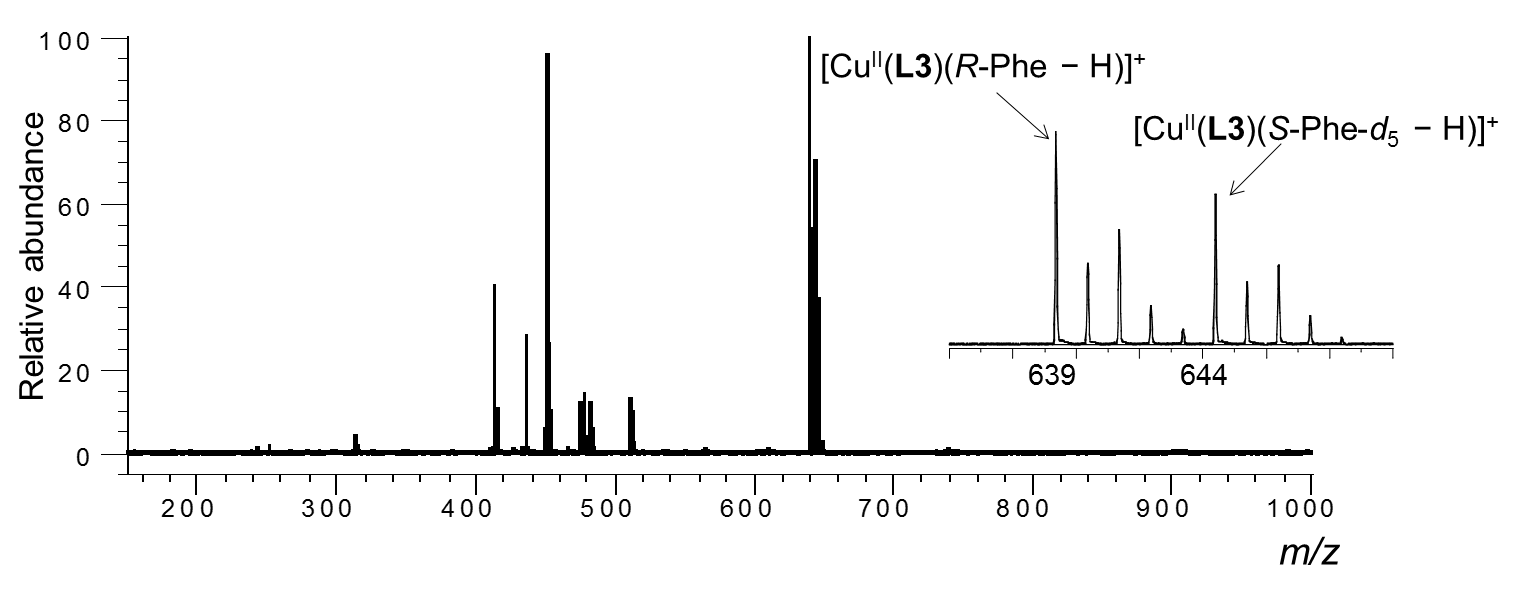


**Figure S33.** ESI mass spectra of the MS/EL method of CuCl_2_/**L**/*R*-AA/*S*-AA-*d*_n_- in water/methanol (1/10, v/v). [CuCl_2_]_0_ = 1.09 × 10^−4^ M, [**L**]_0_ = 9.09 × 10^−5^ M and [*R*-AA]_0_ = [*S*-AA-*d*_n_]_0_ = 4.55 × 10^−5^ M. [CuCl_2_]_0_/[**L**]_0_/[*R*-AA]_0_/[*S*-AA-*d*_n_]_0_ = 1.2/1.0/0.5/0.5. [K_2_CO_3_]_0_ = 9.09 × 10^−5^ M, **L** = **L3**, AA= *R*-Phe/*S*-Phe-*d*_5_.


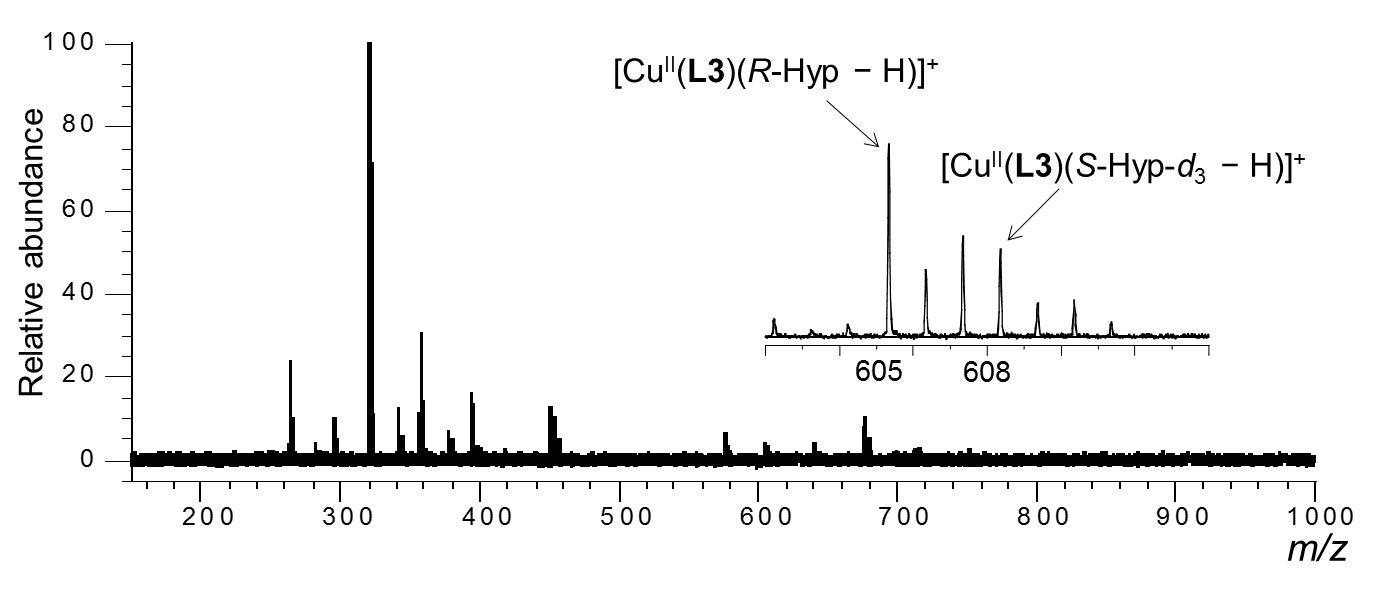


**Figure S34.** ESI mass spectra of the MS/EL method of CuCl_2_/**L**/*R*-AA/*S*-AA-*d*_n_- in water/methanol (1/10, v/v). [CuCl_2_]_0_ = 1.09 × 10^−4^ M, [**L**]_0_ = 9.09 × 10^−5^ M and [*R*-AA]_0_ = [*S*-AA-*d*_n_]_0_ = 4.55 × 10^−5^ M. [CuCl_2_]_0_/[**L**]_0_/[*R*-AA]_0_/[*S*-AA-*d*_n_]_0_ = 1.2/1.0/0.5/0.5. [K_2_CO_3_]_0_ = 9.09 × 10^−5^ M, **L** = **L3**, AA= *R*-Hyp/*S*-Hyp-*d*_3_.


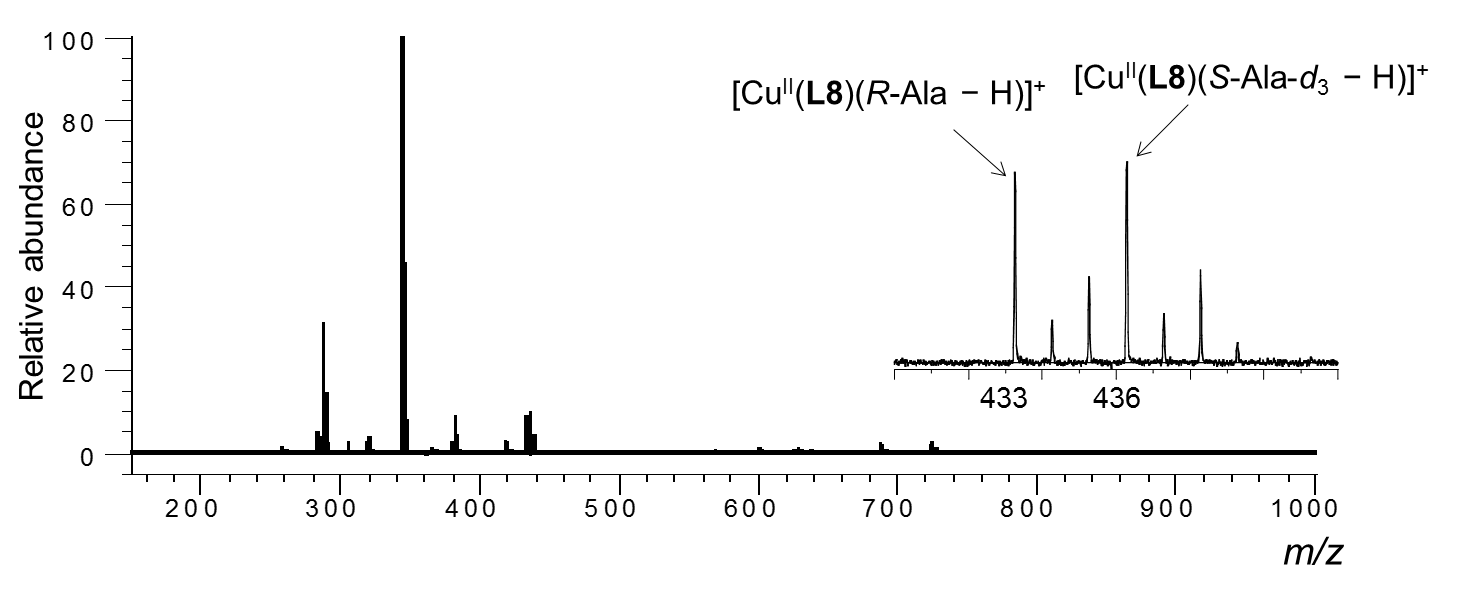


**Figure S35.** ESI mass spectra of the MS/EL method of CuCl_2_/**L**/*R*-AA/*S*-AA-*d*_n_- in water/methanol (1/10, v/v). [CuCl_2_]_0_ = 1.09 × 10^−4^ M, [**L**]_0_ = 9.09 × 10^−5^ M and [*R*-AA]_0_ = [*S*-AA-*d*_n_]_0_ = 4.55 × 10^−5^ M. [CuCl_2_]_0_/[**L**]_0_/[*R*-AA]_0_/[*S*-AA-*d*_n_]_0_ = 1.2/1.0/0.5/0.5. [K_2_CO_3_]_0_ = 9.09 × 10^−5^ M, **L** = **L8**, AA= *R*-Ala/*S*-Ala-*d*_3_.


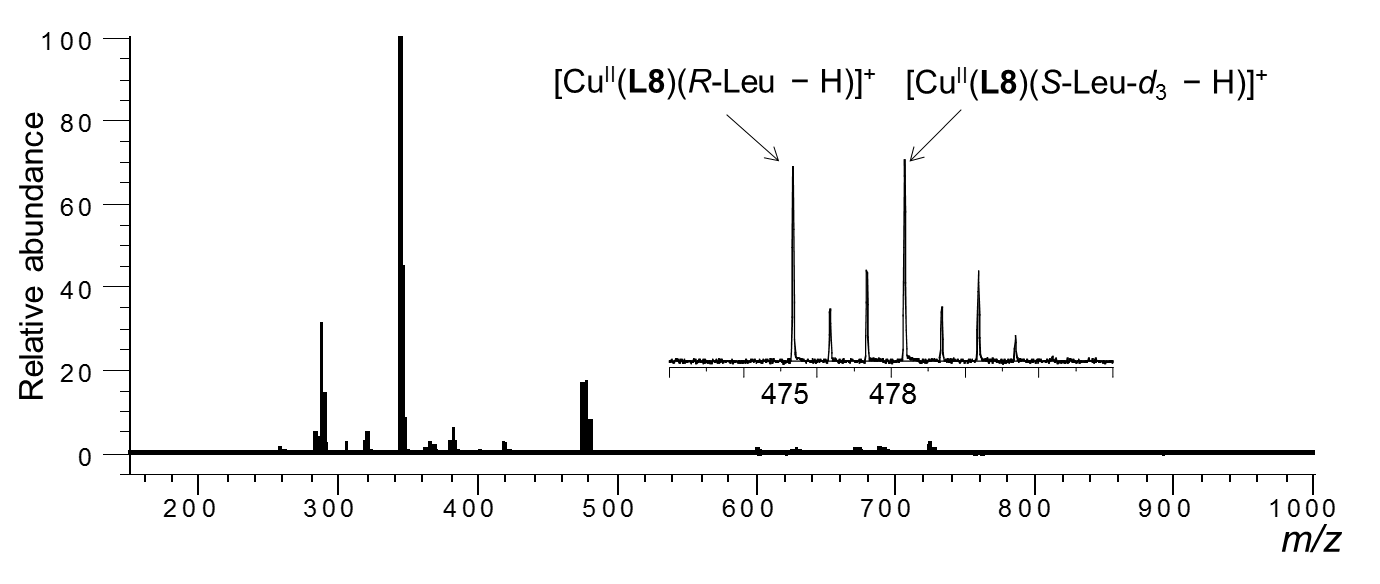


**Figure S36.** ESI mass spectra of the MS/EL method of CuCl_2_/**L**/*R*-AA/*S*-AA-*d*_n_- in water/methanol (1/10, v/v). [CuCl_2_]_0_ = 1.09 × 10^−4^ M, [**L**]_0_ = 9.09 × 10^−5^ M and [*R*-AA]_0_ = [*S*-AA-*d*_n_]_0_ = 4.55 × 10^−5^ M. [CuCl_2_]_0_/[**L**]_0_/[*R*-AA]_0_/[*S*-AA-*d*_n_]_0_ = 1.2/1.0/0.5/0.5. [K_2_CO_3_]_0_ = 9.09 × 10^−5^ M, **L** = **L8**, AA= *R*-Leu/*S*-Leu-*d*_3_.


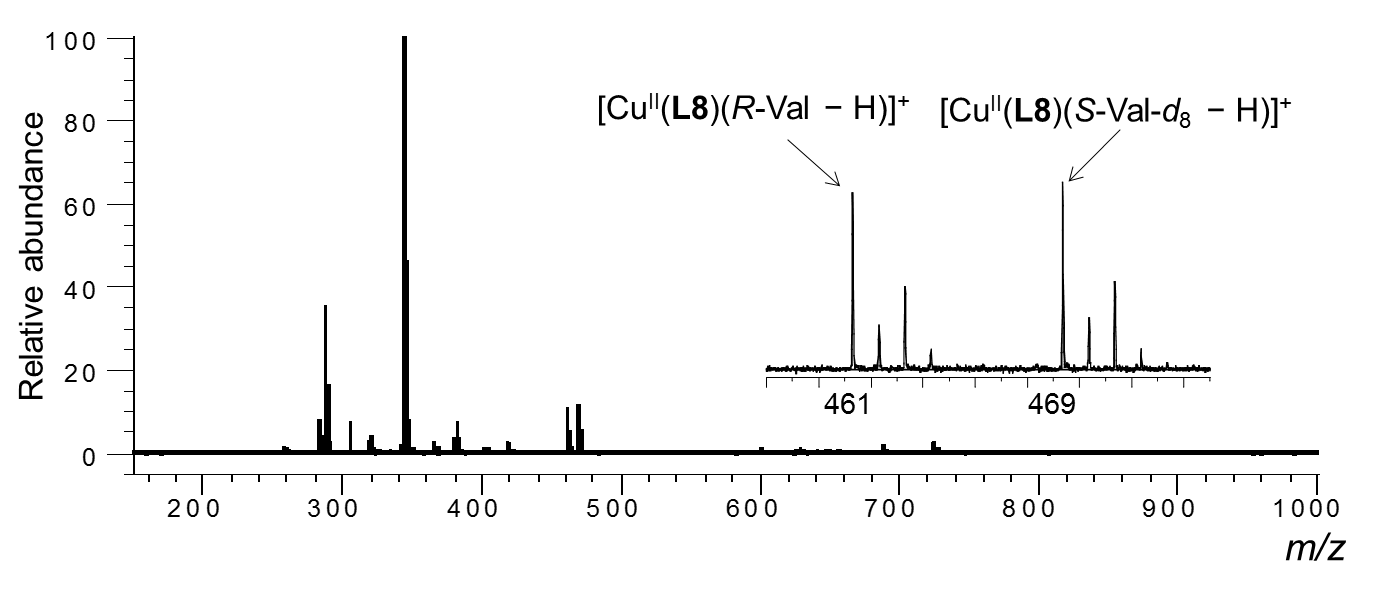


**Figure 37.** ESI mass spectra of the MS/EL method of CuCl_2_/**L**/*R*-AA/*S*-AA-*d*_n_- in water/methanol (1/10, v/v). [CuCl_2_]_0_ = 1.09 × 10^−4^ M, [**L**]_0_ = 9.09 × 10^−5^ M and [*R*-AA]_0_ = [*S*-AA-*d*_n_]_0_ = 4.55 × 10^−5^ M. [CuCl_2_]_0_/[**L**]_0_/[*R*-AA]_0_/[*S*-AA-*d*_n_]_0_ = 1.2/1.0/0.5/0.5. [K_2_CO_3_]_0_ = 9.09 × 10^−5^ M, **L** = **L8**, AA= *R*-Val/*S*-Val-*d*_8_.


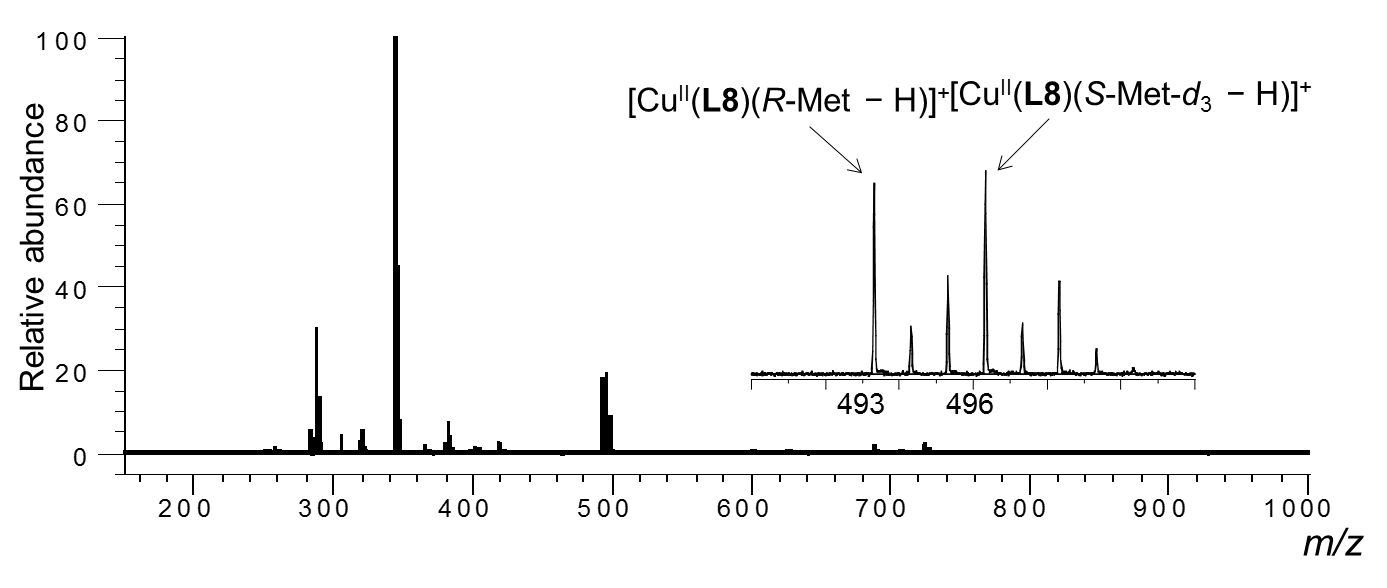


**Figure S38.** ESI mass spectra of the MS/EL method of CuCl_2_/**L**/*R*-AA/*S*-AA-*d*_n_- in water/methanol (1/10, v/v). [CuCl_2_]_0_ = 1.09 × 10^−4^ M, [**L**]_0_ = 9.09 × 10^−5^ M and [*R*-AA]_0_ = [*S*-AA-*d*_n_]_0_ = 4.55 × 10^−5^ M. [CuCl_2_]_0_/[**L**]_0_/[*R*-AA]_0_/[*S*-AA-*d*_n_]_0_ = 1.2/1.0/0.5/0.5. [K_2_CO_3_]_0_ = 9.09 × 10^−5^ M, **L** = **L8**, AA= *R*-Met/*S*-Met-*d*_3_.


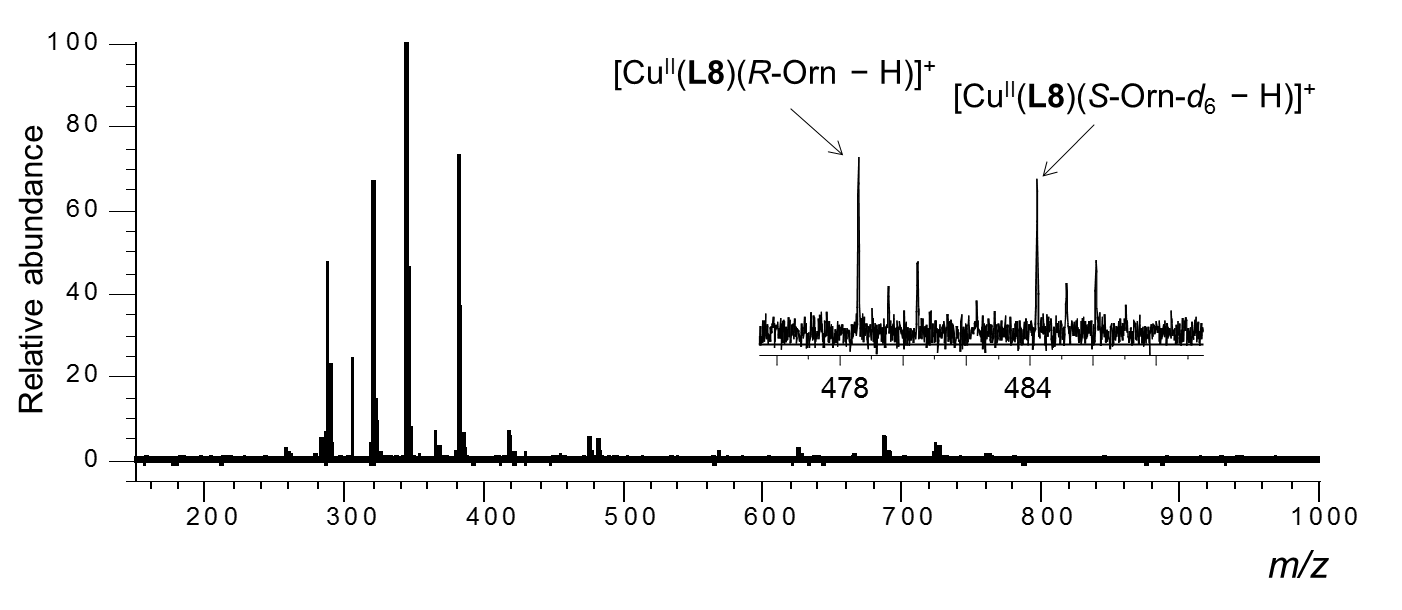


**Figure S39.** ESI mass spectra of the MS/EL method of CuCl_2_/**L**/*R*-AA/*S*-AA-*d*_n_- in water/methanol (1/10, v/v). [CuCl_2_]_0_ = 1.09 × 10^−4^ M, [**L**]_0_ = 9.09 × 10^−5^ M and [*R*-AA]_0_ = [*S*-AA-*d*_n_]_0_ = 4.55 × 10^−5^ M. [CuCl_2_]_0_/[**L**]_0_/[*R*-AA]_0_/[*S*-AA-*d*_n_]_0_ = 1.2/1.0/0.5/0.5. [K_2_CO_3_]_0_ = 9.09 × 10^−5^ M, **L** = **L8**, AA= *R*-Orn/*S*-Orn-*d*_6_.


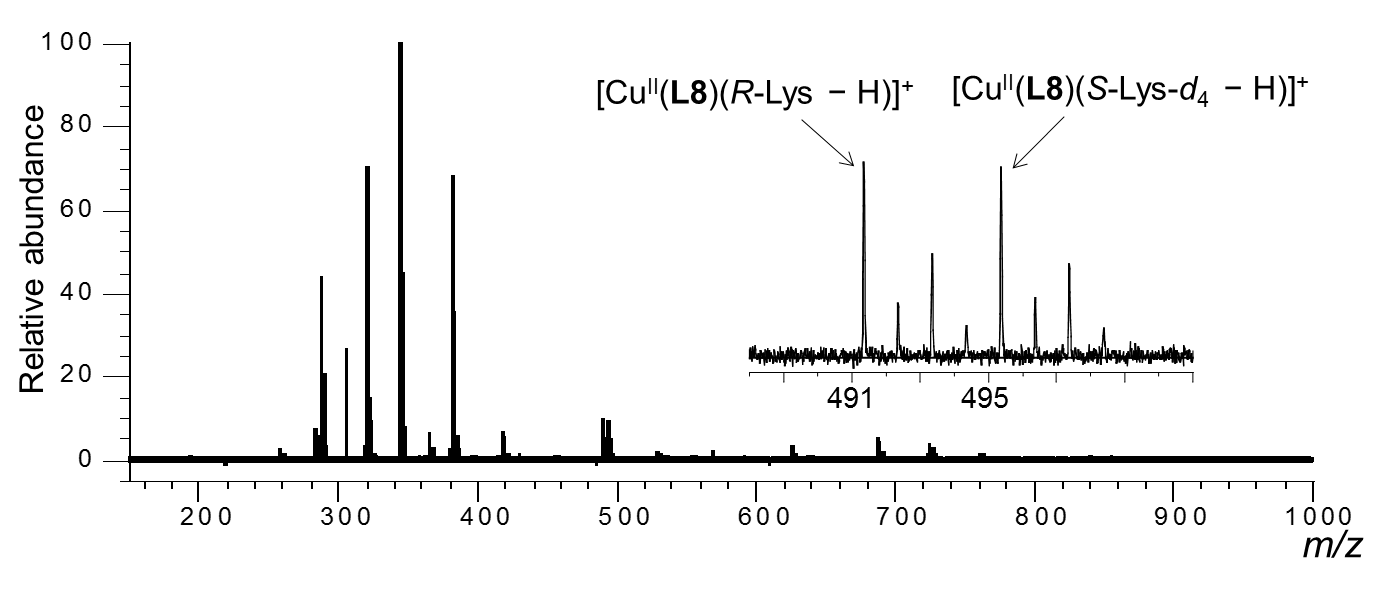


**Figure S40.** ESI mass spectra of the MS/EL method of CuCl_2_/**L**/*R*-AA/*S*-AA-*d*_n_- in water/methanol (1/10, v/v). [CuCl_2_]_0_ = 1.09 × 10^−4^ M, [**L**]_0_ = 9.09 × 10^−5^ M and [*R*-AA]_0_ = [*S*-AA-*d*_n_]_0_ = 4.55 × 10^−5^ M. [CuCl_2_]_0_/[**L**]_0_/[*R*-AA]_0_/[*S*-AA-*d*_n_]_0_ = 1.2/1.0/0.5/0.5. [K_2_CO_3_]_0_ = 9.09 × 10^−5^ M, **L** = **L8**, AA= *R*-Lys/*S*-Lys-*d*_4_.


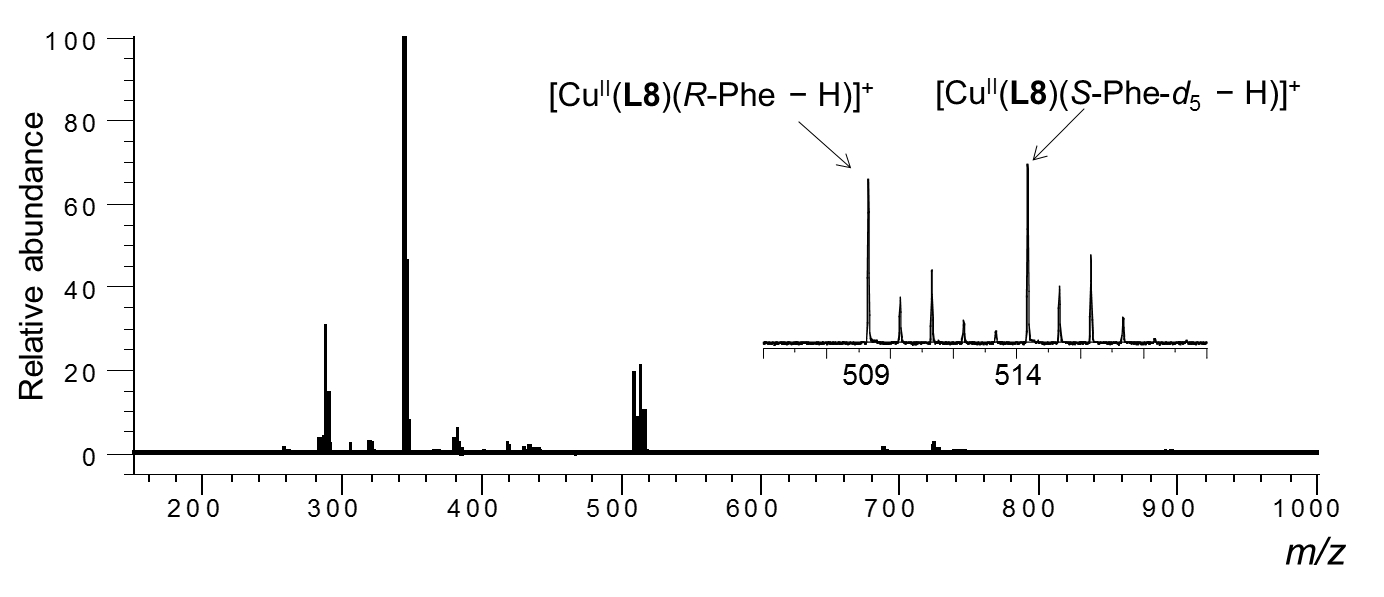


**Figure S41.** ESI mass spectra of the MS/EL method of CuCl_2_/**L**/*R*-AA/*S*-AA-*d*_n_- in water/methanol (1/10, v/v). [CuCl_2_]_0_ = 1.09 × 10^−4^ M, [**L**]_0_ = 9.09 × 10^−5^ M and [*R*-AA]_0_ = [*S*-AA-*d*_n_]_0_ = 4.55 × 10^−5^ M. [CuCl_2_]_0_/[**L**]_0_/[*R*-AA]_0_/[*S*-AA-*d*_n_]_0_ = 1.2/1.0/0.5/0.5. [K_2_CO_3_]_0_ = 9.09 × 10^−5^ M, **L** = **L8**, AA= *R*-Phe/*S*-Phe-*d*_5_.

.
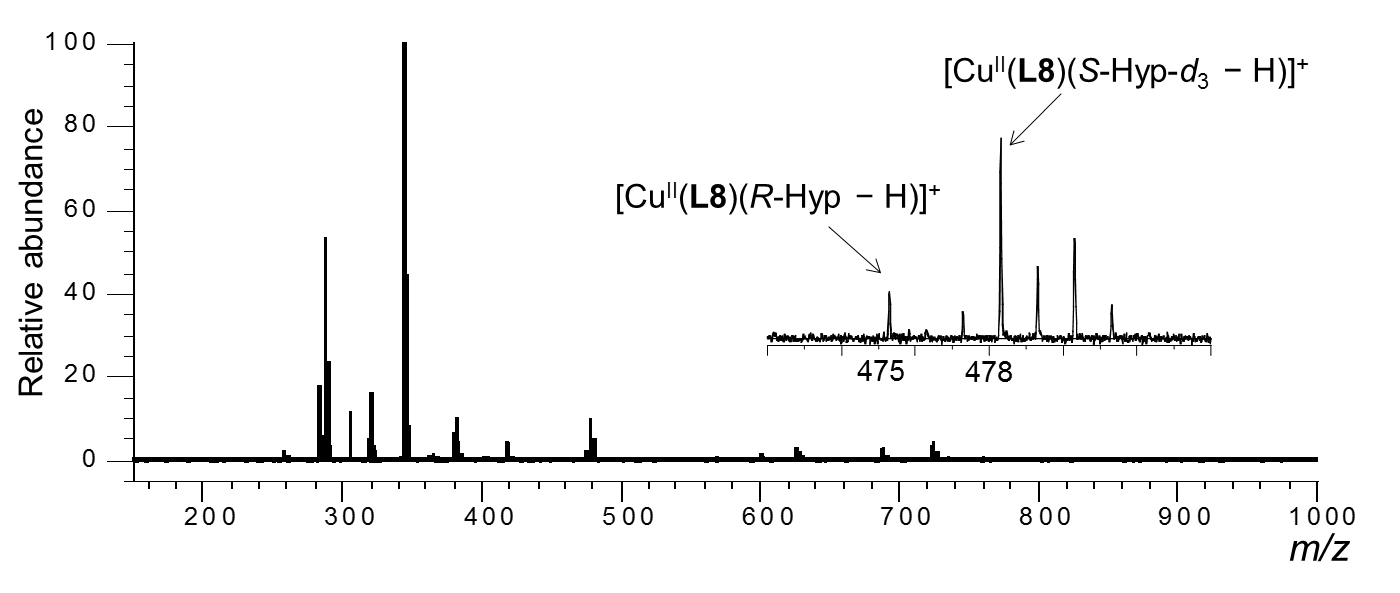


**Figure S42.** ESI mass spectra of the MS/EL method of CuCl_2_/**L**/*R*-AA/*S*-AA-*d*_n_- in water/methanol (1/10, v/v). [CuCl_2_]_0_ = 1.09 × 10^−4^ M, [**L**]_0_ = 9.09 × 10^−5^ M and [*R*-AA]_0_ = [*S*-AA-*d*_n_]_0_ = 4.55 × 10^−5^ M. [CuCl_2_]_0_/[**L**]_0_/[*R*-AA]_0_/[*S*-AA-*d*_n_]_0_ = 1.2/1.0/0.5/0.5. [K_2_CO_3_]_0_ = 9.09 × 10^−5^ M, **L** = **L8**, AA= *R*-Hyp/*S*-Hyp-*d*_3_.

# Deuterated amino acids

**Table S1.** The structure and D contents of deuterated amino acids





# DFT calculation of copper(II)-ligand complex [Cu(L)(MeOH)_2_]^2+^


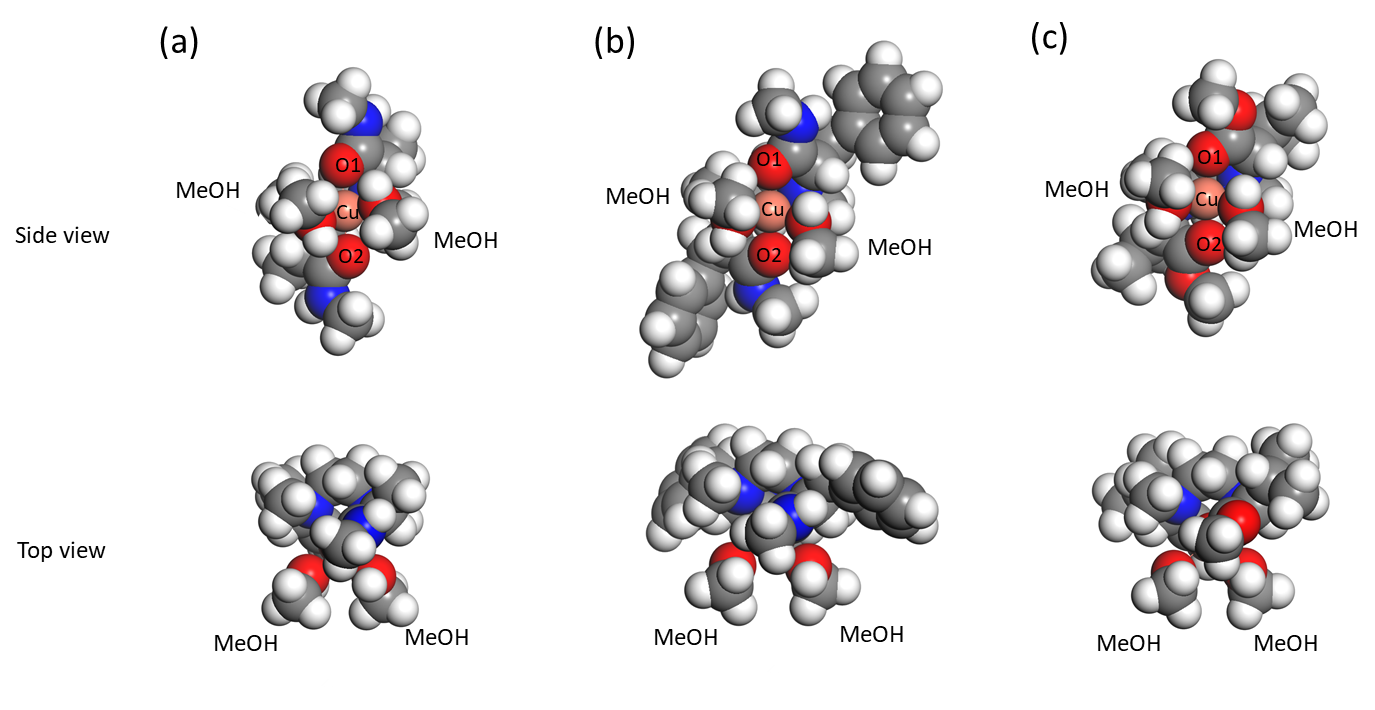


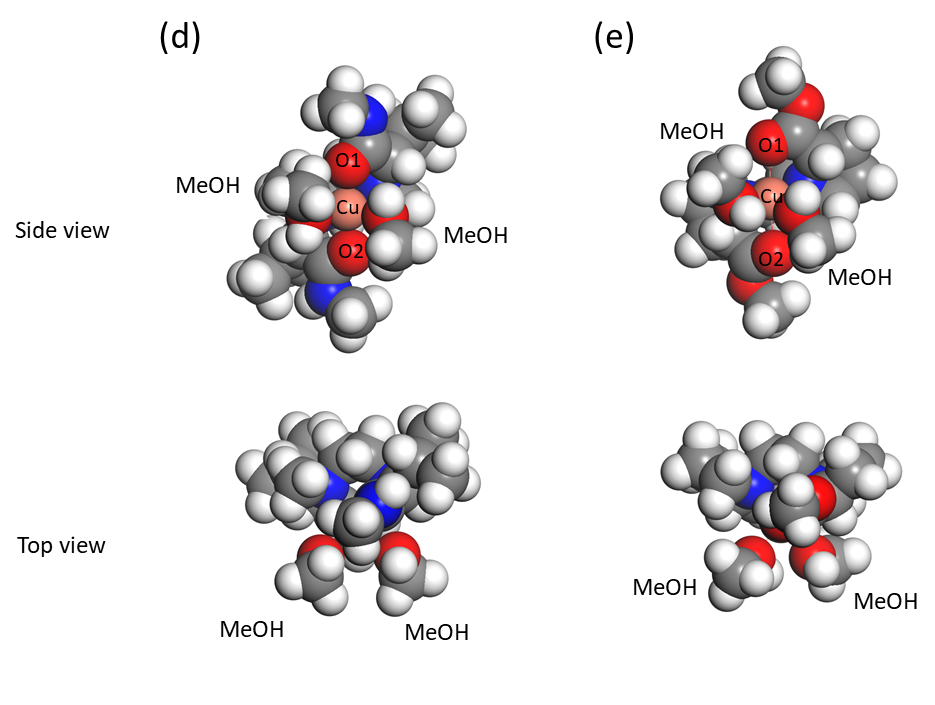


**Figure S43.** DFT calculation of the copper(II)-ligand complex [Cu(**L**)(MeOH)_2_]^2+^ in methanol. **L**: (a) **L2**, (b) **L4**, (c) **L5**, (d) **L6**, (e) **L7**.

# Bond length of Cu-O, Cu-N, and Cu-(MeOH) of copper(II)-ligand complex [Cu(L)(MeOH)_2_]^2+^ by DFT

**Table S2.** Bond length (Å) of Cu- Cu-O, Cu-N, and Cu-(MeOH) of copper(II)-ligand complex [Cu(**L**)(MeOH)_2_]^2+^ by DFT

| Complex ion | Cu-O1 | Cu-O2 | Cu-OMe1 | Cu-OMe2 | Cu-N1 | Cu-N2 |
| --- | --- | --- | --- | --- | --- | --- |
| [Cu(**L1**)(MeOH)_2_]^2+^ | 2.15 | 2.15 | 2.22 | 2.22 | 2.28 | 2.27 |
| [Cu(**L2**)(MeOH)_2_]^2+^ | 2.13 | 2.13 | 2.28 | 2.28 | 2.28 | 2.29 |
| [Cu(**L3**)(MeOH)_2_]^2+^ | 2.14 | 2.13 | 2.24 | 2.26 | 2.32 | 2.34 |
| [Cu(**L4**)(MeOH)_2_]^2+^ | 2.10 | 2.08 | 2.25 | 2.31 | 2.39 | 2.31 |
| [Cu(**L5**)(MeOH)_2_]^2+^ | 2.08 | 2.09 | 2.25 | 2.22 | 2.31 | 2.50 |
| [Cu(**L6**)(MeOH)_2_]^2+^ | 2.03 | 2.04 | 2.31 | 2.25 | 2.32 | 2.47 |
| [Cu(**L7**)(MeOH)_2_]^2+^ | 2.51 | 2.55 | 2.15 | 2.22 | 2.15 | 2.14 |
| [Cu(**L8**)(MeOH)_2_]^2+^ | 2.51 | 2.44 | 2.19 | 2.22 | 2.18 | 2.14 |

**N2**

**OMe2**

**OMe1**

**N1**

**O2**

**O1**

# Coordinates of copper(II)-ligand complex [Cu(L)(MeOH)_2_]^2+^ by DFT

**Table S3.** Final Coordinates (Angstroms) of [Cu^II^(**L1**)(MeOH)_2_]^2+^ complex calculated by DFT

ATOM X Y Z

1 O 1.230108 5.241101 26.225605

2 O -1.186360 3.690428 26.899695

3 H -0.597456 3.040821 26.492167

4 N -0.857349 6.938234 26.762265

5 O 1.278230 5.812985 24.069263

6 C 0.735559 5.828697 25.264595

7 C -0.068021 8.102401 27.235840

8 H -0.557073 9.038433 26.948140

9 H 0.903503 8.086407 26.746884

10 C -0.586523 6.587320 25.346190

11 C -2.302717 7.108494 27.020437

12 H -2.472766 7.271194 28.080966

13 H -2.832219 6.208597 26.716832

14 H -2.705929 7.965558 26.472725

15 C -2.380806 3.011511 27.343335

16 H -2.155271 2.329212 28.163956

17 H -2.817708 2.456105 26.512087

18 H -3.076335 3.774352 27.681235

19 C 2.498497 5.017613 23.909544

20 H 3.274156 5.413021 24.558345

21 H 2.764613 5.129567 22.865303

22 H 2.281890 3.981867 24.153149

23 C -0.688675 7.753788 24.364248

24 H -1.662892 8.231644 24.456440

25 H -0.594720 7.380708 23.346652

26 H 0.087145 8.498413 24.530775

27 O -1.241841 5.247100 29.736141

28 O 1.156754 3.661084 29.054411

29 H 0.555109 3.025327 29.464917

30 N 0.872088 6.915048 29.213472

31 O -1.289164 5.809201 31.895287

32 C -0.743031 5.823537 30.701797

33 C 0.108457 8.098318 28.744130

34 H 0.618404 9.022200 29.034592

35 H -0.862933 8.102258 29.233468

36 C 0.590844 6.562510 30.627339

37 C 2.321488 7.057301 28.960428

38 H 2.497664 7.224293 27.901560

39 H 2.830620 6.143829 29.258364

40 H 2.740863 7.901137 29.515873

41 C 2.333846 2.954836 28.605194

42 H 2.088676 2.276999 27.786526

43 H 2.762169 2.390904 29.434936

44 H 3.044313 3.701793 28.262894

45 C -2.519041 5.027053 32.049218

46 H -3.288887 5.433594 31.400463

47 H -2.785787 5.138367 33.093409

48 H -2.313381 3.989828 31.802402

49 C 0.709265 7.723155 31.614544

50 H 1.691743 8.184939 31.527484

51 H 0.605812 7.347548 32.630305

52 H -0.052860 8.481836 31.448427

53 Cu -0.001212 5.219669 27.984368

54 H 1.326656 5.800528 30.898903

55 H -1.336045 5.839338 25.073920

**Table S4.** Final Coordinates (Angstroms) of [Cu^II^(**L2**)(MeOH)_2_]^2+^ complex calculated by DFT

ATOM X Y Z

1 O 1.197965 5.239458 26.216214

2 O -1.166049 3.678318 26.860022

3 H -0.522507 3.241932 26.285817

4 N -0.834500 7.008621 26.735277

5 N 1.466307 5.960645 24.095111

6 H 1.122859 6.567487 23.367301

7 C 0.797187 5.918964 25.243337

8 C -0.057108 8.172216 27.225590

9 H -0.538648 9.108718 26.927903

10 H 0.925030 8.157762 26.757452

11 C -0.546614 6.661781 25.319916

12 C -2.281953 7.185445 26.969081

13 H -2.467915 7.336717 28.028380

14 H -2.812793 6.294018 26.644081

15 H -2.667114 8.055122 26.428807

16 C -2.113890 2.691974 27.325681

17 H -1.632103 1.980035 27.997633

18 H -2.543092 2.160131 26.476766

19 H -2.893047 3.228348 27.858505

20 C 2.692973 5.216773 23.812090

21 H 3.563299 5.779624 24.152073

22 H 2.761050 5.073323 22.737094

23 H 2.669124 4.253542 24.312712

24 C -0.682058 7.832619 24.347922

25 H -1.658005 8.298537 24.467430

26 H -0.634407 7.481928 23.317436

27 H 0.085923 8.590245 24.494178

28 O -1.200455 5.261482 29.744549

29 O 1.102333 3.649867 29.104647

30 H 0.439465 3.249756 29.683601

31 N 0.855739 7.002262 29.230123

32 N -1.428043 5.932937 31.885724

33 H -1.072691 6.524214 32.620449

34 C -0.779645 5.917084 30.724895

35 C 0.087255 8.171725 28.740011

36 H 0.575617 9.104901 29.037403

37 H -0.894852 8.164493 29.208219

38 C 0.566182 6.656750 30.645130

39 C 2.304598 7.168979 28.997206

40 H 2.492539 7.321776 27.938450

41 H 2.828933 6.272924 29.320165

42 H 2.696151 8.034594 29.539628

43 C 1.988872 2.612174 28.630490

44 H 1.461128 1.922207 27.970512

45 H 2.404607 2.064451 29.476169

46 H 2.785464 3.106304 28.082547

47 C -2.645150 5.176132 32.175423

48 H -3.524633 5.742725 31.866217

49 H -2.690992 5.007852 33.247901

50 H -2.627327 4.225238 31.651686

51 C 0.705090 7.828229 31.616420

52 H 1.682451 8.290658 31.495809

53 H 0.657482 7.480176 32.647784

54 H -0.060439 8.587862 31.468642

55 Cu -0.003422 5.293080 27.979432

56 H 1.306660 5.900195 30.921291

57 H -1.288795 5.906516 25.044918

**Table S5.** Final Coordinates (Angstroms) of [Cu^II^(**L3**)(MeOH)_2_]^2+^ complex calculated by DFT

ATOM X Y Z

1 O 1.015186 5.090006 26.294653

2 O -1.842080 3.723773 27.056156

3 N -1.032978 6.916750 26.650586

4 O 1.515601 6.031180 24.336132

5 C 0.721393 5.834590 25.358969

6 C -0.264682 8.059022 27.196667

7 H -0.728821 9.007970 26.912779

8 H 0.729314 8.052800 26.753268

9 C -0.634133 6.535105 25.276080

10 C -2.491385 7.101117 26.780680

11 H -2.995697 6.202313 26.434304

12 H -2.842937 7.964433 26.208000

13 H -2.745934 7.257963 27.825743

14 C -2.798796 2.930799 27.798462

15 H -2.379555 1.956016 28.048898

16 H -3.711158 2.804206 27.214434

17 H -3.019485 3.479187 28.707722

18 C 2.737019 5.236893 24.286721

19 H 3.216220 5.520635 23.357262

20 H 2.478473 4.182040 24.286485

21 H 3.367184 5.476270 25.138319

22 C -0.732161 7.656964 24.221898

23 H -0.038534 8.459984 24.466912

24 C -0.485644 7.215695 22.795573

25 H -1.738050 8.076660 24.296001

26 O -1.592155 5.248826 29.661158

27 O 0.884490 3.588723 29.085193

28 N 0.585925 6.850261 29.224222

29 O -1.733827 5.860275 31.799909

30 C -1.118953 5.820712 30.639445

31 C -0.148752 8.033706 28.718383

32 H 0.348100 8.960262 29.027116

33 H -1.142050 8.039289 29.164968

34 C 0.247594 6.495174 30.618712

35 C 2.043831 6.972173 29.036512

36 H 2.264886 7.142618 27.986878

37 H 2.524164 6.045706 29.344048

38 H 2.462856 7.804535 29.612389

39 C 2.007799 2.813380 28.611387

40 H 2.370005 3.299047 27.709516

41 H 1.697782 1.794539 28.376716

42 H 2.798883 2.797396 29.362140

43 C -3.003994 5.137803 31.878726

44 H -2.824148 4.082524 31.693215

45 H -3.698183 5.546142 31.149849

46 H -3.358486 5.306880 32.889533

47 C 0.390093 7.645462 31.650387

48 H -0.537349 8.212148 31.713765

49 C 0.823548 7.209689 33.026372

50 H 1.148015 8.327788 31.261772

51 Cu -0.327519 5.121010 27.944112

52 H 0.940244 5.696070 30.895468

53 H -1.322362 5.736248 24.989850

54 C 2.042055 6.543217 33.216637

55 C 2.476048 6.199639 34.494915

56 C 1.698374 6.519147 35.608593

57 C 0.484533 7.182281 35.430837

58 C 0.051379 7.521455 34.150511

59 C -1.042880 6.041467 22.274148

60 C -0.731991 5.624084 20.981046

61 C 0.116468 6.384681 20.177219

62 C 0.644179 7.577033 20.671425

63 C 0.342277 7.984287 21.969202

64 H 2.669345 6.302474 32.366901

65 H 3.422179 5.690067 34.623373

66 H 2.035549 6.259444 36.603805

67 H -0.123580 7.440258 36.287936

68 H -0.890025 8.039586 34.020423

69 H -1.706684 5.435116 22.876645

70 H -1.157268 4.704997 20.599214

71 H 0.356626 6.052788 19.175091

72 H 1.299144 8.179912 20.055826

73 H 0.780715 8.895544 22.356044

74 H 0.399652 3.066046 29.736551

75 H -1.539277 3.208113 26.297960

**Table S6.** Final Coordinates (Angstroms) of [Cu^II^(**L4**)(MeOH)_2_]^2+^ complex calculated by DFT

ATOM X Y Z

1 O 0.895752 5.039247 26.355279

2 O -1.828285 3.598416 27.152931

3 N -1.086588 6.915778 26.621920

4 N 1.588037 5.882059 24.407574

5 C 0.687879 5.791881 25.376302

6 C -0.350225 8.067991 27.185816

7 H -0.840424 9.011403 26.919880

8 H 0.644022 8.098395 26.743739

9 C -0.655718 6.528743 25.259418

10 C -2.545774 7.071347 26.739132

11 H -3.031174 6.152794 26.417442

12 H -2.919655 7.912858 26.145615

13 H -2.803262 7.249240 27.780550

14 C -2.645634 2.705928 27.942022

15 H -2.131770 1.759391 28.117350

16 H -3.592690 2.522118 27.432799

17 H -2.826230 3.205201 28.888622

18 C 2.794120 5.063975 24.392781

19 H 3.387389 5.349996 23.529206

20 H 2.535353 4.007525 24.325311

21 H 3.373336 5.226361 25.299808

22 C -0.733634 7.674131 24.221944

23 H -0.052889 8.477317 24.501710

24 C -0.464508 7.290026 22.781493

25 H -1.741565 8.087145 24.290532

26 O -1.590389 5.223445 29.702322

27 O 0.993381 3.548409 29.031777

28 N 0.529365 6.850662 29.196232

29 N -1.777478 5.850418 31.854537

30 C -1.123626 5.820506 30.696922

31 C -0.239257 8.017431 28.707447

32 H 0.224215 8.953413 29.038253

33 H -1.234529 7.977501 29.146881

34 C 0.250140 6.509364 30.608853

35 C 1.975828 6.984369 28.939306

36 H 2.149021 7.088666 27.873068

37 H 2.485582 6.085765 29.278583

38 H 2.402400 7.859325 29.440367

39 C 1.952255 2.711519 28.346633

40 H 2.154058 3.182354 27.389748

41 H 1.543659 1.713418 28.189110

42 H 2.874441 2.644303 28.926984

43 C -3.057810 5.189167 32.081891

44 H -2.916324 4.164046 32.426983

45 H -3.621882 5.179559 31.154692

46 H -3.601574 5.750761 32.836993

47 C 0.447559 7.686003 31.602226

48 H -0.451788 8.300542 31.642815

49 C 0.866590 7.272197 32.992524

50 H 1.234839 8.324231 31.195219

51 Cu -0.376238 5.097081 27.995161

52 H 0.964674 5.723827 30.860313

53 H -1.351068 5.744183 24.951319

54 C 2.009719 6.482117 33.184633

55 C 2.423366 6.122999 34.463238

56 C 1.697364 6.546980 35.576825

57 C 0.565299 7.340101 35.401496

58 C 0.152628 7.700409 34.118049

59 C -1.015234 6.138239 22.201264

60 C -0.683964 5.774165 20.898530

61 C 0.178461 6.566171 20.140617

62 C 0.703461 7.733141 20.691595

63 C 0.382236 8.089292 22.001454

64 H 2.592568 6.153264 32.333528

65 H 3.309277 5.514606 34.591016

66 H 2.013903 6.267085 36.572560

67 H 0.001575 7.679632 36.260175

68 H -0.719338 8.331304 33.991082

69 H -1.686478 5.507783 22.769241

70 H -1.105876 4.874317 20.470547

71 H 0.428130 6.280843 19.127070

72 H 1.366244 8.362538 20.112360

73 H 0.814426 8.984558 22.431343

74 H 0.573406 3.029559 29.729401

75 H -1.546789 3.136660 26.352488

76 H -1.337732 6.287762 32.649939

77 H 1.412136 6.479356 23.614575

**Table S7.** Final Coordinates (Angstroms) of [Cu^II^(**L5**)(MeOH)_2_]^2+^ complex calculated by DFT

ATOM X Y Z

1 O 1.117995 5.185168 26.329153

2 O -1.735851 3.669707 27.103378

3 N -0.926436 6.967067 26.663593

4 O 1.585610 5.987573 24.307867

5 C 0.809081 5.872670 25.352774

6 C -0.149186 8.092976 27.239511

7 H -0.598718 9.048105 26.951415

8 H 0.851616 8.072125 26.816161

9 C -0.536550 6.589021 25.273397

10 C -2.383808 7.182623 26.787462

11 H -2.912097 6.308875 26.413031

12 H -2.707537 8.071258 26.234450

13 H -2.644952 7.322595 27.833616

14 C -2.574753 2.817002 27.916210

15 H -2.081351 1.862979 28.104221

16 H -3.528570 2.649612 27.414541

17 H -2.738706 3.338741 28.852765

18 C 2.831362 5.228702 24.322308

19 H 3.325684 5.494507 23.396672

20 H 2.607230 4.167587 24.359321

21 H 3.427986 5.526699 25.178864

22 C -0.711830 7.654459 24.151404

23 C 0.282111 8.816086 24.120211

24 H -1.698893 8.088278 24.335443

25 O -1.400948 5.291200 29.631003

26 O 1.037008 3.623003 28.879632

27 N 0.716256 6.968871 29.322173

28 O -1.613498 5.764987 31.785545

29 C -0.962966 5.823546 30.651440

30 C -0.057853 8.092563 28.764501

31 H 0.393024 9.051612 29.038060

32 H -1.054072 8.081060 29.202618

33 C 0.373552 6.574230 30.704971

34 C 2.160052 7.009834 29.029339

35 H 2.321049 6.999279 27.954038

36 H 2.628718 6.124377 29.452136

37 H 2.641926 7.905213 29.432588

38 C 1.997668 2.714019 28.290765

39 H 2.295419 3.135658 27.336777

40 H 1.543385 1.735853 28.139950

41 H 2.865728 2.625611 28.944225

42 C -2.844682 4.983463 31.809104

43 H -2.597574 3.931926 31.693400

44 H -3.503660 5.319216 31.014478

45 H -3.276047 5.174749 32.784861

46 C 0.393458 7.653654 31.852516

47 H -0.588596 8.134071 31.867151

48 C 1.442639 8.751859 31.684563

49 Cu -0.249345 5.147955 27.910495

50 H 1.103274 5.803173 30.970482

51 H -1.233000 5.786746 25.007116

52 H 0.603638 3.170009 29.613880

53 H -1.486203 3.181992 26.307471

54 C 0.639793 6.980677 33.216858

55 C -0.785825 6.965856 22.775799

56 H 0.562452 7.727916 34.009564

57 H -0.065310 6.180353 33.430650

58 H 1.652896 6.567885 33.242918

59 H 2.452970 8.340632 31.669169

60 H 1.298100 9.359295 30.794241

61 H 1.377443 9.421314 32.543988

62 H 0.196164 9.469258 24.986896

63 H 0.072483 9.420937 23.235400

64 H 1.315650 8.478274 24.042989

65 H -1.143962 7.682148 22.034055

66 H -1.471646 6.114920 22.792053

67 H 0.194375 6.612683 22.454199

**Table S8.** Final Coordinates (Angstroms) of [Cu^II^(**L6**)(MeOH)_2_]^2+^ complex calculated by DFT

ATOM X Y Z

1 O 1.132714 5.215996 26.361286

2 O -1.685044 3.677104 27.099452

3 N -0.915165 6.944979 26.692470

4 N 1.510217 5.738885 24.224586

5 C 0.779977 5.826773 25.323685

6 C -0.187030 8.098417 27.263188

7 H -0.676886 9.032076 26.969888

8 H 0.811665 8.116101 26.837004

9 C -0.534271 6.607027 25.294720

10 C -2.377134 7.068944 26.850324

11 H -2.847607 6.155640 26.493680

12 H -2.773776 7.925001 26.294535

13 H -2.618496 7.196756 27.903129

14 C -2.539896 2.842269 27.909518

15 H -2.060092 1.884410 28.115443

16 H -3.492244 2.676200 27.403189

17 H -2.705939 3.375217 28.839867

18 C 2.741313 4.960369 24.176698

19 H 3.231708 5.177258 23.231879

20 H 2.528699 3.892166 24.243026

21 H 3.382145 5.256360 25.006455

22 C -0.686515 7.756028 24.244184

23 C 0.392669 8.846120 24.202327

24 H -1.619294 8.243035 24.538593

25 O -1.258148 5.176186 29.648478

26 O 1.178420 3.628080 28.941677

27 N 0.726820 7.011257 29.339853

28 N -1.754199 5.831924 31.710269

29 C -0.936246 5.831470 30.668239

30 C -0.088568 8.104087 28.786479

31 H 0.325794 9.080366 29.055231

32 H -1.082257 8.050651 29.226481

33 C 0.402865 6.593929 30.720873

34 C 2.162546 7.090659 29.026006

35 H 2.311345 7.034653 27.950641

36 H 2.672665 6.245981 29.480406

37 H 2.613967 8.018938 29.383160

38 C 2.068216 2.701216 28.281075

39 H 2.350392 3.155329 27.336218

40 H 1.567254 1.750034 28.100019

41 H 2.956625 2.540772 28.894004

42 C -2.973098 5.032107 31.729478

43 H -2.733492 3.976087 31.604167

44 H -3.642038 5.340565 30.926131

45 H -3.457830 5.184667 32.689177

46 C 0.453956 7.643930 31.883001

47 H -0.531432 8.110761 31.955062

48 C 1.453985 8.788613 31.708125

49 Cu -0.142798 5.159993 27.956960

50 H 1.140572 5.823386 30.956116

51 H -1.267473 5.854755 24.991199

52 H 0.719948 3.167192 29.656010

53 H -1.415761 3.175756 26.319246

54 H -1.545259 6.392610 32.519763

55 H 1.253809 6.269326 23.409114

56 C 0.777875 6.939471 33.217907

57 C -0.928649 7.202807 22.824733

58 H 0.631448 7.635422 34.045606

59 H 0.175335 6.054003 33.415911

60 H 1.822918 6.621831 33.222945

61 H 2.481110 8.428588 31.639381

62 H 1.246480 9.412398 30.840940

63 H 1.394791 9.433103 32.587402

64 H 0.438775 9.440882 25.110950

65 H 0.161790 9.528715 23.382551

66 H 1.389765 8.444148 24.012441

67 H -1.490521 7.937244 22.246594

68 H -1.493492 6.268136 22.838031

69 H -0.002147 7.038255 22.269163

**Table S9.** Final Coordinates (Angstroms) of [Cu^II^(**L7**)(MeOH)_2_]^2+^ complex calculated by DFT

ATOM X Y Z

1 O 0.233872 0.213637 -0.505720

2 O 3.640405 0.251073 0.328218

3 H 3.487999 1.203367 0.423172

4 N 1.414622 -2.226797 0.156131

5 O -0.583550 -0.744279 -2.376489

6 C 0.234700 -0.676036 -1.345868

7 C 0.146113 -2.706028 0.782208

8 H -0.076806 -3.731170 0.478644

9 H -0.679743 -2.079899 0.452113

10 C 1.243829 -1.800640 -1.263168

11 C 2.396787 -3.342188 0.031589

12 H 2.361084 -3.975343 0.912951

13 H 3.394417 -2.913529 -0.043037

14 C 5.016816 -0.029522 0.700623

15 H 5.177135 0.196031 1.756009

16 H 5.685750 0.566863 0.082516

17 H 5.187153 -1.085790 0.518179

18 C -1.573290 0.321491 -2.551418

19 H -1.122842 1.108351 -3.150582

20 H -1.887772 0.700798 -1.585551

21 H -2.393885 -0.147621 -3.082547

22 C 1.093250 -3.101939 -2.055406

23 H 1.395799 -2.970028 -3.091551

24 H 0.058592 -3.443576 -2.048194

25 O 3.188319 -1.632344 3.121284

26 O 1.810904 1.675165 1.853126

27 H 2.327088 1.986699 2.609739

28 N 0.469533 -1.233866 2.734855

29 O 2.772136 -2.326533 5.230890

30 C 2.453272 -1.740528 4.094698

31 C 0.273627 -2.649946 2.289029

32 H -0.617627 -3.084620 2.743884

33 H 1.126578 -3.243647 2.607823

34 C 1.075549 -1.127683 4.091412

35 C -0.844949 -0.569586 2.960612

36 H -1.536293 -0.805287 2.156805

37 H -0.676495 0.505463 2.967607

38 C 0.818017 2.678020 1.515477

39 H 1.316246 3.573624 1.145687

40 H 0.216269 2.915344 2.390891

41 H 0.196301 2.251105 0.736933

42 C 4.124794 -2.856649 5.385246

43 H 4.018419 -3.730676 6.018085

44 H 4.727974 -2.095111 5.873134

45 H 4.539202 -3.117532 4.417756

46 C -0.024447 -1.574059 5.062118

47 H 0.116257 -1.132212 6.046531

48 H -0.029030 -2.656920 5.179341

49 Cu 1.878111 -0.476818 1.317389

50 C -1.311159 -1.069240 4.351648

51 C 2.012981 -4.077570 -1.278787

52 H 2.910446 -4.319700 -1.846111

53 H 1.496263 -5.013697 -1.069306

54 H -2.036757 -1.875135 4.253329

55 H -1.788756 -0.259992 4.901672

56 H 1.262093 -0.060645 4.254323

57 H 2.188469 -1.331287 -1.553178

**Table S10.** Final Coordinates (Angstroms) of [Cu^II^(**L8**)(MeOH)_2_]^2+^ complex calculated by DFT

ATOM X Y Z

1 O 0.215967 0.158645 -0.389633

2 O 3.376857 0.358747 0.072234

3 H 3.328929 1.286664 0.345256

4 N 1.474057 -2.232408 0.219898

5 N -0.596998 -0.737272 -2.311168

6 H -0.512743 -1.485116 -2.984833

7 C 0.242143 -0.716237 -1.277397

8 C 0.220956 -2.764452 0.826911

9 H 0.063073 -3.808367 0.546564

10 H -0.630347 -2.194835 0.461470

11 C 1.302743 -1.797846 -1.195169

12 C 2.503366 -3.302127 0.100249

13 H 2.478600 -3.948265 0.972636

14 H 3.482957 -2.830862 0.054263

15 C 4.769772 -0.014831 -0.006017

16 H 5.215671 -0.058030 0.987996

17 H 5.300403 0.708525 -0.622865

18 H 4.815927 -0.993343 -0.473794

19 C -1.626189 0.270291 -2.537115

20 H -1.453294 0.767245 -3.491572

21 H -1.592051 0.994817 -1.731736

22 H -2.605700 -0.207275 -2.560102

23 C 1.223479 -3.097595 -2.003918

24 H 1.532590 -2.948891 -3.036981

25 H 0.209616 -3.500951 -2.007439

26 O 3.132834 -1.339268 3.286880

27 O 2.032687 1.690052 2.052154

28 H 2.617185 1.864168 2.802246

29 N 0.393385 -1.235645 2.764191

30 N 2.683294 -2.324038 5.295081

31 H 1.968358 -2.498307 5.986367

32 C 2.350833 -1.611813 4.217499

33 C 0.301730 -2.666168 2.338308

34 H -0.570600 -3.153145 2.777247

35 H 1.184315 -3.196036 2.691190

36 C 0.937073 -1.072362 4.144939

37 C -0.966560 -0.645266 2.918296

38 H -1.608159 -0.953866 2.097911

39 H -0.868798 0.438696 2.887462

40 C 1.268260 2.876894 1.731624

41 H 1.940449 3.704856 1.510097

42 H 0.614026 3.137215 2.562190

43 H 0.677364 2.634992 0.854191

44 C 4.015477 -2.839248 5.572414

45 H 3.966604 -3.918153 5.715034

46 H 4.410417 -2.379418 6.478196

47 H 4.664436 -2.609542 4.733816

48 C -0.180934 -1.576022 5.066105

49 H -0.109550 -1.141335 6.062109

50 H -0.150528 -2.661483 5.166993

51 Cu 1.798755 -0.411809 1.372401

52 C -1.460239 -1.124195 4.308541

53 C 2.181640 -4.037968 -1.227319

54 H 3.098573 -4.222669 -1.785164

55 H 1.709735 -5.002051 -1.041807

56 H -2.167760 -1.947679 4.218255

57 H -1.963592 -0.310545 4.827873

58 H 1.045453 0.005629 4.294985

59 H 2.230335 -1.287905 -1.468580
